# Donor–Acceptor Pyridine-Based Conjugated Microporous Polymers for Visible-Light-Driven Reactive Oxygen Species Generation and Photocatalytic Dye Degradation

**DOI:** 10.1021/acspolymersau.6c00054

**Published:** 2026-06-01

**Authors:** Shimaa Abdelnaser, M. Rashad, Wei-Lung Tseng, Shiao-Wei Kuo, Ahmed F. M. EL-Mahdy

**Affiliations:** † Department of Materials and Optoelectronic Science, 34874 National Sun Yat-Sen University, Kaohsiung 80424, Taiwan; ‡ Chemistry Department, Faculty of Science, Assiut University, Assiut 71516, Egypt; § Physics Department, Faculty of Science, 125900 University of Tabuk, Tabuk 71491, Saudi Arabia; ∥ Department of Chemistry, National Sun Yat-sen University, Kaohsiung 80424, Taiwan

**Keywords:** Conjugated microporous polymers, D−A polymers, Pyridine-based frameworks, Reactive oxygen species, Photocatalytic dye degradation

## Abstract

Conjugated microporous polymers (CMPs) are attractive organic photocatalysts because their porosity, extended π conjugation, and tunable donor–acceptor (D–A) architectures can promote light harvesting, charge separation, and substrate transport. Herein we report two pyridine-based donor–acceptor CMPs, BTPP-TPA and BTPP-TPT, synthesized through Suzuki coupling of a tetrabrominated pyridine-containing building block (BTPP) with triphenylamine (TPA)- or triphenyltriazine (TPT)-based boronate monomers. Both CMPs exhibit high thermal stability, permanent porosity, broad visible-light absorption, and favorable electronic structures for photocatalytic applications. Notably, the more planar and nitrogen-rich BTPP-TPT framework shows enhanced interfacial charge transport, stronger dye–framework interactions, and superior photocatalytic activity relative to BTPP-TPA. BTPP-TPT delivered adsorption efficiencies of 98.29% for rhodamine B (RhB) and 98.24% for methylene blue (MB) within 90 min and achieved visible-light-driven photodegradation rate constants of 2.6 × 10^–2^ min^–1^ for RhB and 1.7 × 10^–2^ min^–1^ for MB. Spectroscopic and electrochemical studies, together with molecular orbital calculations, indicate that D–A polarization and linker planarity govern charge separation and reactive oxygen species generation in these frameworks. This work highlights how molecular-level engineering of pyridine-based CMPs can regulate porosity, electronic structure, and photocatalytic function, providing an effective strategy for metal-free polymer photocatalysts for wastewater remediation.

## Graphical abstract

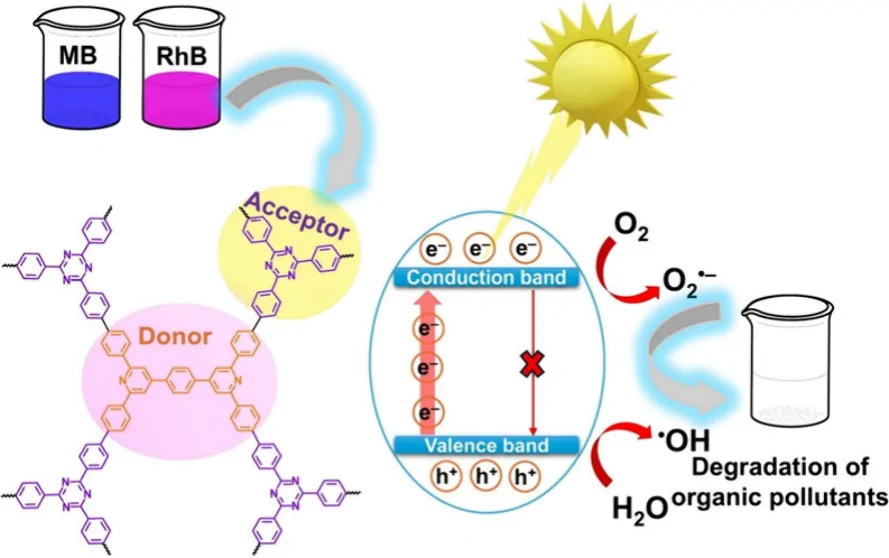

## 1 Introduction

Photocatalysis has emerged as an environmentally benign strategy for solar-energy conversion and wastewater remediation, in which the intrinsic optical, electronic, and surface properties of the catalyst strongly determine overall catalytic efficiency.^1^ In particular, semiconductor photocatalysts have attracted considerable attention because they can use light energy to oxidatively decompose persistent organic pollutants into less harmful products such as CO_2_, H_2_O, and inorganic ions.^2^ Water is an indispensable resource for biological, environmental, and industrial systems; however, the deterioration of freshwater quality has become a serious global challenge.^3,4^ Rapid industrialization has intensified water pollution, especially through the discharge of nonbiodegradable organic dyes from textile, leather, cosmetic, pharmaceutical, printing, and related industries.^5,6^ These dyes are highly soluble, chemically stable, and resistant to natural degradation, allowing them to persist in aquatic environments.^7^ Even at low concentrations, many organic dyes can cause ecological damage and pose risks to human health, including skin irritation, respiratory problems, and potentially carcinogenic effects through contaminated water and food chains.^6,8–10^ Therefore, developing rapid, efficient, and sustainable methods for removing dye pollutants from water is urgently required. Various physical, chemical, and biological approaches, including adsorption, coagulation, membrane filtration, ion exchange, sedimentation, biodegradation, and catalysis, have been explored for dye removal.^11^ Among them, advanced oxidation processes (AOPs) based on photocatalysis are particularly promising because they can generate reactive oxygen species (ROS) under light irradiation.^12^ During photocatalysis, photoexcited electrons and holes are produced in the catalyst and subsequently react with dissolved O_2_, H_2_O, or surface hydroxyl groups to form ROS such as hydroxyl radicals (^•^OH), superoxide radicals (O_2_^•–^), hydrogen peroxide (H_2_O_2_), and singlet oxygen (^1^O_2_).^13,14^ These highly active species can attack dye molecules and promote their oxidative degradation, thereby improving water purification efficiency. Moreover, ROS-based oxidation is attractive from a sustainability viewpoint because it uses abundant oxygen and water as reactants and can reduce the need for harsh chemical oxidants.^15,16^ Inorganic semiconductors, including metal oxides and metal sulfides, have been widely studied for photocatalytic dye degradation.^17^ However, their practical application is often restricted by wide band gaps, limited visible-light absorption, low surface areas, possible secondary toxicity, high production cost, and poor structural flexibility.^12,18^ In addition, the use of nonrenewable or metal-based components raises sustainability concerns.^19^ Consequently, metal-free organic semiconductors have emerged as promising alternatives because their molecular structures, band gaps, porosity, and surface functionalities can be rationally tailored for visible-light-driven photocatalytic degradation.

In recent years, conjugated microporous polymers (CMPs), an important subclass of porous organic polymers (POPs), have rapidly emerged as promising materials for adsorption and photocatalytic applications.^11^ Compared with conventional porous materials, CMPs offer several advantages, including high surface area, robust covalent frameworks, permanent porosity, extended π conjugation, tunable electronic structures, and facile structural functionalization.^20–25^ These features make CMPs attractive platforms for addressing water contamination from industrial dyes.^26^ However, the adsorption performance of many CMPs is often limited by their intrinsic hydrophobicity, insufficient accessible binding sites, and restricted pore accessibility.^27^ To overcome these limitations, the incorporation of heteroatoms such as N, O, and S into CMP backbones has been widely adopted as an effective strategy to improve host–guest interactions, surface polarity, adsorption efficiency, and selectivity.^28^ Despite these advantages, the photocatalytic application of CMPs is still challenged by limited charge-carrier mobility and strong exciton binding energies, which can accelerate electron–hole recombination and reduce photocatalytic efficiency.^29,30^ One effective approach to address these issues is the construction of donor–acceptor (D–A) CMPs with extended conjugated backbones and internal porous structures. D–A architectures can promote photoinduced intramolecular charge transfer, reduce exciton recombination, and enhance charge separation and transport, thereby improving photocatalytic activity.^31,32^ In addition, the incorporation of different donor and acceptor units into the polymer skeleton enables precise modulation of the optical band gap and electronic structure, which is particularly beneficial for visible-light-driven photocatalytic degradation of organic pollutants.^33,34^ Therefore, developing environmentally stable D–A CMPs with efficient adsorption capability, strong visible-light absorption, and improved charge-transfer properties remains an important challenge for photocatalytic wastewater treatment.

The rational engineering of nitrogen-containing heterocycles within CMP networks is especially important for enhancing both adsorption and photocatalytic performance.^35,36^ Pyridine is a representative nitrogen-containing aromatic heterocycle in which the pyridinic nitrogen can regulate electron distribution, improve charge-transfer behavior, and provide Lewis-basic interaction sites for organic molecules.^37^ Therefore, pyridine-derived building blocks are attractive components for the design of photocatalytically active CMPs. In addition, triphenylamine (TPA) and triphenyltriazine (TPT) derivatives are widely used photoactive units because of their aromatic structures, redox activity, and favorable charge-transport properties.^38,39^ TPA commonly serves as an electron-rich donor unit, whereas TPT possesses a more planar, nitrogen-rich, and electron-deficient triazine core, making it suitable as an acceptor segment.^40^ The combination of pyridine-containing linkers with TPA or TPT units can therefore generate D–A CMP frameworks with tunable electronic structures, enhanced charge separation, and improved photocatalytic activity.^41^ In this work, rhodamine B (RhB) and methylene blue (MB) were selected as representative cationic dye pollutants because they are widely used in textiles, printing, cosmetics, pharmaceuticals, and related industries.^42,43^ These dyes are chemically stable, highly soluble, and resistant to biodegradation, allowing them to persist in aquatic environments even at low concentrations.^44^ Their accumulation can cause serious environmental and health concerns, including irritation, respiratory problems, toxicity, mutagenicity, and potential carcinogenic effects.^42,45,46^ Therefore, RhB and MB are suitable model pollutants for evaluating the adsorption and visible-light-driven photocatalytic degradation performance of the designed CMP photocatalysts.

Effective CMP photocatalysts require careful selection of photoactive precursors and covalent linkages. Therefore, we designed two BTPP-based CMPs, BTPP-TPA and BTPP-TPT, through Suzuki–Miyaura coupling to construct donor–acceptor architectures with extended π conjugation for enhanced photocatalytic performance. BTPP-TPA CMP was synthesized by coupling 1,4-bis­(2,6-bis­(4-bromophenyl)­pyridin-4-yl)­benzene (BTPP-4Br) (Scheme S1 and Figures S1–S3) with tris­(4-(4,4,5,5-tetramethyl-1,3,2-dioxaborolan-2-yl)­phenyl)­amine (TPA-3BO) (Scheme S2 and Figures S4–S6), whereas BTPP-TPT CMP was prepared by coupling BTPP-4Br with 2,4,6-tris­(4-(4,5-dimethyl-1,3,2-dioxaborolan-2-yl)­phenyl)-1,3,5-triazine (TPT-3BO) (Scheme S3 and Figures S7–S9). Both synthesized CMPs possess a conjugated structure (Schemes S4, S5, and 1). In these frameworks, TPA acts as an electron-donating unit, while TPT provides a more planar and electron-accepting triazine-based segment, enabling modulation of the donor–acceptor character and charge-transfer behavior.^31,47^ The BTPP unit was selected as the central pyridine-containing building block because it offers both structural and electronic advantages for the construction of donor–acceptor CMP photocatalysts. Unlike simple pyridine derivatives, BTPP-4Br contains four reactive bromophenyl sites, enabling efficient Suzuki coupling with trifunctional TPA-3BO and TPT-3BO monomers to form highly cross-linked porous polymer networks. In addition, its rigid bis­(pyridylphenyl)­benzene skeleton extends π conjugation throughout the framework, which is beneficial for visible-light absorption, charge delocalization, and interfacial charge transport. The pyridine nitrogen sites also provide electronically active interaction centers that can enhance dye-framework affinity and facilitate photoinduced charge redistribution. Moreover, DFT calculations indicate that BTPP can play different electronic roles depending on the paired comonomer, acting as an electron-accepting segment in BTPP-TPA CMP and as an electron-donating segment in BTPP-TPT CMP. This electronic versatility enables systematic modulation of the donor–acceptor character, HOMO/LUMO distribution, and photocatalytic behavior. Therefore, BTPP was chosen as a multifunctional pyridine-based linker that integrates cross-linking capability, extended conjugation, nitrogen-rich active sites, and tunable donor–acceptor interactions, distinguishing it from simpler pyridine-containing alternatives. Herein we report the successful fabrication of BTPP-based CMPs with a high specific surface area (up to 521 m^2^ g^–1^), excellent thermal robustness (char yield of 68.54% at 800 °C), and strong chemical resistance. These CMPs were then applied to the adsorption and photocatalytic degradation of the cationic dyes RhB and MB. Notably, the adsorption behavior and photocatalytic performance of these materials toward organic dye removal have not been previously investigated.

**Scheme 1:**
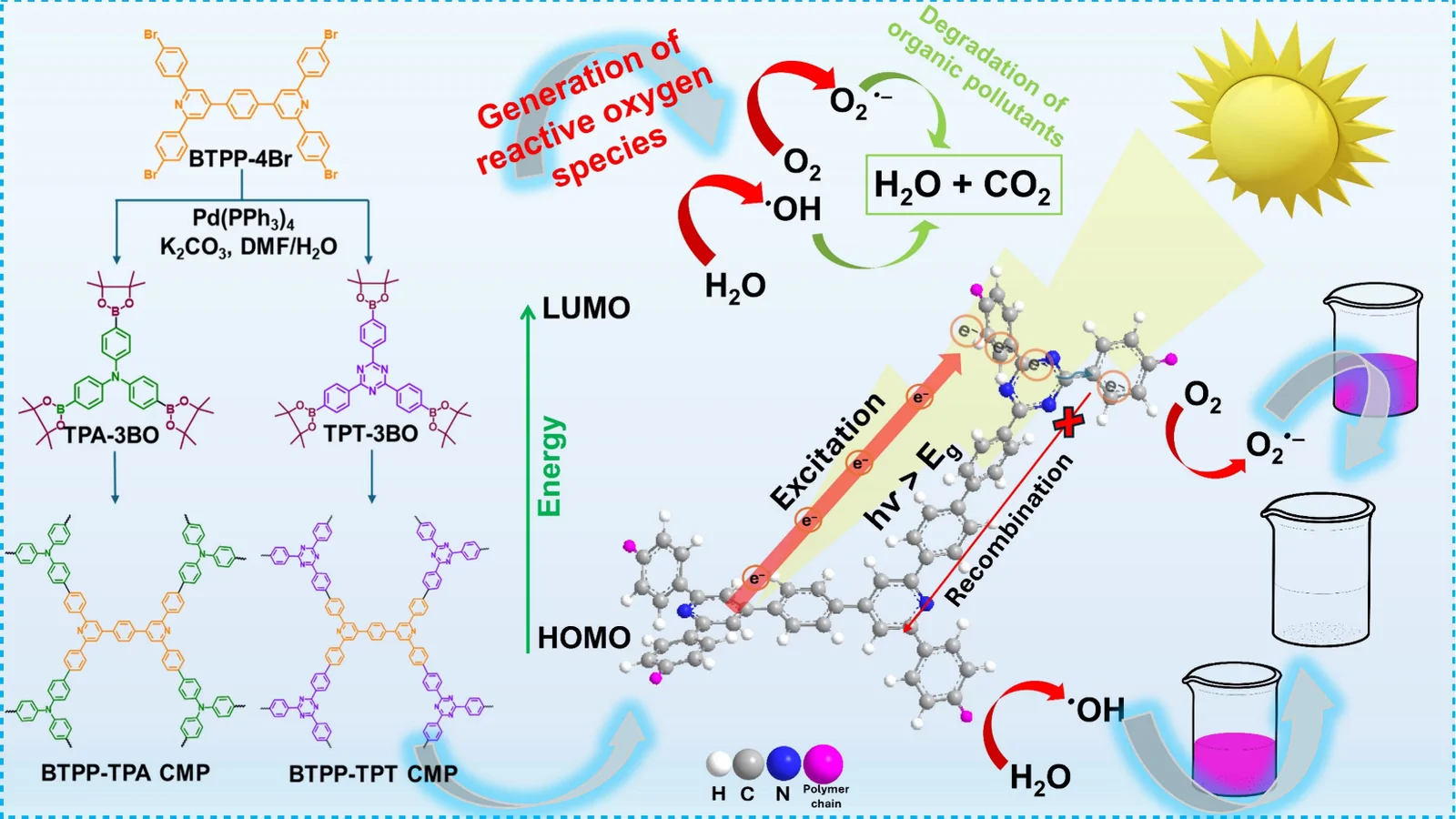
Schematic Diagram of the Synthetic Production of BTPP-Based CMPs and Their Photocatalytic Production of Reactive Oxygen Species That Oxidatively Decompose Organic Pollutants into CO_2_ and H_2_O

## 2 Experimental Section

### 2.1 Materials

All chemicals and solvents were purchased from commercial suppliers and used without further purification, unless otherwise specified. Terephthalaldehyde (98%), 4-bromoacetophenone (98%), chloroform (CHCl_3_), and methanol (MeOH) were acquired from Alfa Aesar. Ammonium acetate (NH_4_OAc) (97%) was obtained from Showa. *N*-Bromosuccinimide (NBS) (99%), 4-bromobenzonitrile (≥98%), and tetrakis­(triphenylphosphine)­palladium (Pd­(PPh_3_)_4_) (99%) were sourced from Acros. Additional materials are included Supporting Information (SI) section S1.

### 2.2 Preparation of BTPP-TPA CMP

In a Schlenk Pyrex tube, BTPP-4Br (150 mg, 0.176 mmol), TPA-3BO (146.3 mg, 0.234 mmol), Pd­(PPh_3_)_4_ (21 mg, 0.018 mmol), and potassium carbonate (244 mg, 1.77 mmol) were evacuated for 15 min. Then, a cosolvent of *N*,*N*-dimethylformamide (DMF) and water (10.34, 1.3 mL) was added. The Schlenk Pyrex device was placed in a liquid nitrogen vessel to solidify its contents, then evacuated for 10 min under vacuum. After that, the tube was put in a bath of water, and the solvent was turned into a liquid by using a dropper to rinse the bottle with water. This technique was conducted thrice. The Schlenk tube was next inserted into an oil vessel at 130 °C for 3 days. Following cooling to ambient temperature, the resulting substance was filtered and subjected to three washings with distilled water, methanol, and tetrahydrofuran (THF) until a colorless solution was obtained, then with acetone. Finally, the resultant substance was dried at 120 °C for 24 h under vacuum, yielding BTPP-TPA CMP as a pale-green powder (55.8% yield).

### 2.3 Preparation of BTPP-TPT CMP

In a Schlenk Pyrex tube, BTPP-4Br (150 mg, 0.176 mmol), TPT-3BO (161.28 mg, 0.234 mmol), Pd­(PPh_3_)_4_ (21 mg, 0.018 mmol), and potassium carbonate (244 mg, 1.77 mmol) were evacuated for 15 min. Then a cosolvent of DMF and water (10.34, 1.3 mL) was added. The Schlenk Pyrex device was placed in a liquid nitrogen vessel to solidify its contents, then evacuated for 10 min under vacuum. After that, the tube was put into a bath of water, and the solvent was turned into a liquid by using a dropper to rinse the bottle with water. This technique was conducted thrice. The Schlenk tube was next inserted into an oil vessel at 130 °C for 3 days. Following cooling to ambient temperature, the resulting substance was filtered and subjected to three washings with distilled water, methanol, and THF until a colorless solution was obtained, then with acetone. Finally, the resultant substance was dried at 120 °C under vacuum for 24 h, yielding BTPP-TPT CMP as a green powder (63.3% yield).

## 3 Results and Discussion

### 3.1 Synthesis and Characterization of CMPs

BTPP-TPA and BTPP-TPT CMPs were synthesized through a one-pot Suzuki coupling strategy using BTPP-4Br, TPA-3BO, and TPT-3BO as the corresponding building blocks (Schemes 1, S4, and S5).^20,48^ The pale-green BTPP-TPA CMP (Schemes 1 and S4) was obtained by coupling BTPP-4Br with TPA-3BO, whereas the green BTPP-TPT CMP (Schemes 1 and S5) was prepared by coupling BTPP-4Br with TPT-3BO. Both CMPs were isolated in good yields and showed poor solubility in common organic solvents, including alcohols, THF, and acetone, indicating the formation of highly cross-linked polymer networks. Fourier transform infrared (FT-IR), solid-state ^13^C cross-polarization magic-angle spinning (CP MAS) NMR spectroscopy, and X-ray photoelectron spectroscopy (XPS) were used to characterize the chemical composition of the CMPs. FT-IR spectra of BTPP-TPA and BTPP-TPT CMPs show characteristic absorption bands at 3031 and 3040 cm^–1^, corresponding to aromatic C–H stretching vibrations. The bands at 1597 and 1604 cm^–1^ are assigned to C=N stretching, while those at 1493 and 1510 cm^–1^ correspond to aromatic C=C stretching vibrations. Additional peaks at 1004.75 and 1004.27 cm^–1^ are attributed to in-plane aromatic C–H bending, whereas the bands at 820 and 812 cm^–1^ are assigned to C=C bending vibrations. The peaks at 695.71 and 695.22 cm^–1^ correspond to out-of-plane aromatic C–H bending vibrations (Figure 1a). Notably, BTPP-TPT CMP exhibited an additional signal at 1368 cm^–1^, assigned to the triazine C=N. The complete absence of C–Br and B–O stretching vibration peaks in either CMP, relative to their monomers BTPP-4Br (640 cm^–1^), TPA-3BO (1363 cm^–1^), and TPT-3BO (1349 cm^–1^), indicates successful coupling between the monomers and effective Suzuki coupling polymerization. Solid-state ^13^C NMR spectroscopy provided additional structural information, confirming complete polymerization by the absence of pinacolato carbon signals (Figure 1b). The spectra showed two primary resonance bands in both CMPs: 139.77–135.81 ppm for aromatic C=N and 127.53–127.63 ppm for aromatic C–H groups. BTPP-TPT CMP showed an additional signal at 170.32 ppm, corresponding to the triazine carbon. The elemental composition and chemical environments of carbon and nitrogen in the CMP catalysts were investigated by XPS. As shown in Figure 1c, the survey spectra of both BTPP-TPA and BTPP-TPT CMPs exhibit distinct C 1s and N 1s signals at approximately 284 and 398 eV, respectively, confirming the successful incorporation of carbon- and nitrogen-containing units into the polymer frameworks. A weak O 1s signal at around 531 eV is also observed, which can be attributed to surface-adsorbed oxygen/water species. No additional significant signals were detected in the survey spectra, suggesting the absence of obvious inorganic residues or unexpected elemental impurities in the synthesized CMPs. The high-resolution C 1s and N 1s spectra were further deconvoluted to examine the local chemical environments of carbon and nitrogen atoms (Figure S10a–d). For BTPP-TPA CMP, the high-resolution C 1s spectrum was deconvoluted into three components at 283.48, 284.87, and 285.20 eV, which are assigned to aromatic C=C/C–C/C–H, C=N, and C–N species, respectively (Figure S10a and Table S1). The corresponding N 1s spectrum exhibits two fitted peaks at 398.17 and 400.29 eV, attributable to pyridinic N=C and N–C species, respectively (Figure S10b and Table S1). For BTPP-TPT CMP, the C 1s spectrum shows two main components at 283.62 and 285.50 eV, corresponding to aromatic C=C/C–C/C–H and C=N species, respectively (Figure S10c and Table S1). The N 1s spectrum of BTPP-TPT CMP displays two peaks at 397.35 and 397.98 eV, which are assigned to pyridinic nitrogen and triazine nitrogen, respectively (Figure S10d and Table S1). These results confirm the successful incorporation of pyridine- and triazine-containing units into the corresponding CMP frameworks. Table S2 summarizes the relative peak-area contributions obtained from the high-resolution C 1s and N 1s XPS deconvolution. For BTPP-TPA CMP, the fitted C 1s components are assigned to aromatic C=C/C–C/C–H, C=N, and C–N species with relative contributions of 93.59%, 3.45%, and 2.96%, respectively. The corresponding N 1s spectrum consists of pyridinic N=C and N–C species, with contributions of 57.33% and 42.67%, respectively. For BTPP-TPT CMP, the C 1s spectrum contains aromatic C=C/C–C/C–H and C=N components with relative contributions of 90.48% and 9.52%, respectively. Its N 1s spectrum shows pyridinic nitrogen and triazine nitrogen species, accounting for 33.49% and 66.51%, respectively. It should be noted that the percentages obtained from the high-resolution C 1s and N 1s fitting represent relative peak-area contributions within each core-level spectrum, not the total elemental composition of the CMPs. The elemental compositions of BTPP-TPA and BTPP-TPT CMPs were further estimated from the XPS survey spectra by integrating the peak areas of all detected elements and applying the appropriate sensitivity-factor corrections. As summarized in Table S3, the experimentally obtained elemental ratios are in good agreement with the theoretical compositions calculated from the proposed CMP structures (Table S4), supporting the successful formation of the targeted polymer frameworks. The experimental C, N, and O contents of BTPP-TPA CMP were 88.76%, 4.49%, and 6.75%, respectively, whereas those of BTPP-TPT CMP were 85.02%, 8.28%, and 6.69%, respectively. These results indicate that BTPP-TPT CMP contains a higher nitrogen ratio than BTPP-TPA CMP, consistent with the incorporation of the nitrogen-rich triazine unit. To further verify the bulk elemental composition, elemental analysis was performed for both CMPs. BTPP-TPA CMP showed C, N, O, and H contents of 81.50%, 4.12%, 6.20%, and 4.78%, respectively, while BTPP-TPT CMP exhibited corresponding values of 77.60%, 7.56%, 6.11%, and 4.32%, respectively (Table S5). These elemental analysis results further confirm the presence of nitrogen and oxygen heteroatoms in both polymer frameworks and support the higher nitrogen content of BTPP-TPT CMP.

**Figure 1:**
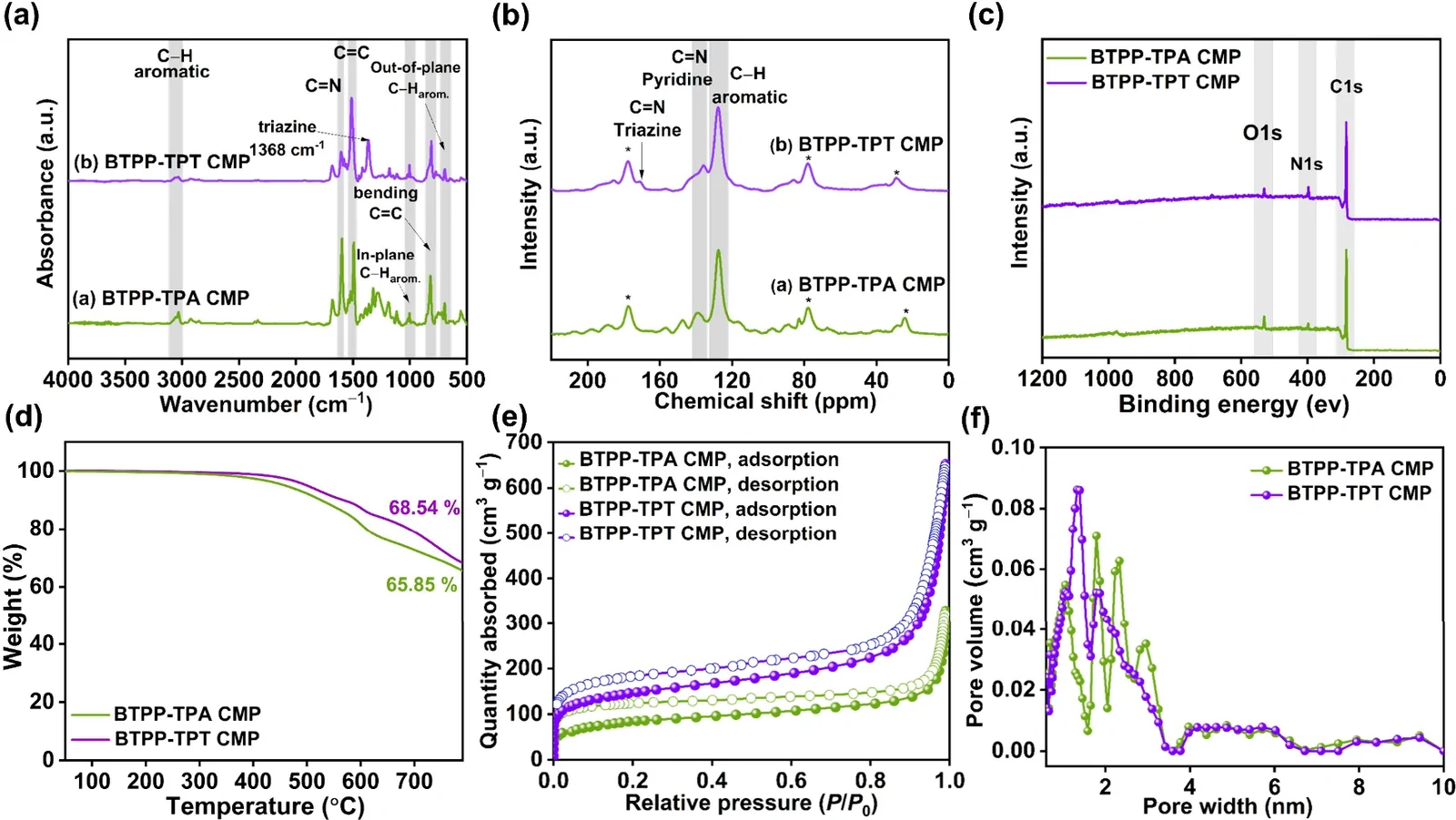
(a) FT-IR spectra, (b) solid-state ^13^C NMR measurements, (c) XPS survey spectra, (d) TGA spectra, (e) nitrogen sorption isotherms at 77 K, and (f) pore size distributions of BTPP-TPA and BTPP-TPT CMPs.

The thermal stability of both the BTPP-TPA and BTPP-TPT CMPs was assessed by thermogravimetric analysis (TGA). Decomposition temperatures (*T*_d10_) of 530.27 and 573.91 °C, with char yields of 65.85% and 68.54%, respectively, demonstrated that these CMPs exhibited exceptional thermal stability (Figure 1d and Table S6). Both CMPs exhibit exceptional thermal stability, largely due to their extensively cross-linked networks. Nonetheless, the BTPP-TPT CMP has enhanced heat resistance, attributable to its increased structural planarity, high nitrogen content, π–π stacking interactions among CMP layers, and expanded π conjugation.^9^ Prior studies have demonstrated that extended conjugation length markedly improves thermal stability by facilitating enhanced electron delocalization and increasing structural rigidity.^49–51^ The TPT unit features a 1,3,5-triazine ring, characterized by its aromaticity, higher heteroatom content, electron deficiency, and inherent rigidity, which enhances the thermal stability of the BTPP-TPT CMP.^52,53^ At 77 K, N_2_ sorption isotherms were recorded to evaluate the porosity of the synthesized CMPs (Figure 1e,f). According to the IUPAC classification, both CMPs exhibit Type IV isotherms with H4 hysteresis loops, indicating the coexistence of microporous and mesoporous features. The sharp nitrogen uptake at low relative pressure (*P*/*P*_0_ < 0.1) confirms the presence of micropores, while the increased adsorption at high relative pressure (*P*/*P*_0_ > 0.9) suggests the contribution of mesopores and interparticle voids. The Brunauer–Emmett–Teller (BET) surface areas and pore volumes are summarized in Table S7. BTPP-TPT CMP exhibited a higher BET surface area of 521 m^2^ g^–1^ and a larger pore volume of 0.99 cm^3^ g^–1^, whereas BTPP-TPA CMP showed a lower surface area of 300.16 m^2^ g^–1^ and a smaller pore volume of 0.49 cm^3^ g^–1^. The enhanced porosity of BTPP-TPT CMP can be attributed to the more planar and rigid triazine-containing TPT unit, which promotes efficient framework extension, stronger π-conjugated packing, and the formation of more accessible porous channels. In contrast, the more twisted TPA unit may reduce packing efficiency and limit the development of accessible porosity. The pore diameter distributions (PDDs), calculated using nonlocal density functional theory (NLDFT), further confirm the porous nature of both CMPs. BTPP-TPA CMP shows pore sizes in the ranges of 0.67–1.58, 1.58–2.05, 2.05–2.69, and 2.69–3.59 nm, whereas BTPP-TPT CMP displays pore-size distributions of 1.07–1.65 and 1.65–2.34 nm (Figure 1f and Table S7). The higher surface area and pore volume of BTPP-TPT CMP provide more accessible active sites and facilitate mass transport, thereby benefiting dye adsorption and photocatalytic degradation efficiency. Powder X-ray diffraction (PXRD) was applied to assess the crystallinity of the developed polymers. Figure S11 shows that the PXRD patterns of BTPP-TPA and BTPP-TPT CMPs exhibit broad diffraction features with a main broad maximum at 18.06°. The corresponding *d*-spacing is approximately 0.49 nm, which is larger than the typical π–π stacking distance; therefore, this peak is not assigned to conventional π–π interactions. Instead, the broad diffraction feature is attributed to short-range packing/order and local structural periodicity within the amorphous CMP networks.^21,54,55^ These materials typically exhibit large, low-angle diffraction peaks resulting from short-range ordering in their structures.^21,56^ The absence of sharp diffraction peaks indicates that both CMPs lack long-range crystalline order and are predominantly amorphous.

The nanoscale morphology and surface topography of the CMPs were examined by transmission electron microscopy (TEM) and high-resolution scanning electron microscopy (HR-SEM) (Figure 2). As shown in Figure 2a–c, BTPP-TPA CMP is composed of nanospheres with an average diameter of approximately 250 nm. In contrast, TEM images of BTPP-TPT CMP reveal smaller nanospheres that tend to aggregate into fibrous assemblies (Figure 2i–k). These structures have an average diameter of approximately 40 nm and extend to several micrometers in length, which may be related to the more planar TPT-3BO building block and stronger intermolecular stacking interactions.^57^ The observed morphological difference can therefore be associated with the different geometries of the monomers: the planar TPT-3BO unit promotes more ordered aggregation and fibrous network formation, whereas the more twisted TPA-3BO unit disrupts packing and favors the formation of spherical BTPP-TPA particles.^58,59^ The low-magnification TEM images (Figure 2c,k) further indicate the porous morphology of both CMPs. Consistent with the TEM results, HR-SEM images show monomer-dependent morphologies, with BTPP-TPA CMP displaying relatively uniform nanospheres and BTPP-TPT CMP showing smaller, more tightly clustered spherical/fibrous aggregates (Figure 2d,e,l,m).

**Figure 2:**
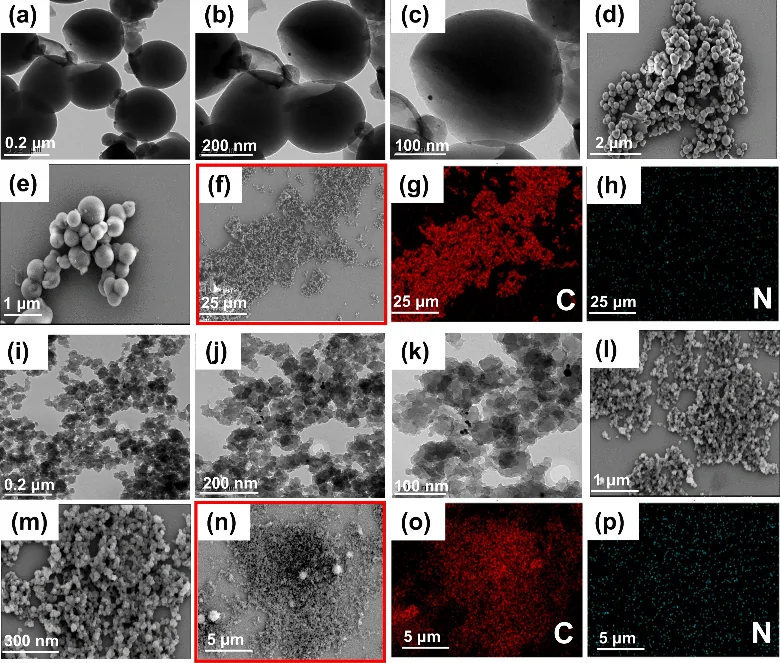
TEM images of (a–c) BTPP-TPA CMP and (i–k) BTPP-TPT CMP. SEM images of (d–f) BTPP-TPA CMP and (l–n) BTPP-TPT CMP. EDS elemental maps of (g, h) BTPP-TPA CMP and (o, p) BTPP-TPT CMP.

Energy-dispersive X-ray spectroscopy (EDS) elemental mapping confirms the presence and homogeneous distribution of carbon and nitrogen throughout both CMPs (Figure 2f–h,n–p), supporting the successful incorporation of these elements into the polymer frameworks. The aqueous stability of BTPP-TPA and BTPP-TPT CMPs was evaluated by immersing 20 mg of each CMP in water for 4 days, followed by recovery through vacuum filtration. As shown in Figure S12, the FT-IR spectra show no obvious changes after water immersion, confirming the good chemical stability of both CMPs under aqueous conditions.

### 3.2 Photophysical and Electrochemical Properties

Efficient photocatalysis requires materials with suitable optical band gaps as well as favorable photophysical and electrochemical properties. Therefore, UV–vis diffuse reflectance spectroscopy (UV–vis DRS), electrochemical impedance spectroscopy (EIS), and transient photocurrent measurements were performed to evaluate the solid-state properties of BTPP-TPA and BTPP-TPT CMPs. As shown in Figure S13, both CMPs exhibit broad light absorption extending to approximately 800 nm. The maximum absorption peaks are located at 380 nm for BTPP-TPA CMP and 373 nm for BTPP-TPT CMP, indicating their ability to absorb visible light. The slight red shift observed for BTPP-TPA CMP compared with BTPP-TPT CMP may be attributed to its more twisted molecular structure, which can facilitate intramolecular charge transfer and narrow the optical band gap.^14^ The optical band gaps (*E*_g_), estimated from the corresponding Tauc plots, are 2.66 eV for BTPP-TPA CMP and 2.97 eV for BTPP-TPT CMP (Figure 3a). Compared with the commonly used ZnO photocatalyst with a band gap of approximately 3.45 eV, both CMPs possess narrower band gaps, which are advantageous for visible-light absorption and photocatalytic dye degradation.^11^ The narrow band gap of both CMPs facilitates effective visible-light absorption and enhances the generation rate of electron–hole pairs, hence improving the degradation of organic dyes.^14^ The lowest unoccupied molecular orbital (LUMO) levels of the CMPs were estimated from cyclic voltammetry (CV), while the highest occupied molecular orbital (HOMO) levels were calculated using the optical band gaps according to HOMO = LUMO – *E*_g_ (Figure 3b,c). As summarized in Figure 3c and Table S8, BTPP-TPA CMP exhibits HOMO/LUMO levels of −6.04/–3.38 eV, whereas BTPP-TPT CMP shows corresponding values of −6.07/–3.10 eV. Similar HOMO levels of the two CMPs suggest comparable electron-donating ability, while the more negative LUMO level of BTPP-TPA CMP results in its narrower band gap. This difference may arise from the stronger electron-donating character of the TPA unit compared with the electron-deficient TPT unit.^14^ Because photocatalytic reactions depend strongly on the generation, separation, and transport of photogenerated charge carriers, transient photocurrent and EIS measurements were conducted to evaluate the charge-transfer behavior of the CMPs. In a conventional three-electrode configuration, both BTPP-TPA- and BTPP-TPT-CMP-coated electrodes exhibited clear photocurrent responses under visible-light irradiation, confirming their photoresponsive behavior (Figure 3d). The BTPP-TPA CMP- and BTPP-TPT CMP-coated electrodes exhibited minimal semicircular patterns in the high-frequency domain, indicating reduced charge-movement resistance and enhanced ionic conductivity (Figure 3e).^32^ The near-linear features in the Nyquist plots of the CMP-coated electrodes suggest minimal interfacial charge-transfer resistance, supporting efficient electron transport, which is consistent with the high surface area and good conductivity of the CMP.^60^ The spectra were fitted using an equivalent circuit containing *R*_s_, *R*_ct_, *C*, and *W*, corresponding to solution resistance, charge-transfer resistance, double-layer capacitance, and Warburg impedance, respectively (Figure S14). The *R*_s_ of the nitrogen-containing CMP-coated electrodes was obtained through the intercept of the *Z*′ axis (Figure 3e). The *R*_s_ values of BTPP-TPA and BTPP-TPT CMP electrodes are 23.06 and 18.71 Ω, respectively, while their *R*_ct_ values are 500 and 280 Ω, respectively (Figure S14 and Table S9). The lower *R*_s_ and *R*_ct_ values of BTPP-TPT CMP indicate improved electrical conductivity and more efficient interfacial charge transfer compared with BTPP-TPA CMP.^61^

**Figure 3:**
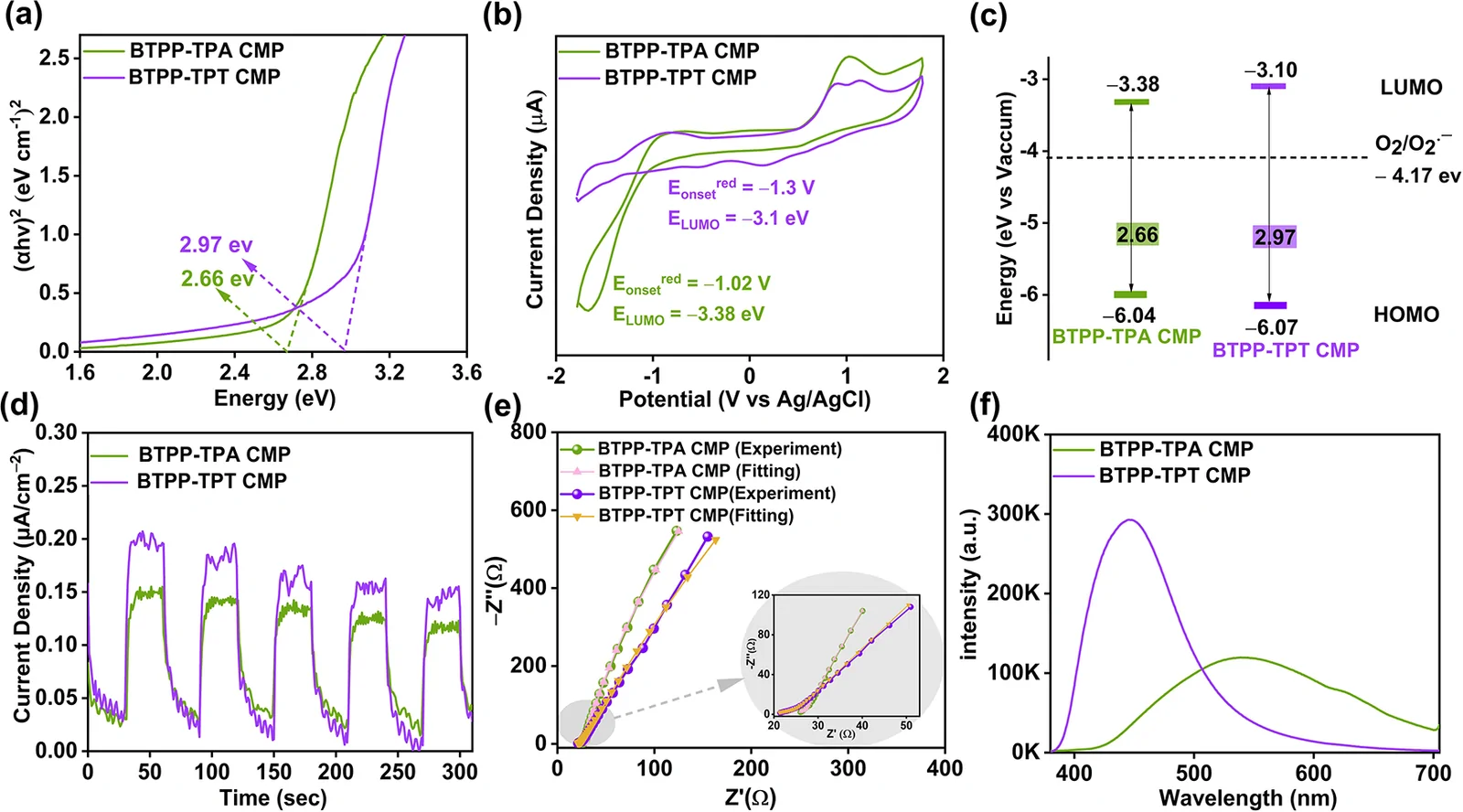
(a) Tauc graphs obtained from the UV–vis spectra, (b) cyclic voltammograms, (c) energy level charts, (d) periodic on/off photocurrent responses, (e) Nyquist graphs and their corresponding fitted graphs, and (f) photoluminescence emission spectra of BTPP-TPA and BTPP-TPT CMPs.

Photoluminescence (PL) spectroscopy was further used to evaluate the radiative recombination behavior of photogenerated charge carriers (Figure 3f).^14^ The lower PL intensity of BTPP-TPA CMP suggests reduced radiative electron–hole recombination, whereas the stronger emission of BTPP-TPT CMP indicates a higher degree of radiative recombination. Nevertheless, the superior photocatalytic performance of BTPP-TPT CMP can be mainly attributed to its higher nitrogen content, larger surface area and pore volume, stronger dye-framework interactions, and lower charge-transfer resistance, which collectively enhance adsorption-assisted photocatalytic degradation. The PL results therefore provide complementary information on charge recombination behavior, while the overall photocatalytic activity is governed by the combined effects of light absorption, porosity, dye adsorption, and interfacial charge transfer. The surface wettability of the CMPs was investigated using water contact angle measurements (Figure S15). The contact angles of BTPP-TPA and BTPP-TPT CMPs are 77.3° and 81.4°, respectively, indicating that both materials possess hydrophilic surfaces, as contact angles below 90° generally reflect good wettability.^21^ The hydrophilicity of both CMPs arises from their polar heteroatom-containing frameworks, which promote interactions between water molecules and the polymer backbone.^62^ Improved wettability can facilitate better contact between the CMP particles and the aqueous dye solution during photocatalysis, thereby enhancing particle dispersion and promoting mass transport in water. These features facilitate the removal and photocatalytic degradation of organic pollutants, such as RhB and MB. In general, hydrophilicity is positively associated with the presence of polar heteroatoms in the polymer framework. Although BTPP-TPT CMP has a higher heteroatom content, BTPP-TPA CMP exhibits a slightly lower water contact angle, indicating better surface wettability.^32^ This difference may be attributed to the more planar structure of the TPT unit, which promotes stronger π–π stacking and may partially shield the heteroatom sites from direct interaction with water molecules.^21,63^ In contrast, the more twisted TPA unit can reduce π–π stacking and expose more polar surface sites, thereby enhancing wettability.

To substantiate the D–A characteristics of the BTPP-CMPs, we performed density functional theory calculations at the B3LYP/6-31G­(d,p) level to elucidate the structure–property relationships of the BTPP-TPA and BTPP-TPT frameworks. As depicted in Figure 4a, the BTPP-TPA CMP exhibits a clear D–A configuration in which the TPA unit operates as the electron-donating component, reflected by the predominant localization of the HOMO electron density on TPA, while the BTPP moiety acts as the electron acceptor, as indicated by the LUMO density centered on this fragment. In contrast, the BTPP-TPT CMP displays an inverted electronic arrangement (Figure 4b), wherein the BTPP unit assumes the electron-donating role, with the HOMO concentrated on BTPP, whereas the TPT segment functions as the electron acceptor, consistent with the LUMO electron density distributed primarily over the TPT unit. Collectively, the results substantiate that both synthesized CMPs embody a defined D–A framework, which intrinsically promotes enhanced inter- and intramolecular charge-transport processes. To further support the relationship between molecular structure and photocatalytic performance, electrostatic potential (ESP) calculations were performed to compare the electronic environments of BTPP-TPA and BTPP-TPT CMPs. The red regions, corresponding to negative ESP, indicate areas of high electron density, primarily associated with electronegative nitrogen atoms. In contrast, the blue regions represent positive ESP and are mainly located around hydrogen atoms and electron-deficient regions of the aromatic skeletons. The ESP distributions reveal that electron-rich regions are mainly localized around the nitrogen-containing sites, including pyridine, C=N, C–N, and triazine moieties. BTPP-TPT CMP displays a more pronounced negative potential around the triazine regions, indicating a higher electron-density distribution than that of BTPP-TPA CMP (Figure 4). These electron-rich nitrogen sites can enhance interactions with cationic RhB and MB molecules via electrostatic and dipole–dipole interactions, thereby promoting dye enrichment near the photocatalytic surface. This interpretation is consistent with the adsorption results, which show that BTPP-TPT CMP exhibits stronger dye uptake, especially toward RhB.

**Figure 4:**
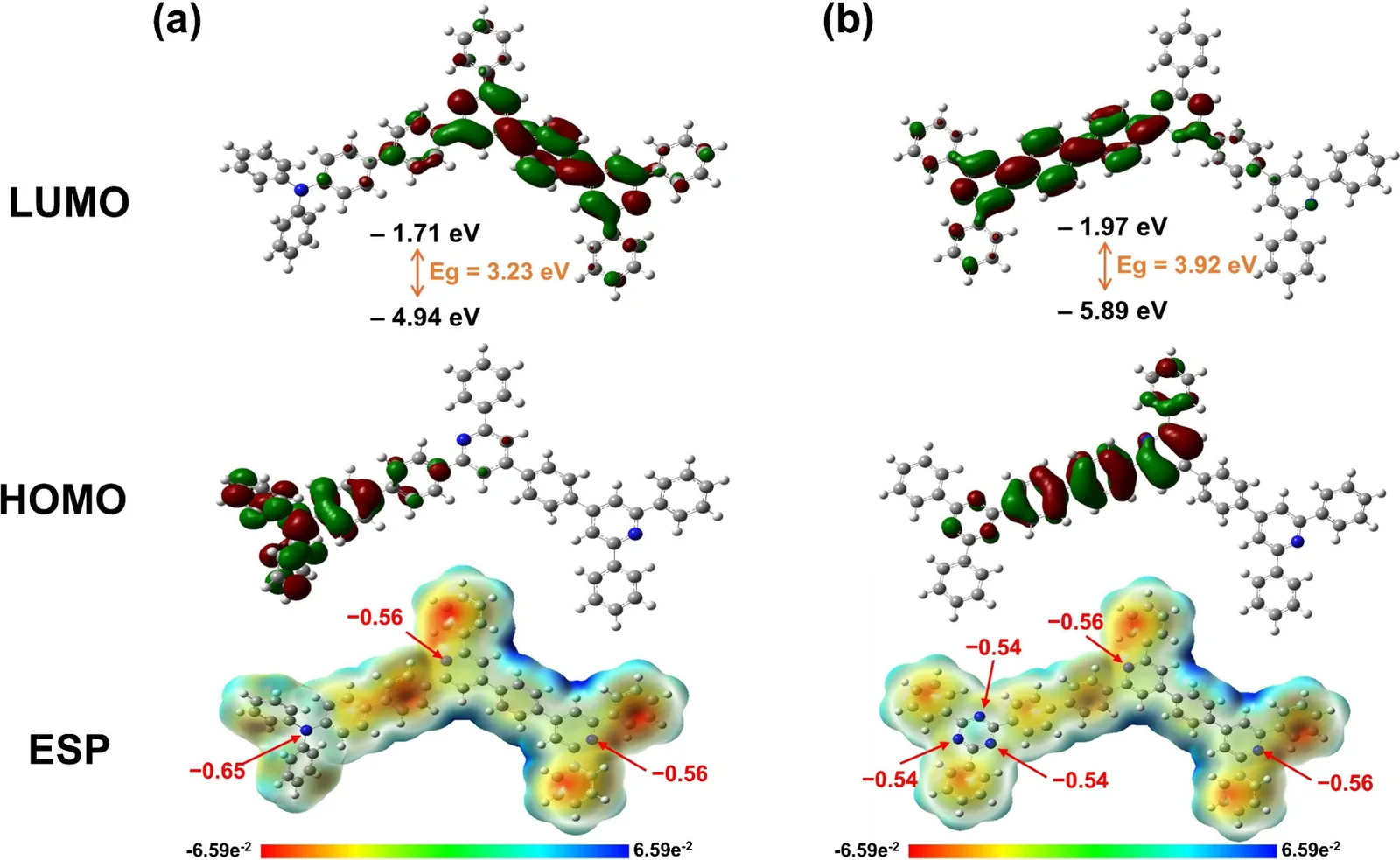
HOMO and LUMO distributions and ESP distributions of (a) BTPP-TPA and (b) BTPP-TPT CMPs. Red indicates negative potential, and positive regions are indicated in blue.

### 3.3 Performance of Dye Adsorption

Our synthesized BTPP-based CMPs possess several favorable features for dye adsorption, including high specific surface areas, hydrophilic surfaces, extended π-conjugated frameworks, and nitrogen-containing interaction sites. These characteristics facilitate their interaction with ionic dyes such as RhB and MB in aqueous media.^11^ In particular, the nitrogen atoms within the CMP frameworks can provide electron-rich sites that enhance dye-framework interactions through electrostatic interactions, dipole–dipole interactions, hydrogen bonding, and π–π interactions.^54,64^ Therefore, BTPP-TPA and BTPP-TPT CMPs were evaluated as adsorbents for the removal of the cationic dyes RhB and MB from aqueous solutions. The adsorption performance was monitored by UV–vis spectroscopy at different time intervals after adding 6 mg of each CMP to the dye solution. Changes in the characteristic absorption peaks of RhB and MB were used to evaluate the adsorption process (SI section S16). As shown in Figures 5 and S16, both CMPs exhibited clear adsorption ability toward RhB and MB. For RhB adsorption, BTPP-TPA CMP reached an adsorption efficiency of 51.28% after 30 min, whereas BTPP-TPT CMP exhibited a much higher efficiency of 92.26% under the same conditions (Figures 5a,b,e, and S16a). After extending the adsorption time to 90 min, the removal efficiencies increased to 76.75% for BTPP-TPA CMP and 98.29% for BTPP-TPT CMP, indicating faster adsorption kinetics and a stronger RhB affinity for BTPP-TPT CMP. For MB adsorption, both CMPs exhibited rapid, high removal efficiencies. After 30 min, BTPP-TPA and BTPP-TPT CMPs achieved adsorption efficiencies of 97.94% and 88.99%, respectively (Figures 5c,d,f, and S16b). After 90 min, the efficiencies further increased to 99.26% and 98.24%, respectively, confirming the excellent MB adsorption ability of both CMPs. The adsorption behavior of these CMPs is influenced by several factors, including BET surface area, pore volume, nitrogen content, molecular planarity, surface polarity, and secondary interactions with dye molecules. Notably, BTPP-TPT CMP exhibits a higher BET surface area of 521 m^2^ g^–1^ and a larger pore volume of 0.99 cm^3^ g^–1^ compared with BTPP-TPA CMP, which has a surface area of 300.16 m^2^ g^–1^ and a pore volume of 0.49 cm^3^ g^–1^. These textural advantages provide more accessible adsorption sites and facilitate dye diffusion within the porous framework. In addition, the more planar TPT unit and higher nitrogen content of BTPP-TPT CMP can enhance surface polarity, electron-density distribution, and dye-framework affinity. The stronger adsorption of RhB by BTPP-TPT CMP can therefore be attributed to the combined effects of its larger accessible surface area, higher pore volume, nitrogen-rich framework, and higher electron density, and stronger π–π/electrostatic interactions with the aromatic cationic dye molecules.^11,12,65^ The interaction between the CMPs and dye molecules was further supported by FT-IR analysis before and after adsorption. After RhB adsorption, the characteristic aromatic C=C stretching vibrations of the CMPs shifted slightly from 1596 and 1602 cm^–1^ to 1600 and 1605 cm^–1^ for BTPP-TPA and BTPP-TPT CMPs, respectively. In addition, the C=O stretching band of RhB shifted from 1706 cm^–1^ to 1682 and 1684 cm^–1^ after adsorption onto BTPP-TPA and BTPP-TPT CMPs, respectively (Figures S17 and S18). Similar changes were observed after MB adsorption, where the C=C stretching bands of the CMPs shifted to 1604 and 1605 cm^–1^ for BTPP-TPA and BTPP-TPT CMPs, respectively (Figures S19 and S20). These spectral changes confirm the presence of dye-framework interactions, mainly involving π–π stacking, hydrogen bonding, electrostatic interactions, and dipole–dipole interactions. Overall, the adsorption results demonstrate that both CMPs can effectively capture RhB and MB from aqueous solutions, while the superior RhB adsorption of BTPP-TPT CMP is closely related to its higher surface area, larger pore volume, nitrogen-rich structure, and stronger dye-framework interactions.

**Figure 5:**
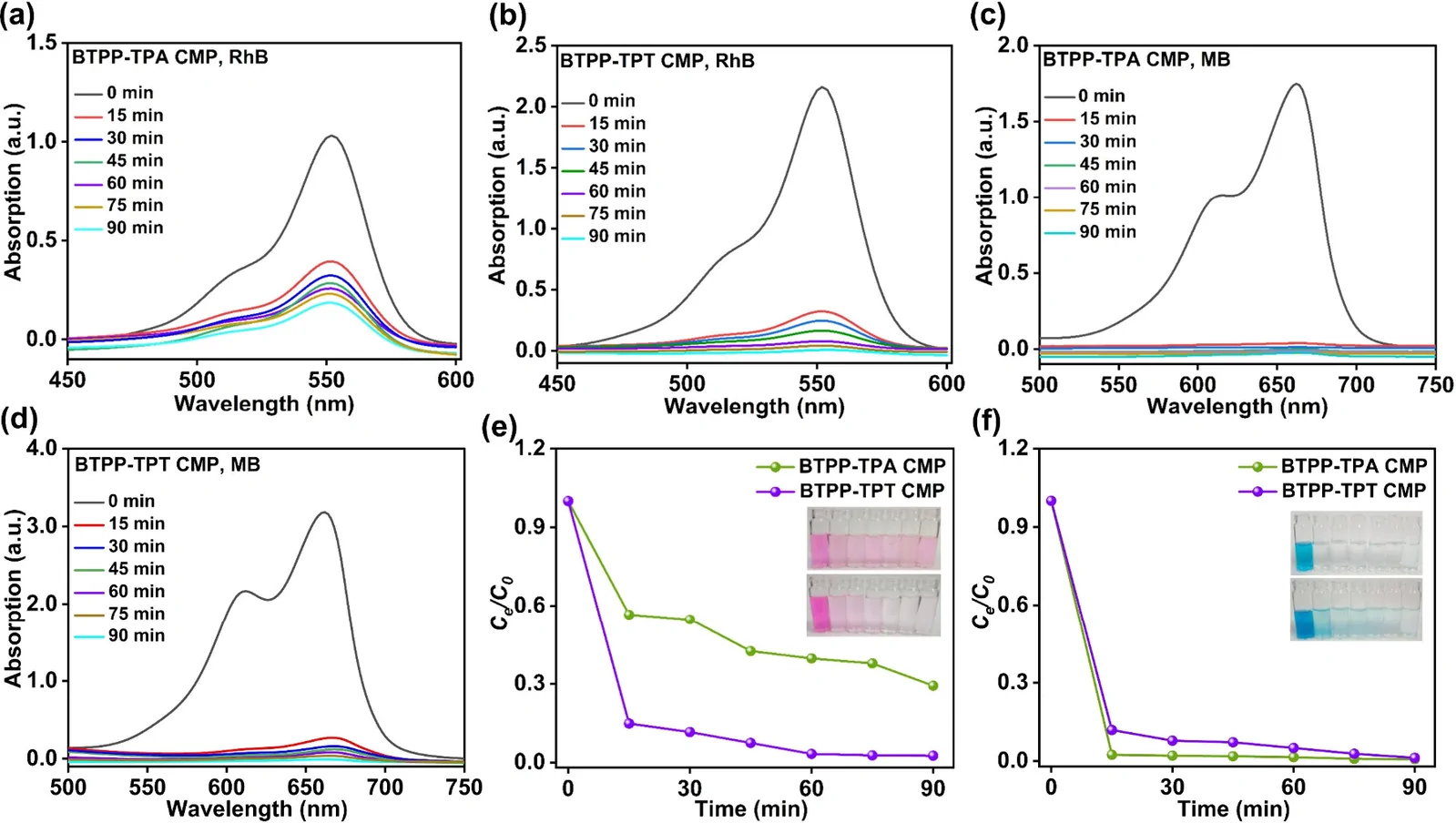
(a–d) UV–vis spectra of (a, b) RhB and (c, d) MB dye solutions at different time intervals after the addition of the (a, c) BTPP-TPA and (b, d) BTPP-TPT CMPs. (e, f) Adsorption rates of (e) RhB and (f) MB organic dyes from an aqueous solution via CMPs.

### 3.4 Performance of Dye Photodegradation

Given their high surface area, conjugated frameworks, suitable porosity, favorable photophysical properties, and appropriate band gaps, we examined BTPP-TPA and BTPP-TPT CMPs as visible-light photocatalysts for degrading aqueous organic pollutants, focusing on the cationic dyes RhB and MB under irradiation above 450 nm. The photocatalytic performance of the CMP (6 mg) was evaluated using RhB and MB (20 mg L^–1^, 60 mL) in an aqueous solution. The photocatalytic degradation experiments were conducted under ambient air conditions without N_2_ or Ar purging; therefore, dissolved O_2_ in the air-saturated aqueous solution served as the main oxygen source for ROS generation. To establish adsorption–desorption equilibrium, the suspensions were stirred in the dark for 60 min prior to illumination. Because BTPP-based CMPs exhibit clear adsorption ability toward RhB and MB, the photocatalytic process should be regarded as an adsorption-assisted degradation pathway. The dye concentration measured after this dark-equilibrium step was used as *C*_0_ for calculating the apparent photodegradation efficiency and rate constant. Therefore, the reported photocatalytic performance reflects the decrease in dye concentration during the irradiation stage rather than the initial adsorption contribution. Nevertheless, adsorbed dye molecules on the CMP surface may also participate directly in the photocatalytic reaction. Under visible-light irradiation, photogenerated electrons and holes are produced within the CMP framework and react with dissolved O_2_ and H_2_O/OH^–^ to generate reactive oxygen species. Thus, RhB and MB degradation likely occurs mainly at or near the CMP–solution interface, where adsorbed dye molecules are oxidized by surface-generated ROS, while diffused ROS may also degrade free dye molecules in solution. This adsorption-assisted interfacial mechanism explains the enhanced performance of BTPP-TPT CMP, which combines strong dye enrichment, efficient charge separation/transfer, and ROS generation. After 90 min of visible-light irradiation, RhB was efficiently degraded, with removal efficiencies of 96.69% for BTPP-TPA CMP and 99.60% for BTPP-TPT CMP (Figures 6a and S21a,b). For MB degradation, BTPP-TPA CMP showed a much lower degradation efficiency of 7.94%, whereas BTPP-TPT CMP achieved 82.52% removal after 90 min of visible-light irradiation (Figures 6b and S21c,d). Nearly complete RhB degradation was achieved by both CMPs, with BTPP-TPA CMP reaching 99.08% removal after 120 min and BTPP-TPT CMP reaching 99.31% removal after only 60 min (Figure 6c). In addition, BTPP-TPT CMP effectively degraded MB after 180 min, achieving a photodegradation efficiency of 95.8% (Figure 6d). These results demonstrate the superior photocatalytic activity of BTPP-TPT CMP toward the degradation of cationic RhB and MB dyes. Zeta-potential measurements revealed negatively charged surfaces for both CMPs, with values of −70.4 mV for BTPP-TPT CMP and −12.6 mV for BTPP-TPA CMP. The negatively charged surface, together with electron-rich N=C and N–C sites, can strengthen the interaction with cationic RhB and MB molecules, thereby promoting dye adsorption near the photocatalytic surface. In addition, the D–A framework can facilitate photoinduced charge separation by promoting exciton dissociation and reducing electron–hole recombination.^31^ These combined effects contribute to the enhanced adsorption-assisted photocatalytic degradation performance of BTPP-TPT CMP. The superior photocatalytic performance of BTPP-TPT CMP can be attributed to the synergistic effects of its higher nitrogen content, enhanced porosity, more planar framework, and improved charge-transfer properties. Elemental analysis and XPS confirm that BTPP-TPT CMP contains a higher nitrogen content than BTPP-TPA CMP due to the incorporation of triazine units. These nitrogen-rich sites create electron-rich/negatively polarized regions, as supported by ESP calculations, which can strengthen interactions with cationic RhB and MB molecules through electrostatic and dipole–dipole interactions. In addition, the N_2_ sorption results show that BTPP-TPT CMP possesses a higher BET surface area of 521 m^2^ g^–1^ and a larger pore volume of 0.99 cm^3^ g^–1^ compared with BTPP-TPA CMP, which exhibits a surface area of 300.16 m^2^ g^–1^ and a pore volume of 0.49 cm^3^ g^–1^. This higher porosity provides more accessible adsorption and photocatalytic sites and facilitates mass transport during dye degradation. Moreover, the planar TPT unit promotes stronger π conjugation, better framework extension, and more efficient charge delocalization. This is consistent with the EIS results, which show that BTPP-TPT CMP exhibits lower charge-transfer resistance than BTPP-TPA CMP, indicating more efficient interfacial charge transfer. Therefore, the enhanced activity of BTPP-TPT CMP is not solely related to its nitrogen content, but results from the combined contributions of nitrogen-rich interaction sites, higher surface area, larger pore volume, improved planarity, stronger dye-framework affinity, and more efficient charge separation/transport. Our CMP photocatalysts exhibited RhB and MB degradation performances that are comparable to or better than those of many reported photocatalysts, including metal sulfides, metal oxides, COFs, hyper-cross-linked polymers, and conjugated polymers, under similar photocatalytic conditions (Tables S10 and S11).

**Figure 6:**
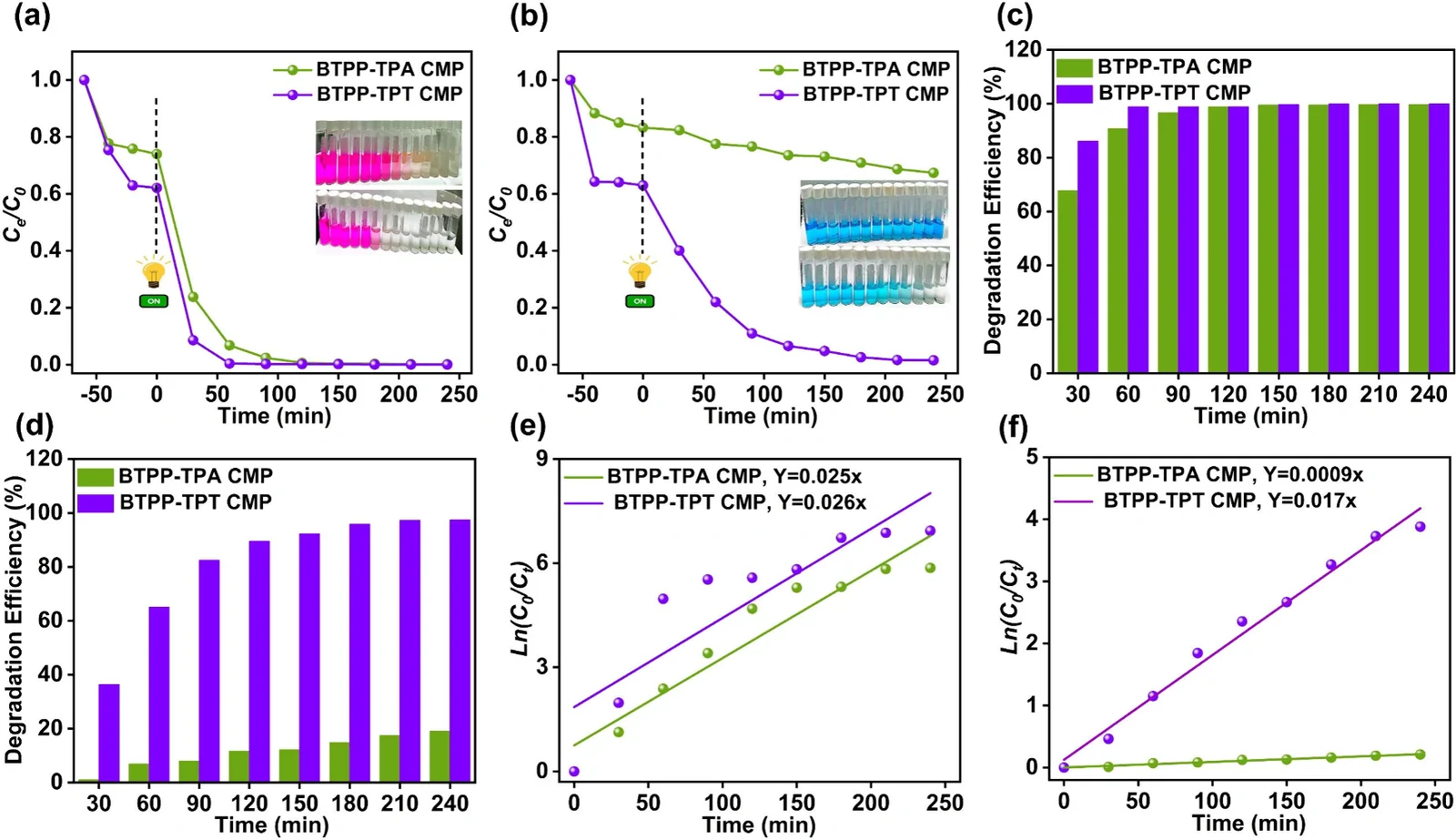
(a, b) Photodegradation rates of an aqueous solution of (a) RhB (20 mg L^–1^, pH = 3) and (b) MB (20 mg L^–1^, pH = 3) using BTPP-TPA (6 mg) and BTPP-TPT (6 mg) CMPs, evaluated across various time intervals. (c, d) Photodegradation performance of BTPP-TPA and BTPP-TPT CMPs for the degradation of (c) RhB and (d) MB in water under visible-light irradiation for different time intervals. (e, f) Pseudo-first-order kinetic plots for the photodegradation of (e) RhB and (f) MB dyes.

The photocatalytic performance of organic and inorganic semiconductors is generally governed by the efficiency of charge-carrier generation, separation, and transfer. Several strategies have been developed to address fast electron–hole recombination, including heterojunction construction, cocatalyst incorporation, and molecular π conjugation engineering. Heterojunctions can create interfacial electric fields that spatially separate photogenerated electrons and holes, while cocatalysts can accelerate interfacial charge transfer and provide additional active sites for redox reactions. In contrast to these composite-based approaches, the present BTPP-based CMPs rely on molecular-level donor–acceptor and π-conjugation engineering. The D–A framework facilitates photoinduced intramolecular charge transfer, while the extended π-conjugated backbone reduces exciton recombination and improves charge mobility.^66^ In addition, nitrogen-rich heteroatomic sites introduce electron-rich regions that enhance interactions with cationic RhB and MB molecules through electrostatic and dipole–dipole interactions.^9^ The extension of π conjugation further reduces exciton recombination, increases charge separation, and narrows the band gap between the HOMO and LUMO levels.^67^ These modifications result in tunable electronic properties and asymmetric electronic structures that significantly enhance charge carrier separation and transport, positioning D–A-based CMPs as highly promising materials for optoelectronic and visible light-driven photocatalytic applications. Additionally, the degradation efficiency of BTPP-based CMPs exceeds that of other reported pyridine-based materials, further highlighting their effectiveness in photocatalytic systems (Table S12). To ensure economical application and minimize secondary contamination, BTPP-TPT CMP was recycled for the photodegradation of RhB and MB over three cycles under similar conditions. The BTPP-TPT CMP exhibited significant activity, with a minimal 0.24% and 3.74% decline in RhB and MB degradation, respectively (Figure S22a,b). The recovered catalyst was further analyzed by FT-IR spectroscopy, revealing no obvious peak shifts or changes in intensity after cycling (Figure S23), confirming the remarkable stability of the CMP photocatalysts.^38^ Several characterization techniques, including FT-IR, XRD, and HR-SEM, were used to evaluate the stability of the BTPP-TPT CMP following RhB photodegradation, to assess its stability and practical efficacy. The FT-IR analysis of the recovered catalyst showed no notable shifts or changes in peak intensity after photodegradation, thereby affirming the remarkable stability of the BTPP-TPT CMP photocatalysts, even after RhB photodegradation under varying pH conditions (Figure S24). XRD and SEM measurements were conducted on the BTPP-TPT CMP following RhB photodegradation at pH 3. The PXRD patterns exhibited no significant variations in peak intensity or position, signifying negligible structural modification. Furthermore, SEM pictures demonstrated that the BTPP-TPT CMP preserved its distinctive nanosphere morphology, despite prolonged photodegradation (Figure S25a–e).

To analyze the photodegradation kinetics of RhB and MB, the irradiation-stage data were fitted using the Langmuir–Hinshelwood pseudo-first-order model: ln­(*C*_0_/*C*_*t*_) = *kt*, where *C*_0_ is the dye concentration after the dark adsorption–desorption equilibrium step, *C*_*t*_ is the dye concentration after irradiation for time *t*, and *k* is the apparent pseudo-first-order rate constant. The linear relationship between ln­(*C*_0_/*C*_*t*_) and irradiation time indicates that the photocatalytic degradation of RhB and MB follows apparent pseudo-first-order kinetics. For RhB degradation, BTPP-TPT CMP exhibited a slightly higher apparent rate constant of 2.6 × 10^–2^ min^–1^ compared with BTPP-TPA CMP, which showed a value of 2.5 × 10^–2^ min^–1^. In the case of MB degradation, BTPP-TPT CMP displayed a much higher rate constant of 1.7 × 10^–2^ min^–1^, greatly exceeding that of BTPP-TPA CMP (9.0 × 10^–4^ min^–1^). These results further confirm the enhanced photocatalytic activity of BTPP-TPT CMP, particularly for MB degradation (Figure 6e and 6f).

Based on the above results, BTPP-TPT CMP was identified as the most efficient photocatalyst for the degradation of RhB and MB under visible-light irradiation (>450 nm). Therefore, BTPP-TPT CMP was selected to investigate the effects of solution pH and catalyst dosage on photocatalytic degradation performance. The pH of the dye solution is an important factor because it influences the ionization state of dye molecules and the electrostatic interactions between the dye and the photocatalyst surface.^9^ The pH-dependent photodegradation performance was evaluated using 60 mL aqueous solutions of RhB or MB (20 mg L^–1^) containing 6 mg of BTPP-TPT CMP over a pH range of 3.0–9.0 (Figure 7a–d). The pH values were adjusted using appropriate amounts of NaOH or HCl solution. For RhB degradation, BTPP-TPT CMP showed the highest activity at pH 3.0, achieving 99.31% removal within 60 min (Figures 7a and S26–S29). The degradation rate decreased gradually as the pH increased, with apparent rate constants of 2.6 × 10^–2^, 2.1 × 10^–2^, 1.8 × 10^–2^, and 1.4 × 10^–2^ min^–1^ at pH 3.0, 5.0, 7.0, and 9.0, respectively (Figure 7b). The enhanced RhB degradation under acidic conditions can be attributed mainly to the cationic form of RhB, which promotes stronger electrostatic attraction with the negatively charged/electron-rich regions of the BTPP-TPT CMP surface. In addition, the presence of chloride ions introduced during pH adjustment with HCl may contribute to the formation of reactive chlorine species through reactions with photogenerated holes, which could further assist the degradation process. Figure S30 shows that RhB can exist in cationic (RhBH^+^) and zwitterionic (RhB^±^) forms depending on the solution pH. At pH 1.0–3.0, RhBH^+^ is dominant, favoring electrostatic interaction with BTPP-TPT CMP. At pH values above 4.0, RhB predominantly exists in its zwitterionic form, which weakens electrostatic attraction and reduces degradation efficiency.^31^ For MB degradation, BTPP-TPT CMP also exhibited strong photocatalytic activity under acidic to neutral conditions, achieving removal efficiencies of 95.80%, 98.56%, and 90.51% at pH 3.0, 5.0, and 7.0, respectively, after 180 min (Figures 7c and S31–S34). The corresponding rate constants were 1.7 × 10^–2^, 1.6 × 10^–2^, 1.5 × 10^–2^, and 0.8 × 10^–2^ min^–1^ at pH 3.0, 5.0, 7.0, and 9.0, respectively (Figure 7d), indicating that MB degradation is less efficient under alkaline conditions. As shown in Figure S35, MB can exist in different protonation states depending on the solution pH.^68^ Positively charged MB species within the acidic-to-neutral pH range can interact more effectively with the negatively charged BTPP-TPT CMP surface, whereas reduced electrostatic attraction at higher pH values leads to lower degradation efficiency. Under strongly acidic conditions (pH < 2.6), the triprotonated species MBH_3_^2+^ is dominant, in which the terminal amine groups are protonated. In the pH range of 2.6–8.33, MB mainly exists as mono- or diprotonated species that retain a positive charge and can interact strongly with the negatively charged BTPP-TPT CMP surface via electrostatic attraction. When the pH exceeds 8.33, MB is converted to the negatively charged form (MB^–^), weakening its electrostatic interaction with the negatively charged CMP surface and decreasing degradation efficiency. Therefore, considering both the high removal efficiency and faster degradation kinetics, pH 3.0 was selected as a suitable operating condition for the photocatalytic degradation of both RhB and MB using BTPP-TPT CMP.

**Figure 7:**
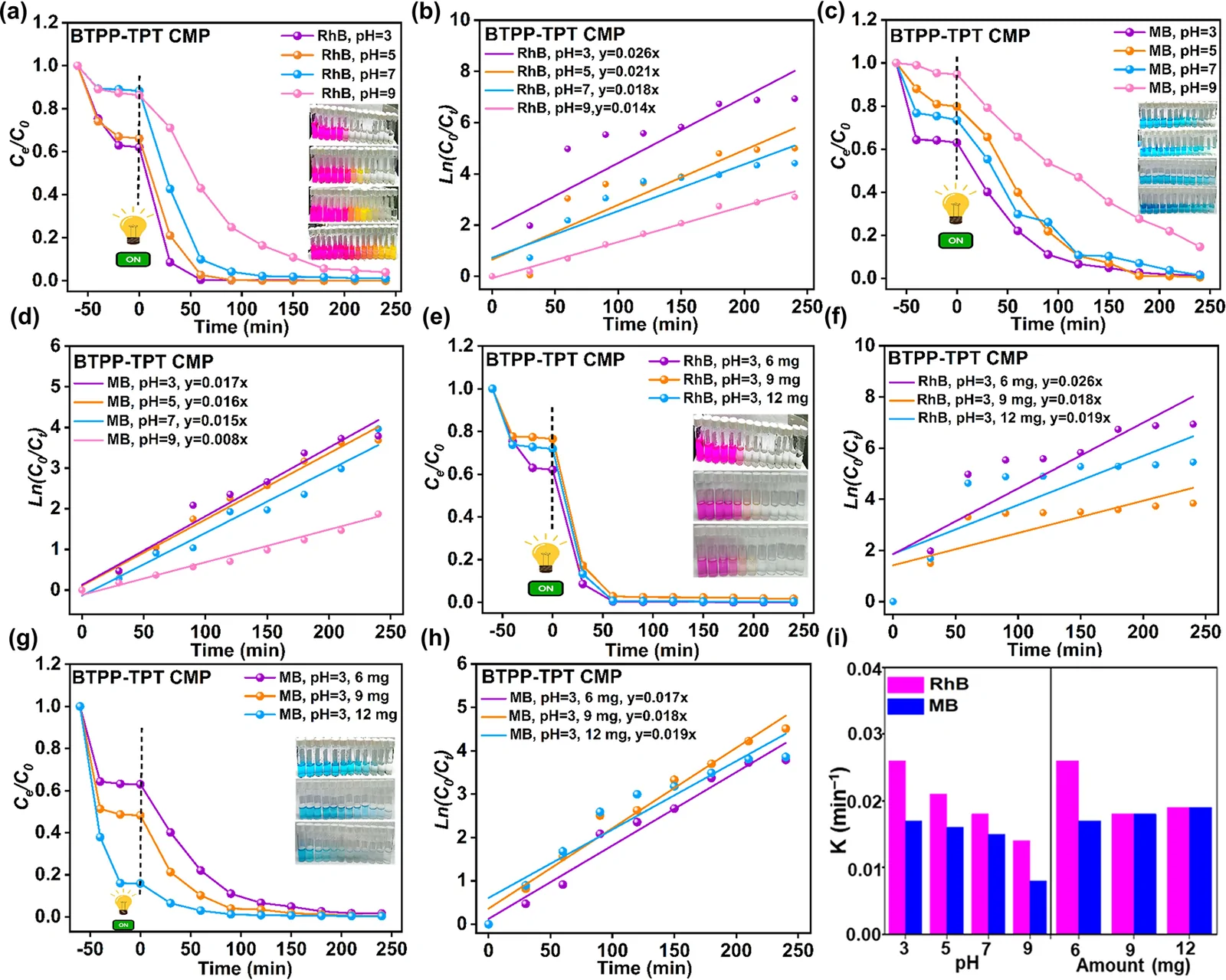
(a–h) Photodegradation rates of aqueous solutions of the cationic RhB and MB dyes using BTPP-TPT CMP, along with their respective pseudo-first-order kinetic plots under varying experimental conditions: (a, b) different levels of pH (RhB: 20 mg L^–1^; CMP dosage: 6 mg); (c, d) different levels of pH (MB: 20 mg L^–1^; CMP dosage: 6 mg); (e, f) different dosages of CMP (RhB: 20 mg L^–1^; pH: 3); (g, h) different dosages of CMP (MB: 20 mg L^–1^; pH: 3). (i) Calculated reaction rate constant *k* of aqueous solutions of the cationic RhB and MB dyes onto BTPP-TPT CMP under different conditions.

The effect of catalyst dosage was then examined because photocatalytic efficiency is strongly influenced by the amount of accessible photoactive material. At pH 3.0, RhB and MB solutions (20 mg L^–1^, 60 mL) were treated with different amounts of BTPP-TPT CMP ranging from 6.0 to 12.0 mg (Figure 7e–h). Increasing the catalyst dosage from 6.0 to 12.0 mg did not significantly improve the degradation rate for either dye (Figures 7e,g, S26, S31, and S36–S39). For RhB, the apparent rate constants obtained using 6.0, 9.0, and 12.0 mg of catalyst were 2.6 × 10^–2^, 1.8 × 10^–2^, and 1.9 × 10^–2^ min^–1^, respectively (Figure 7f). For MB, the corresponding rate constants were 1.7 × 10^–2^, 1.8 × 10^–2^, and 1.9 × 10^–2^ min^–1^, respectively (Figure 7h). The absence of a substantial improvement at higher catalyst loadings may be attributed to catalyst aggregation, increased light scattering, and reduced light penetration, which limit the number of effectively illuminated active sites.^69,70^ Therefore, 6.0 mg of BTPP-TPT CMP at pH 3.0 was selected as the optimized condition for subsequent RhB and MB photodegradation experiments (Figure 7i).

### 3.5 Mechanism and Pathway of Photodegradation

The mechanism of photocatalytic dye degradation was investigated by radical-trapping experiments using BTPP-TPT CMP as a representative photocatalyst. To identify the main reactive species involved in the degradation of RhB and MB under visible-light irradiation, *p*-benzoquinone (BQ), isopropanol (IPA), and EDTA-2Na were used as scavengers for superoxide radicals (O_2_^•–^), hydroxyl radicals (^•^OH), and photogenerated holes (h^+^), respectively (Figures S40–S42). For RhB degradation (Figure S40), the addition of BQ caused the most significant decrease in degradation efficiency, indicating that O_2_^•–^ is the dominant reactive species. IPA also reduced the degradation efficiency, confirming the participation of ^•^OH radicals, whereas EDTA-2Na caused only a minor change, suggesting that direct oxidation by h^+^ plays a limited role. For MB degradation (Figure S41), the degradation efficiency was strongly suppressed by both BQ and IPA, indicating that O_2_^•–^ and ^•^OH jointly dominate the MB degradation pathway. In contrast, EDTA-2Na showed a much weaker inhibitory effect, further confirming that photogenerated holes are not the main active species. These results demonstrate that the photocatalytic degradation of RhB and MB over BTPP-TPT CMP proceeds mainly through ROS-mediated oxidation, with O_2_^•–^ and ^•^OH as the principal reactive species. Under visible-light irradiation, photogenerated electrons in the CMP framework react with dissolved O_2_ to generate O_2_^•–^, while photogenerated holes can oxidize H_2_O/OH^–^ to form ^•^OH. The generated ROS then attack and degrade dye molecules adsorbed on or near the CMP surface, as illustrated in Figure S42.

The visible-light photocatalytic degradation pathways of both dyes over the BTPP-TPT CMP were elucidated by identifying the reaction intermediates using LC-MS (Figures S43 and S44). In the case of RhB, UV–vis spectral monitoring revealed a gradual hypsochromic shift of the characteristic absorption band from 555 nm to shorter wavelengths as irradiation time increased (Figures S26 and S27), indicating a stepwise N-deethylation process. After 30 min of illumination, the main absorption band shifted to approximately 535–537 nm, consistent with the formation of *N*,*N*,*N*′-triethylrhodamine (λ_max_ ≈ 539 nm).^31^ With prolonged irradiation (60 min), additional bands appeared at around 508 and 497 nm, corresponding to *N*-ethylrhodamine and further deethylated rhodamine species, respectively. The corresponding mass signals at *m*/*z* 415, 387, and 359 further corroborate the stepwise deethylation pathway (Scheme S6). Subsequent deamination and decarboxylation produced intermediates at *m*/*z* 331, 303, and 287, followed by chromophore cleavage and ring-opening reactions yielding lower-mass fragments (*m*/*z* 261, 199, and 117). These intermediates were ultimately mineralized to CO_2_ and H_2_O (Schemes S6 and S7 and Figure S43). For MB, a slight blue shift of the main absorption band (λ_max_ = 665 nm) was observed with increasing irradiation time during photocatalysis (Figure S31). LC-MS analysis revealed a stepwise N-demethylation process, yielding demethylated intermediates at *m*/*z* 270, 256, 242, and 228 (Scheme S8). Continued oxidative degradation led to chromophore cleavage, producing fragments with *m*/*z* values of 303, 231, 215, 201, 185, 173, 163, 158, 149, 147, 142, 136, and 117, followed by complete mineralization to CO_2_ and H_2_O (Schemes S8 and S9 and Figure S44).

To clarify whether the individual building blocks contribute to dye removal, control experiments were performed using BTPP-4Br, TPA-3BO, and TPT-3BO under the same conditions used for the CMP photocatalysts. As shown in Figures S45 and S46, the individual building blocks exhibited much lower photocatalytic degradation efficiencies toward RhB and MB than the corresponding BTPP-TPA and BTPP-TPT CMPs. This result indicates that the enhanced photocatalytic performance does not originate from isolated monomer units alone, but rather from the formation of extended, porous, conjugated polymer networks. The superior activity of the CMPs can be attributed to their higher accessible surface area, extended π conjugation, improved light absorption, and more efficient charge-carrier separation and transport within the cross-linked donor–acceptor framework. In contrast, BTPP-4Br, TPA-3BO, and TPT-3BO are discrete, nonporous molecular building blocks that lack continuous conjugated pathways and interconnected pore channels, resulting in limited dye interaction and inefficient photoinduced charge transfer. Therefore, these control experiments confirm that polymer network formation plays a critical role in promoting dye adsorption, ROS generation, and the photocatalytic degradation of RhB and MB. Moreover, the monomers TPA-3BO and TPT-3BO are also unstable in water, which could further limit their effectiveness in photocatalytic applications. This instability likely contributes to their reduced degradation performance for both RhB and MB dyes, as the materials may degrade or lose their catalytic properties in the presence of water.

## Conclusions

In conclusion, novel donor–acceptor BTPP-TPA and BTPP-TPT CMPs were successfully developed for visible-light-driven ROS generation, enabling the efficient adsorption and photocatalytic degradation of cationic dyes, including RhB and MB. The synthesized CMPs exhibited excellent thermal stability, with *T*_d10_ values up to 573.91 °C and char yields up to 68.54%, as well as high BET surface areas reaching 521 m^2^ g^–1^. Among them, BTPP-TPT CMP showed superior adsorption performance toward RhB and MB in aqueous solution, achieving removal efficiencies of 98.29% and 98.24%, respectively, within 90 min. Moreover, BTPP-TPT CMP displayed excellent photocatalytic degradation activity, with apparent rate constants of 2.6 × 10^–2^ min^–1^ for RhB and 1.7 × 10^–2^ min^–1^ for MB. The enhanced performance of BTPP-TPT CMP is attributed to the synergistic effects of its extended π conjugation, intrinsic porosity, high surface area, larger pore volume, planar TPT unit, D–A framework, nitrogen-rich structure, and efficient charge-transfer behavior. These features promote dye adsorption, charge separation/transport, and ROS-mediated photocatalytic degradation. Therefore, these pyridine-based CMPs show strong potential as metal-free photocatalysts for wastewater treatment applications.

## Supplementary Material



## Notes

Materials, characterization methods, synthetic procedures, FT-IR and NMR spectra, XPS analysis, elemental analysis, TGA, BET, XRD, chemical-stability tests, UV–vis DRS, CV, photocurrent, EIS, contact-angle measurements, dye-adsorption experiments, photocatalytic degradation studies, cycling stability, pH and catalyst-dosage effects, scavenger experiments, LC-MS analysis, proposed degradation pathways, control experiments, comparison tables, and references (PDF)
