# Supplementary material for: Donor–Acceptor Pyridine-Based Conjugated Microporous Polymers for Visible-Light-Driven Reactive Oxygen Species Generation and Photocatalytic Dye Degradation
Source: ACS Polym Au. 2026 Jun 1;6(5):1286–300. doi: 10.1021/acspolymersau.6c00054 (PMC13673878; doi:10.1021/acspolymersau.6c00054)
Supplement: Supplementary file 1 [file lg6c00054_si_001.pdf]

Supporting Information

Donor–Acceptor Pyridine-Based Conjugated Microporous Polymers for Visible-Light-Driven Reactive Oxygen Species Generation and Photocatalytic Dye Degradation

Shimaa Abdelnaser, [a,b] M. Rashad,[c] Wei-Lung Tseng,[d] Shiao-Wei Kuo [a], Ahmed F. M. EL-Mahdy [a,b*]

[a] Department of Materials and Optoelectronic Science, National Sun Yat-Sen University, Kaohsiung 80424, Taiwan

[b] Chemistry Department, Faculty of Science, Assiut University, Assiut 71516, Egypt

[c] Physics Department, Faculty of Science, University of Tabuk, Tabuk 71491, Saudi Arabia

[d] Department of Chemistry, National Sun Yat-sen University, Kaohsiung 80424, Taiwan

Corresponding authors:

Ahmed F. M. EL-Mahdy, +886-986-404-402, E-mail: ahmedelmahdy@mail.nsysu.edu.tw

Section	Content	Page No.
S1	Materials	S3
S2	Characterization	S3
S3	Synthetic Procedures of Monomers	S5
S4	Synthetic Procedures of Polymers	S8
S5	Spectral Analyses of Monomers	S9
S6	X-Ray Photoelectron Spectroscopy Analysis	S13
S7	Elemental analysis	S15
S8	Thermogravimetric Analysis (TGA) Profile	S15
S9	BET Parameters	S15
S10	X-ray diffraction pattern (XRD) profile	S16
S11	Chemical Stability	S16
S12	Photophysical and electrochemical properties	S17
S13	Cyclic Voltammetry and Electronic band levels	S17
S14	Photocurrent Measurement	S18
S15	Contact Angle Measurements	S19
S16	Dye adsorption Experiments	S20
S17	Photodegradation	S23
S18	References	S46

S1. Materials

Terephthalaldehyde (98%), 4-bromoacetophenone (98%), chloroform ($CHCl_3$) and methanol (MeOH) were purchased from Alfa Aesar. Ammonium acetate (NH_4OAc) (97 %) was obtained from Showa. N-Bromosuccinimide (NBS, 99%), tetrakis(triphenylphosphine)palladium ($Pd(PPh_3)_4$, 99%), and 4-bromobenzonitrile (≥98%) were sourced from Acros. Combi-Blocks supplied bis(pinacolato)diboron (98%), potassium acetate, sodium carbonate (Na_2CO_3) and potassium carbonate (K_2CO_3). Additionally, Triphenylamine (TPA), 1,4-dioxane, acetic acid, trimethylamine and dimethylformamide (DMF) were obtained from Sigma-Aldrich. Acetone (99.9%) we purchased from Echo Chemical. Dimethyl sulfoxide (DMSO) was purchased from J. T. Baker. Tetrahydrofuran (THF, 99.9%) was purchased from HY Biocare Chem.

S2. Characterization

Fourier transform infrared (FTIR) spectra were recorded using a Nicolet iS50 FTIR spectrophotometer, with 32 scans collected at a resolution of 4 cm^{-1}. The sample preparation was conducted following the standard potassium bromide (KBr) disc method. All samples were prepared by compressing KBr discs, ensuring optimal transparency and consistency for FTIR analysis. Nuclear magnetic resonance (NMR) spectra were obtained on a JEOL ECZ 600R spectrometer, operating at 500 MHz for ^1H and 125 MHz for ^{13}C. Chemical shifts are reported in parts per million (ppm) relative to the internal standard. Solid-state nuclear magnetic resonance (ssNMR) spectra were acquired on a Bruker Avance III HD 400 MHz NMR spectrometer, equipped with a Bruker magic angle spinning (MAS) probe. A total of 32,000 scans were performed to ensure high-quality spectral data. X-ray photoelectron spectroscopy (XPS) measurements were conducted using a ULVAC PHI Quantes system to analyze the surface chemical composition and elemental states of the samples. Thermogravimetric analysis (TGA) was performed using a TA Q-50 analyzer under a continuous nitrogen flow. The

samples were heated from 40 to 800 °C at a constant rate of 20 °C min^{-1}, with nitrogen gas circulating at a flow rate of 60 mL min^{-1}. The analysis was conducted in a platinum (Pt) cell to ensure accurate thermal stability measurements. The specific surface area and porosity of the samples, ranging from 20 to 100 mg, were evaluated using a Micromeritics ASAP 2020 Surface Area and Porosity Analyzer. The analysis was based on the BET (Brunauer-Emmett-Teller) theory to interpret the surface area and pore structure data. Nitrogen adsorption isotherms were generated by incrementally exposing the samples to ultrahigh-purity nitrogen gas (up to approximately 1 atm) at 77 K in a liquid nitrogen bath. Powder X-ray diffraction (XRD) was carried out using a Karlsruhe X-ray powder diffractometer, utilizing monochromated Cu/Kα radiation with a wavelength of 0.1542 nm. Transmission electron microscope (TEM) was performed using a JEOL 2100 scanning electron microscope, operating at an acceleration voltage of 200 kV. Scanning electron microscopy (SEM) was performed using a ZEISS Gemini SEM 450 High-Resolution Scanning Electron Microscope. Prior to imaging, the samples were sputter-coated with a thin layer of gold (Au) for 40 seconds to enhance conductivity and improve image quality. Cyclic voltammetry (CV) and electrochemical impedance spectroscopy (EIS) measurements were carried out using a Squid stat Plus electrochemical workstation. UV–Vis–NIR spectra. Samples for UV–Vis and fluorescence spectroscopy were dissolved in suitable organic solvents and placed in a small quartz cell (0.2 × 1.0 × 4.5 cm^3). UV–Vis–NIR spectra were recorded at 25 °C using a Jasco V-570 spectrometer (Japan), with deionized water as the solvent. Photoluminescence (PL) was measured by HITACHI F-4500, using a 150W Xe Lamp. The samples were mixed with DMF then placed in a small quartz cell and excited with 360 nm wavelength. The photodegradation of dye was performed using a solar simulator of 7IS0503A, SOFN Instruments Co. Ltd.

S3. Synthetic Procedures of Monomers

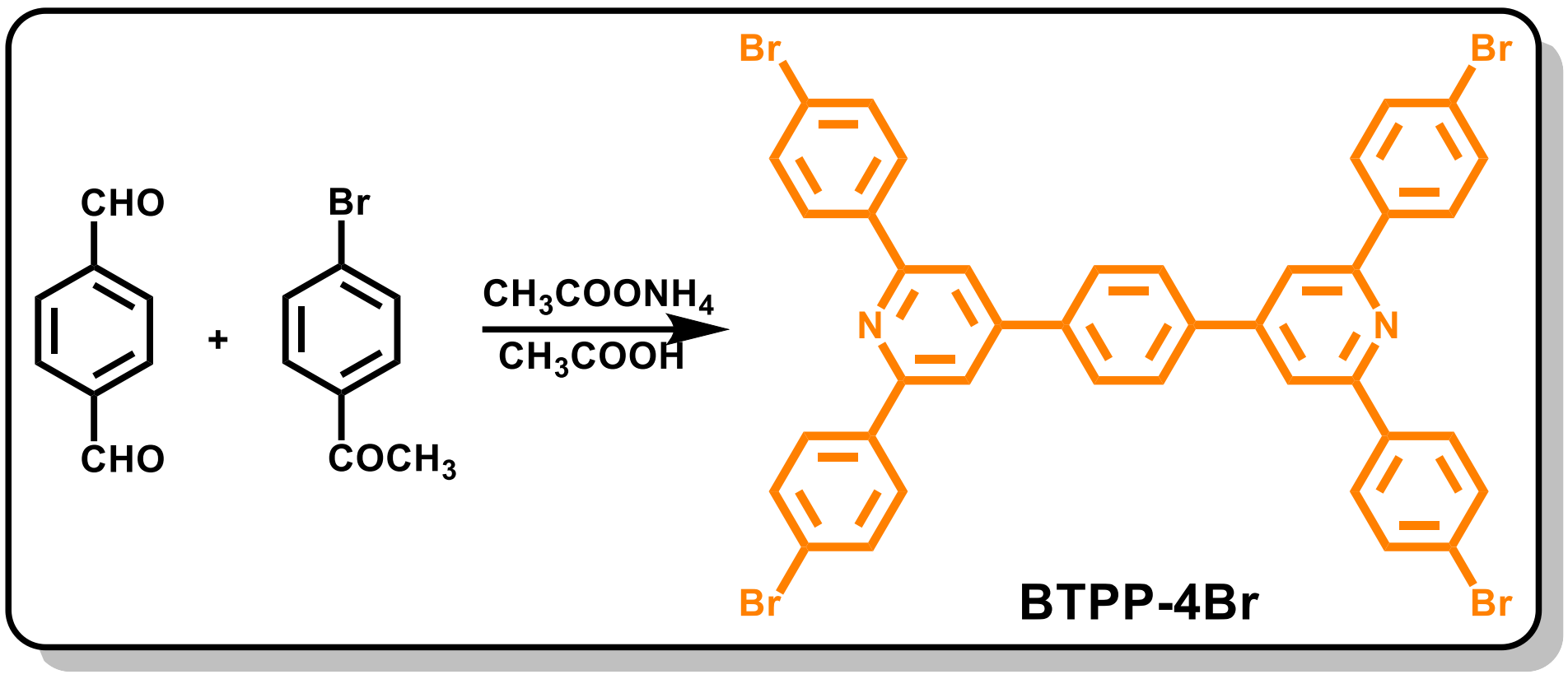

Scheme S1. Synthesis of 1,4-bis(2,6-bis(4-bromophenyl)pyridin-4-yl)benzene (BTPP-4Br).

1,4-bis(2,6-bis(4-bromophenyl)pyridin-4-yl)benzene (BTPP-4Br): A 20-mL microwave vial equipped with a stirrer bar was charged with 4-bromoacetophenone (9 g, 45.2 mmol), terephthaldehyde (1 g, 7.5 mmol), ammonium acetate (23 g, 298.4 mmol), and acetic acid (14.6 mL, 0.260 mmol). The contents were heated in a microwave oven under autogenous pressure at 500 W and 220 °C for 90 min. After cooling to room temperature, deionized water was added. The aqueous suspension was neutralized through the addition of a solution of $NaHCO_3$. The aqueous phase was extracted with DCM; the combined organic extracts were dried (anhydrous $MgSO_4$) and filtered. The solvent was evaporated under reduced pressure; the residue was recrystallized (acetone) and dried under vacuum overnight to yield a yellow solid of BTPP-4Br [1]. FT-IR (KBr disks): 3069, 1599, 1546, 1176, 625 cm^{-1}. ^{1}H NMR (CDCl3, 25 °C, 500 MHz, δ, ppm): 8.10 (d,s, aromatic, 12H), 7.93 (d, aromatic, 8H), 7.66 (d, aromatic, 4H). ^{13}C NMR (CDCl3, 25 °C, 125 MHz, δ, ppm):156.59, 139.43, 137.65, 131.97, 129.53, 128.78, 128.05, 124, 117.18.

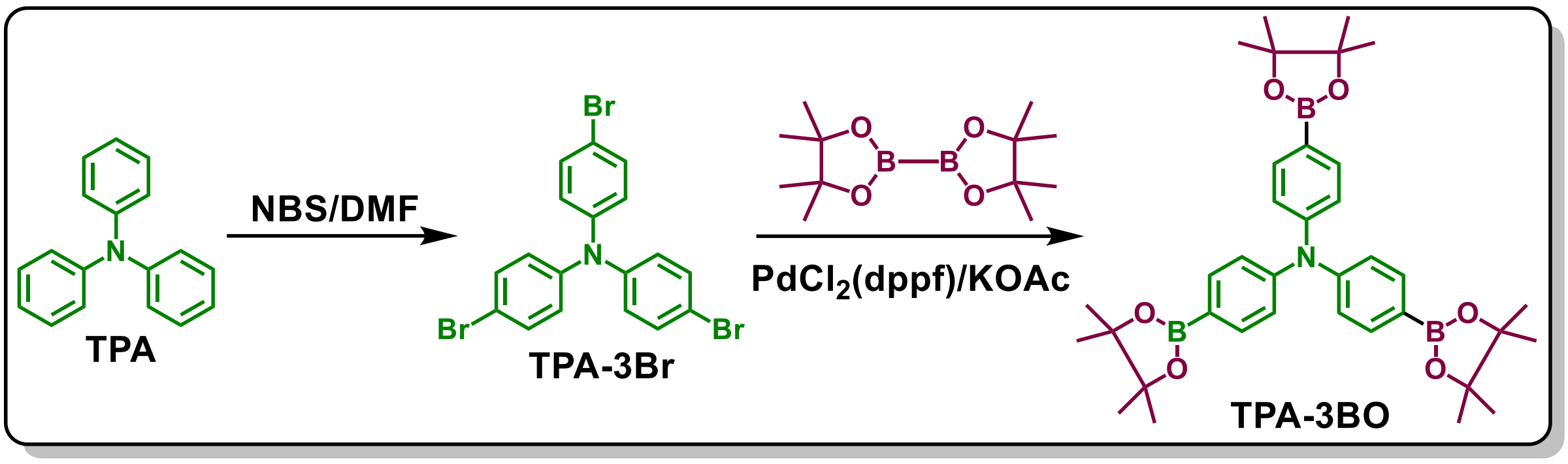

Scheme S2. Synthesis of tris(4-(4,4,5,5-tetramethyl-1,3,2-dioxaborolan-2-yl)phenyl)amine (TPA-3BO).

Tris(4-bromophenyl) amine (TPA-3Br): In a beaker *N*-bromosuccinimide (NBS; 12 g, 12.32 mmol) was dissolved into DMF (60 ml). Another solution of trimethylamine (5 g, 4.080 mmol) was mixed with DMF (120 ml) at 0 °C. The first solution (NBS) was dropped wise onto the second then the mixture was left under stirring at room temperature overnight. The mixture was sediment via pouring onto cold water then extracted by DCM followed by washing several cycles by methanol, white precipitate was granted. FTIR: 3064, 1570, 1266, 1081, 995, and 816 cm^{-1}. ^{1}H NMR (500MHz, r.t., $CDCl_3$): δ 7.35 (d, J = 8.8, 6H), 6.921 ppm (d, J = 8.8, 6H). ^{13}C NMR ($CDCl_3$, 25 °C, 125 MHz): 146.7, 132.8, 126.01 and 116.38 ppm.

Tris(4-(4,4,5,5-tetramethyl-1,3,2-dioxaborolan-2-yl)phenyl)amine (TPA-3BO): TPA-3Br (734 mg, 1.54 mmol), potassium acetate (4.8 g, 46 mmol), bis(pinacolato)diboron, (1.56 g, 6.14 mmol) and bis(diphenylphosphino)-ferrocene]dichloropalladium (101 mg, 0.138 mmol) were evacuated in a 250 mL 2-neck flask for 15 min, after this time a dry dioxane (50 mL) was injected to the reaction then refluxed for 24 hours under N_2 pressure. Finally, the TPA-3BO was obtained by DCM then purified by column chromatography PE-DCM (V/V, 3:1) [2]. FTIR (KBr disks): 3048, 2975, 1603, 1363, and 1285 cm^{-1}. ^{1}H NMR ($CDCl_3$); 7.68 (d, 6H), 7.05 (d, 6H), and 1.34 ppm (s, 36 H). ^{13}C NMR ($CDCl_3$): 150.26, 136.59, 124.34, 84.18, and 24.94 ppm.

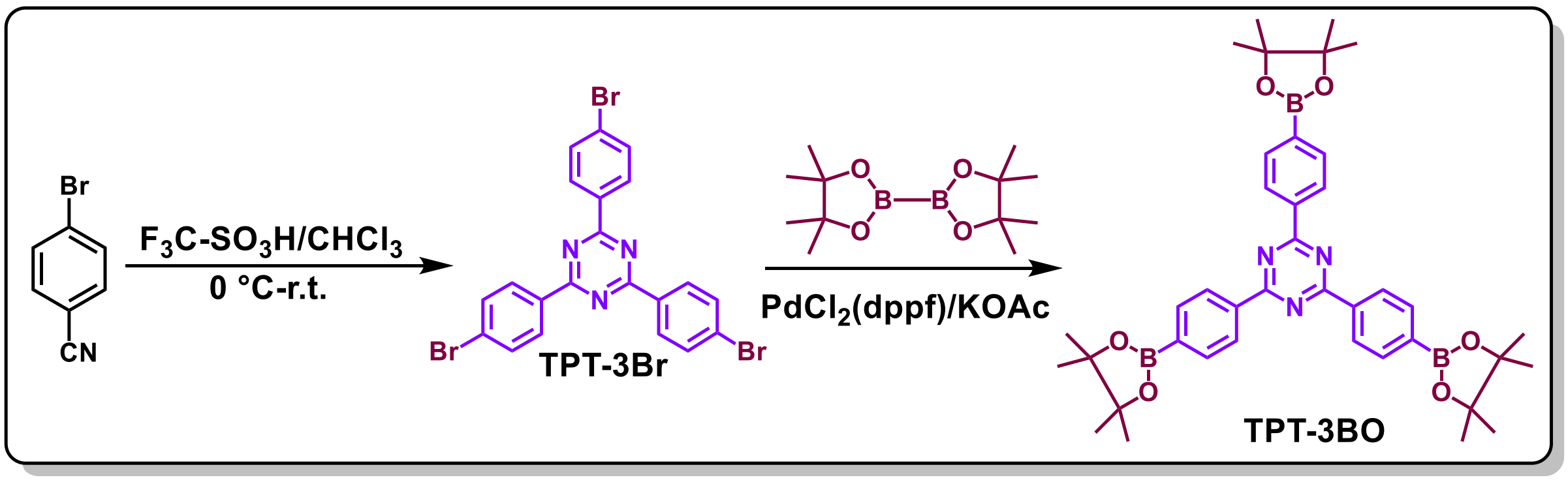

Scheme S3. Synthesis of 2,4,6-tris(4-(4,5-dimethyl-1,3,2-dioxaborolan-2-yl)phenyl)-1,3,5-triazine (TPT-3BO).

2,4,6-tris(4-bromophenyl)-1,3,5-triazine (TPT-3Br): In a 100 mL two neck-bottle under nitrogen atmosphere, a solution of 4-bromobenzonitrile (1.5 g, 8.24 mmol) in dry chloroform (20 mL) was cooled to 0 °C and then trifluoromethanesulfonic acid (4 mL, 0.045 mmol) was drop wisely added. The reaction mixture was stirred for 30 min at 0 °C and then warmed to room temperature. After stirring for an additional 20 hours at room temperature, the reaction solution was poured into ice-water (50 mL). The aqueous solution was neutralized by sodium carbonate (Na_2CO_3). The formed precipitate was collected by filtration and dried under vacuum overnight to yield 2,4,6-tris(4-bromophenyl)-1,3,5-triazine as a white solid. FT-IR (powder): 1578, 1518, 1400, 1372, 1173, 1069, 1011, 843, 806, 498 cm^{-1}. ^{1}H NMR ($CDCl_3$, 25 °C, 500 MHz): 8.60 (d, J = 8.5 Hz, 6H), 7.70 (d, J = 8.5Hz, 6H). ^{13}C NMR ($CDCl_3$, 25 °C, 125 MHz): 171.22, 134.91, 132.01, 130.48, 127.88.

2,4,6-tris(4-(4,5-dimethyl-1,3,2-dioxaborolan-2-yl)phenyl)-1,3,5-triazine (TPT-3BO): In a 100 mL two neck-bottle under nitrogen atmosphere, a solution of TPT-3Br (0.60 g, 1.10 mmol), 1,1′-Bis(diphenylphosphino) ferrocene]dichloropalladium(II) (0.50 g, 0.70 mmol), potassium acetate (0.30 g, 3.30 mmol), and bis(pinacolato)diboron (0.85 g, 3.65 mmol) in 1,4-dioxane (50 mL) was heated at 100 °C 24 h under. After consumption of the TPT-3Br, the reaction mixture was cooled to room temperature and then poured into iced-water. The formed precipitate was collected by extraction with dichloromethane and dried

under vacuum overnight. The residue was purified by flash chromatographically (SiO_2; THF/Hexane = 1:4) to provide a white solid (0.56 g, 75.0%) [2]. FTIR (KBr, cm^{-1}): 2975–2870, 1572, 1511, 1349. ^1H NMR ($CDCl_3$, 25 °C, 500 MHz): 8.62 (d, 6H, J = 8.0 Hz), 7.90 (d, 6H, J = 8.0 Hz), 1.32 (s, 36H). ^{13}C NMR ($CDCl_3$, 25 °C, 125 MHz): 172.53, 139.34, 135.73, 128.67, 84.47, 24.49.

S4. Synthetic Procedures of CMPs

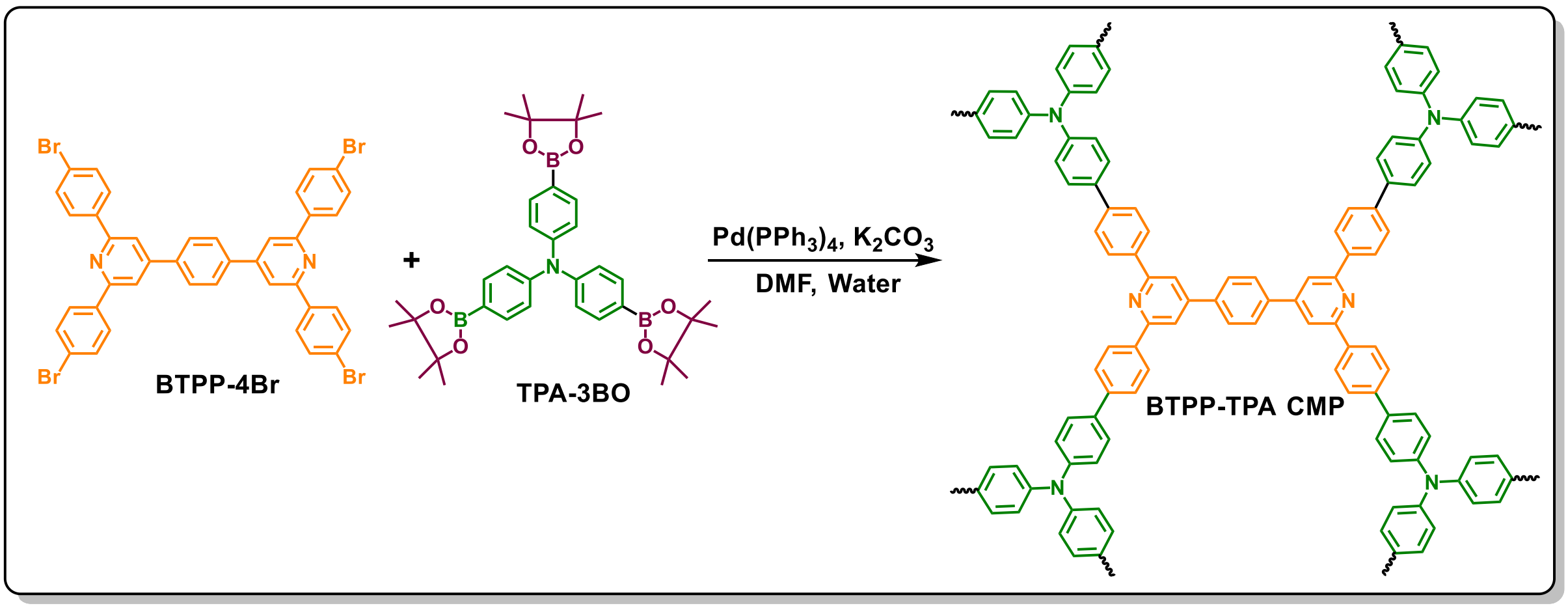

Scheme S4. Synthesis of BTPP-TPA CMP.

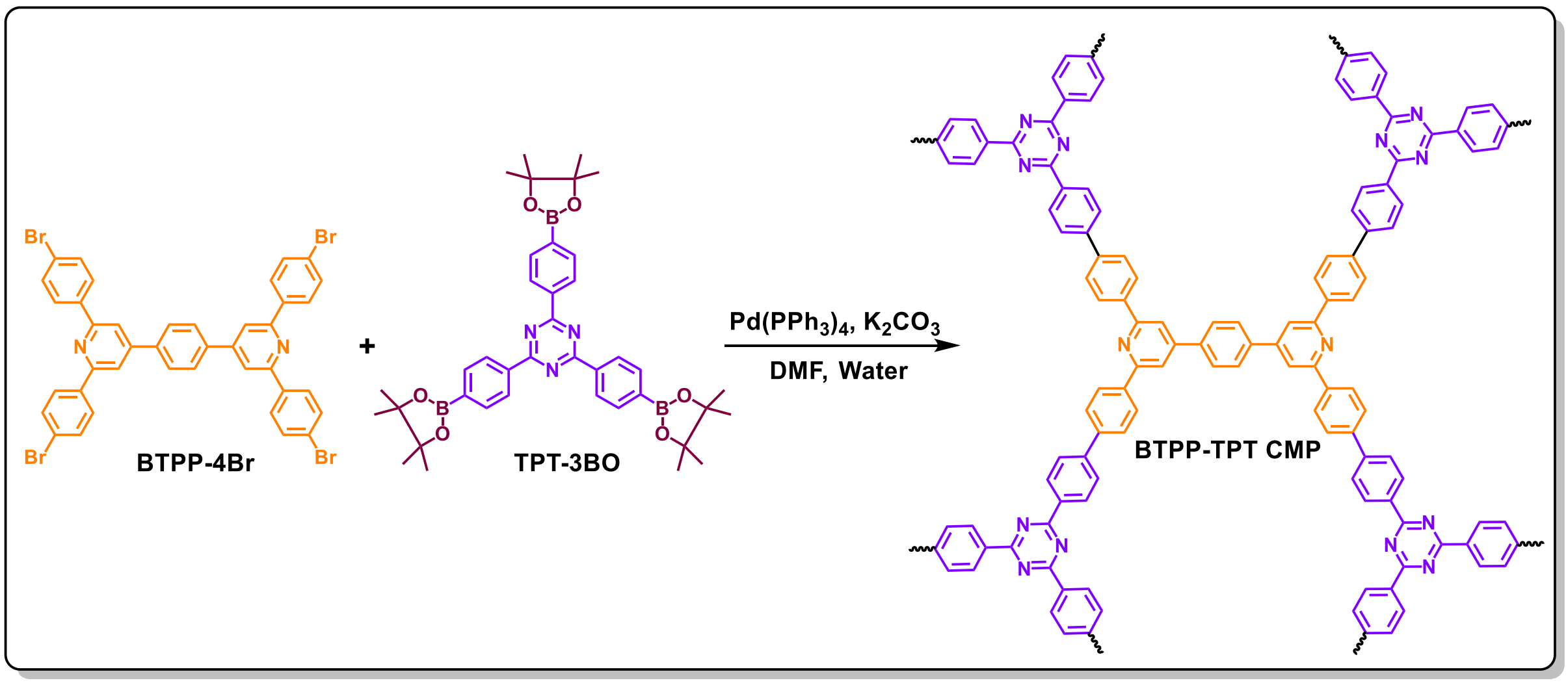

Scheme S5. Synthesis of BTPP-TPT CMP.

S5. Spectral Analyses of Monomers

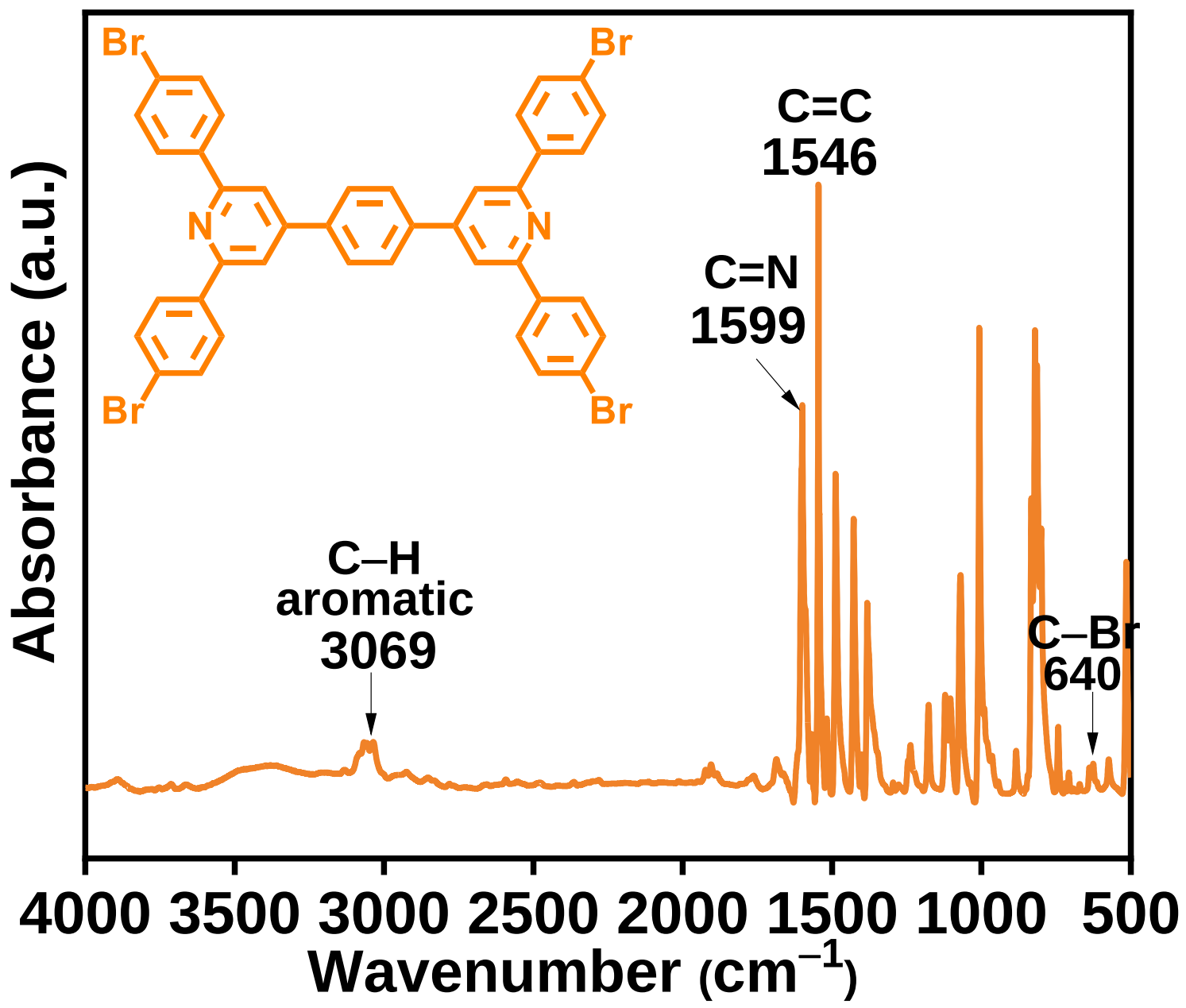

Figure S1. FTIR spectrum of BTPP-4Br.

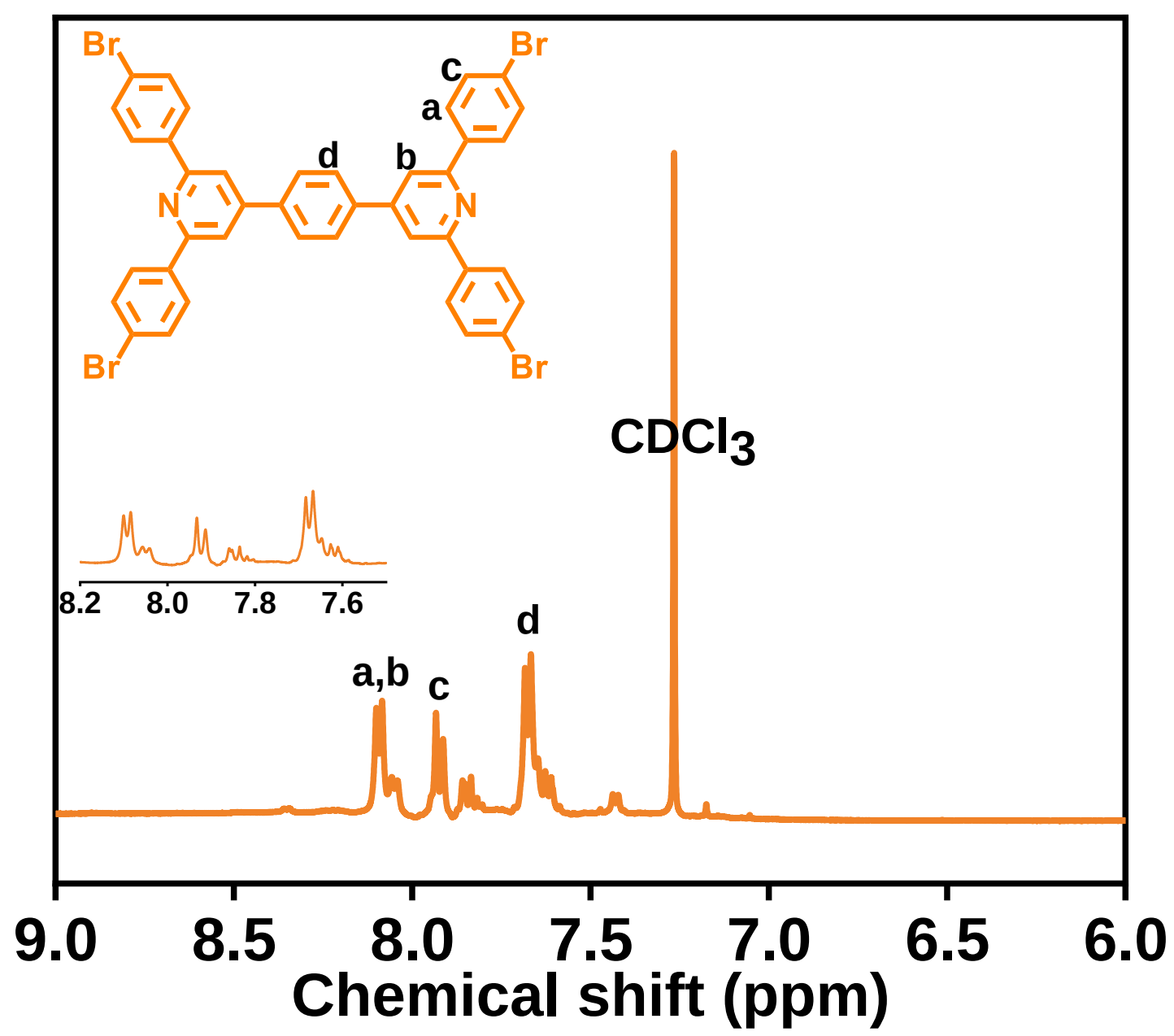

Figure S2. ^{1}H NMR spectrum of BTPP-4Br.

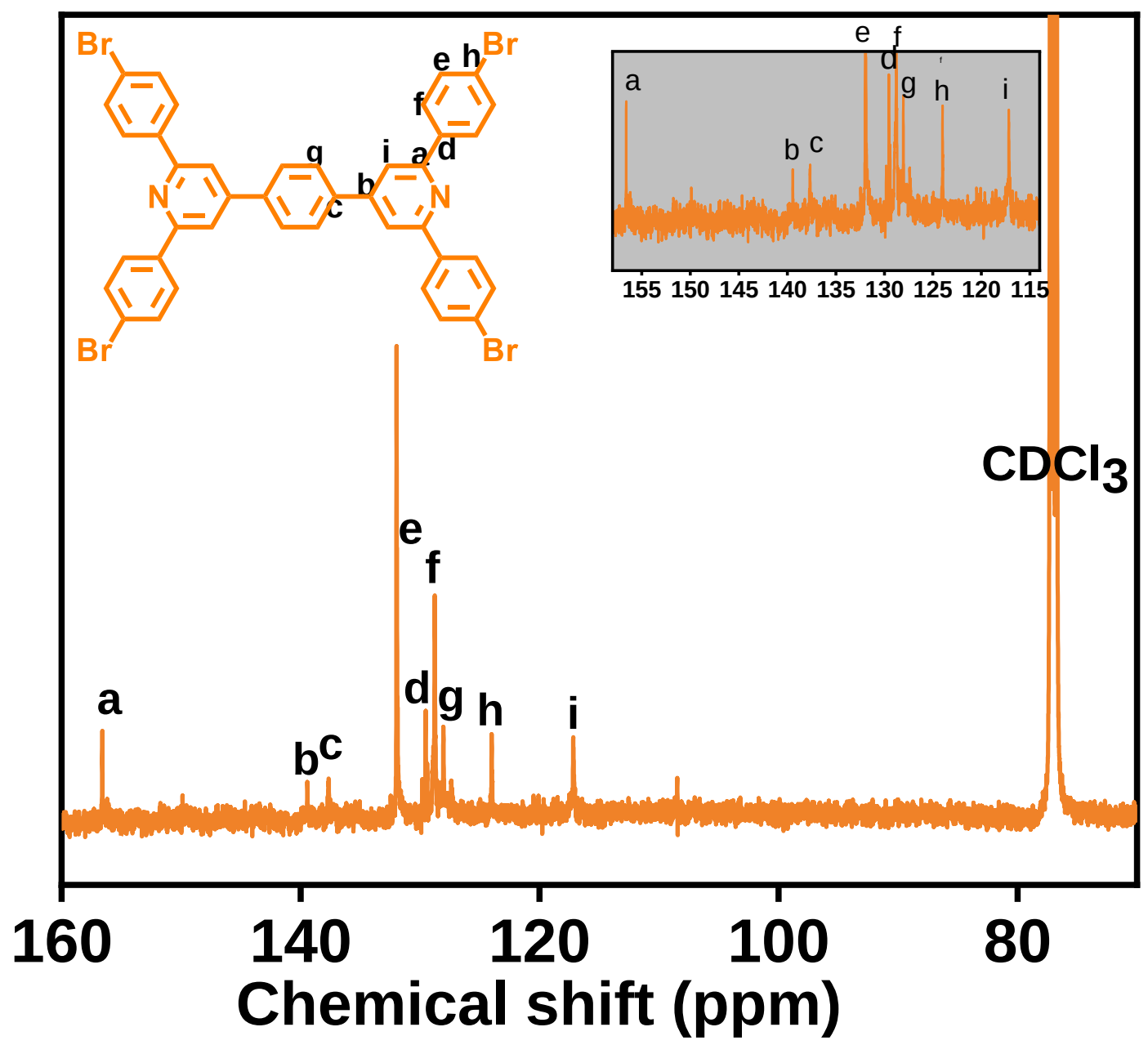

Figure S3. ^{13}C NMR spectrum of BTPP-4Br.

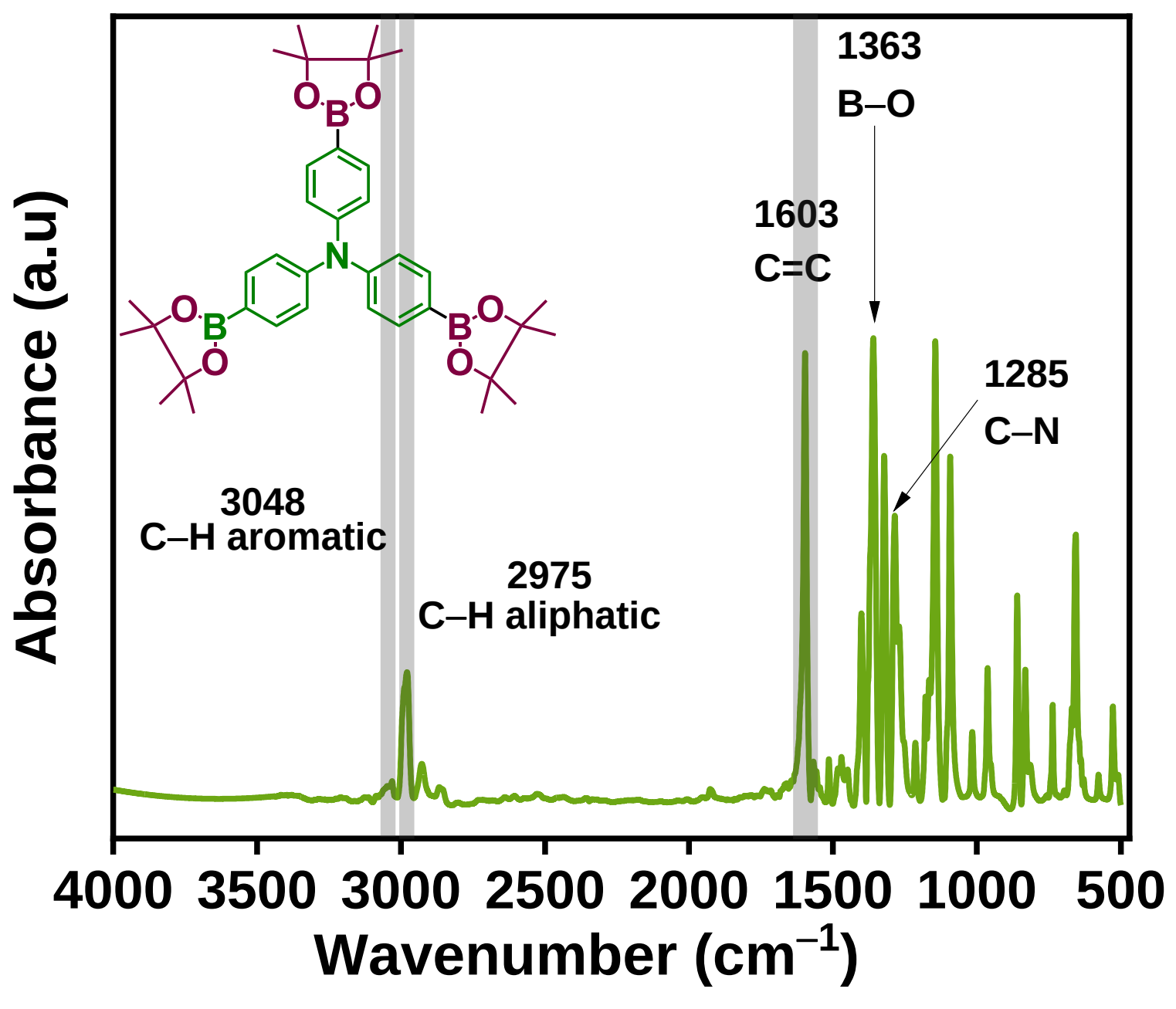

Figure S4. FTIR spectrum of TPA-3BO.

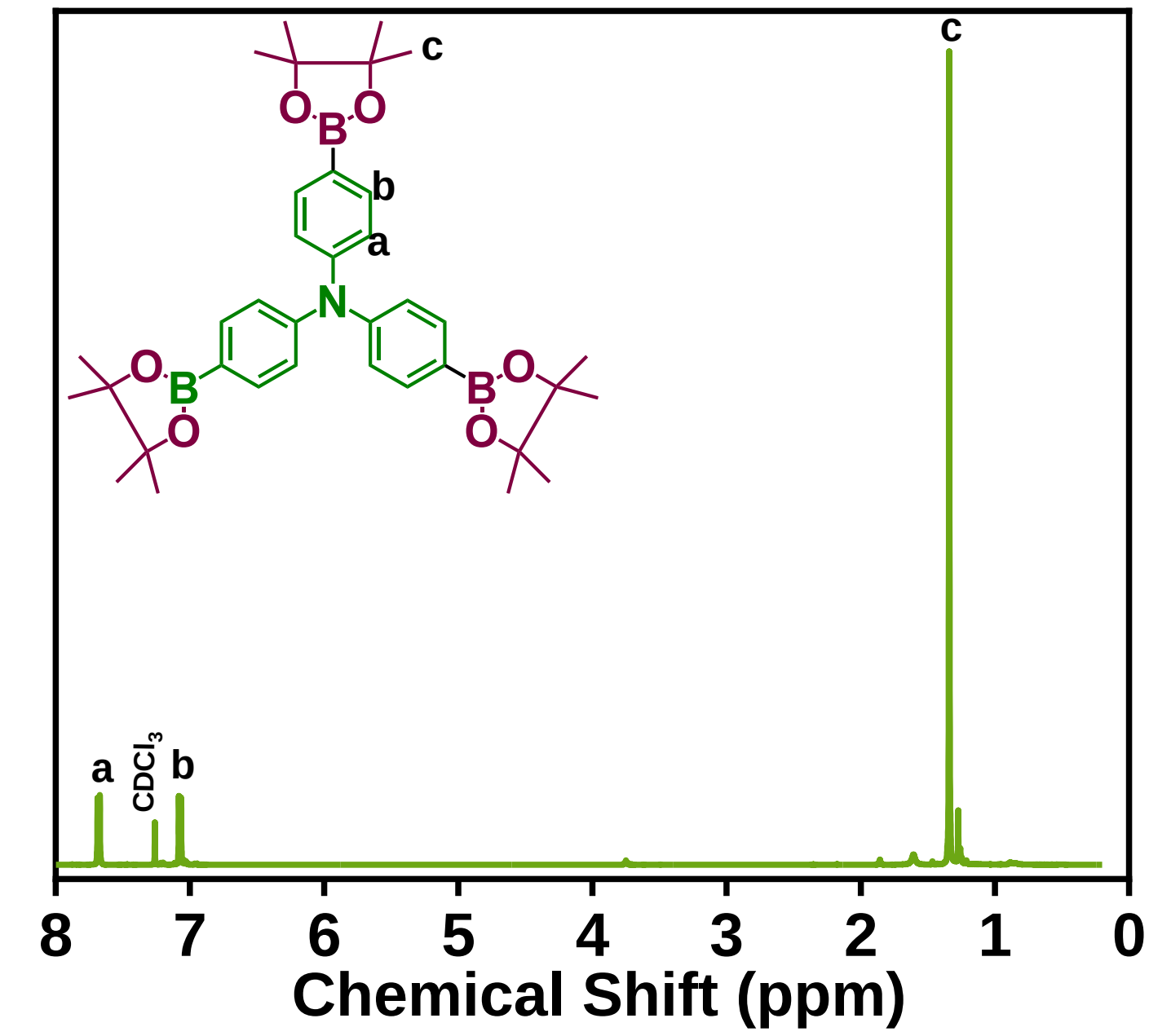

Figure S5. ^{1}H NMR spectrum of TPA-3BO.

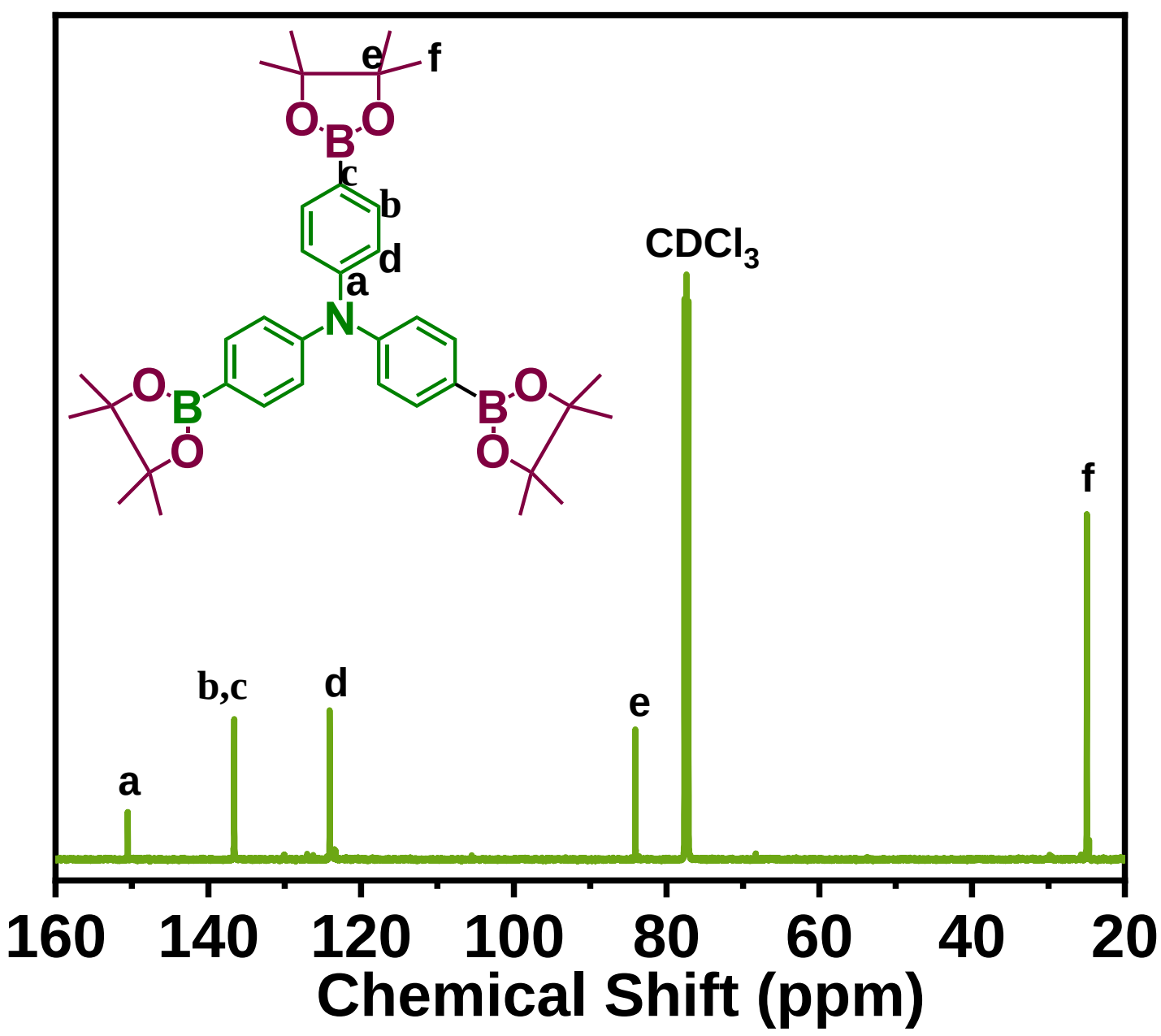

Figure S6. ^{13}C NMR spectrum of TPA-3BO.

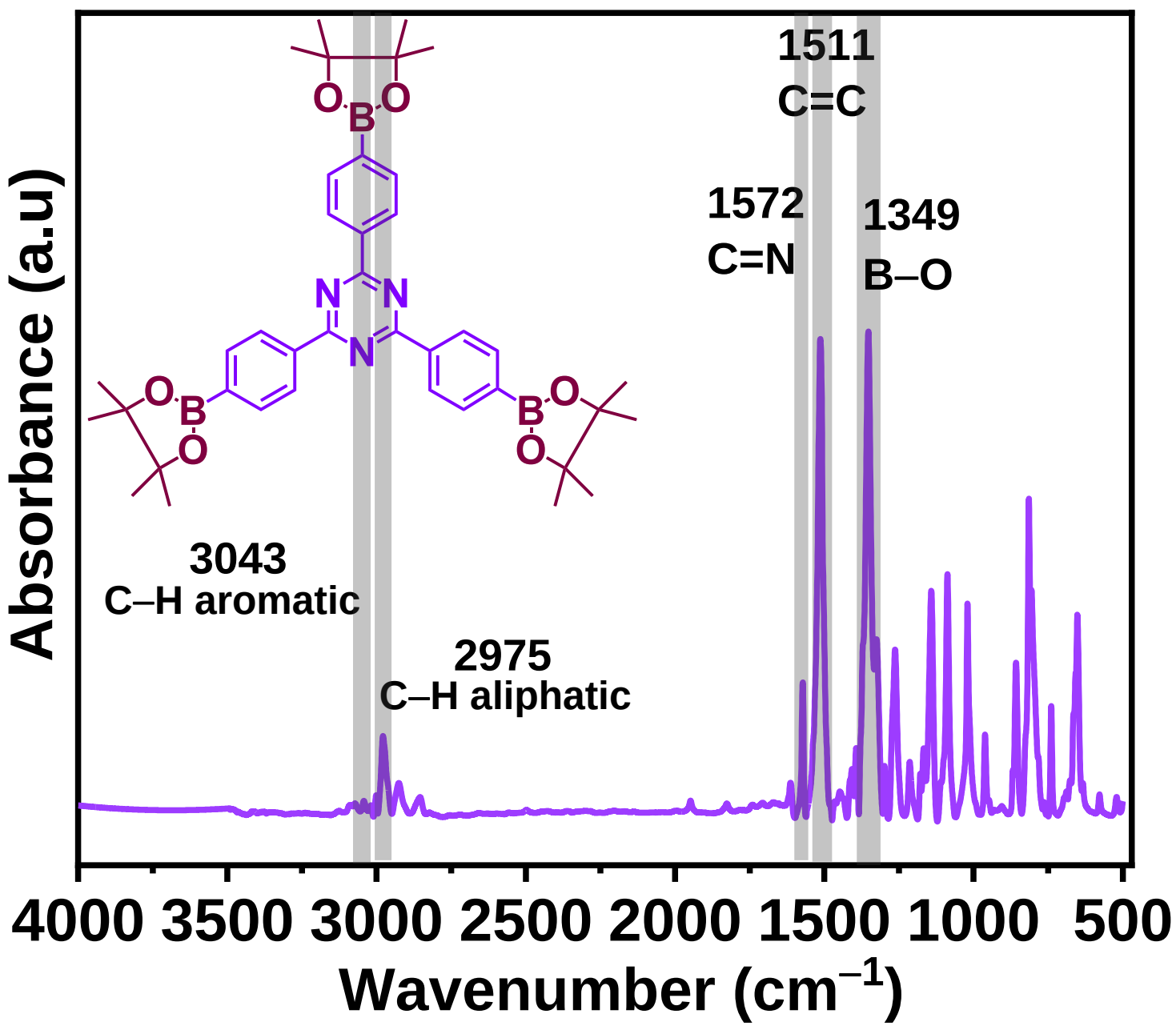

Figure S7. FTIR spectrum of TPT-3BO.

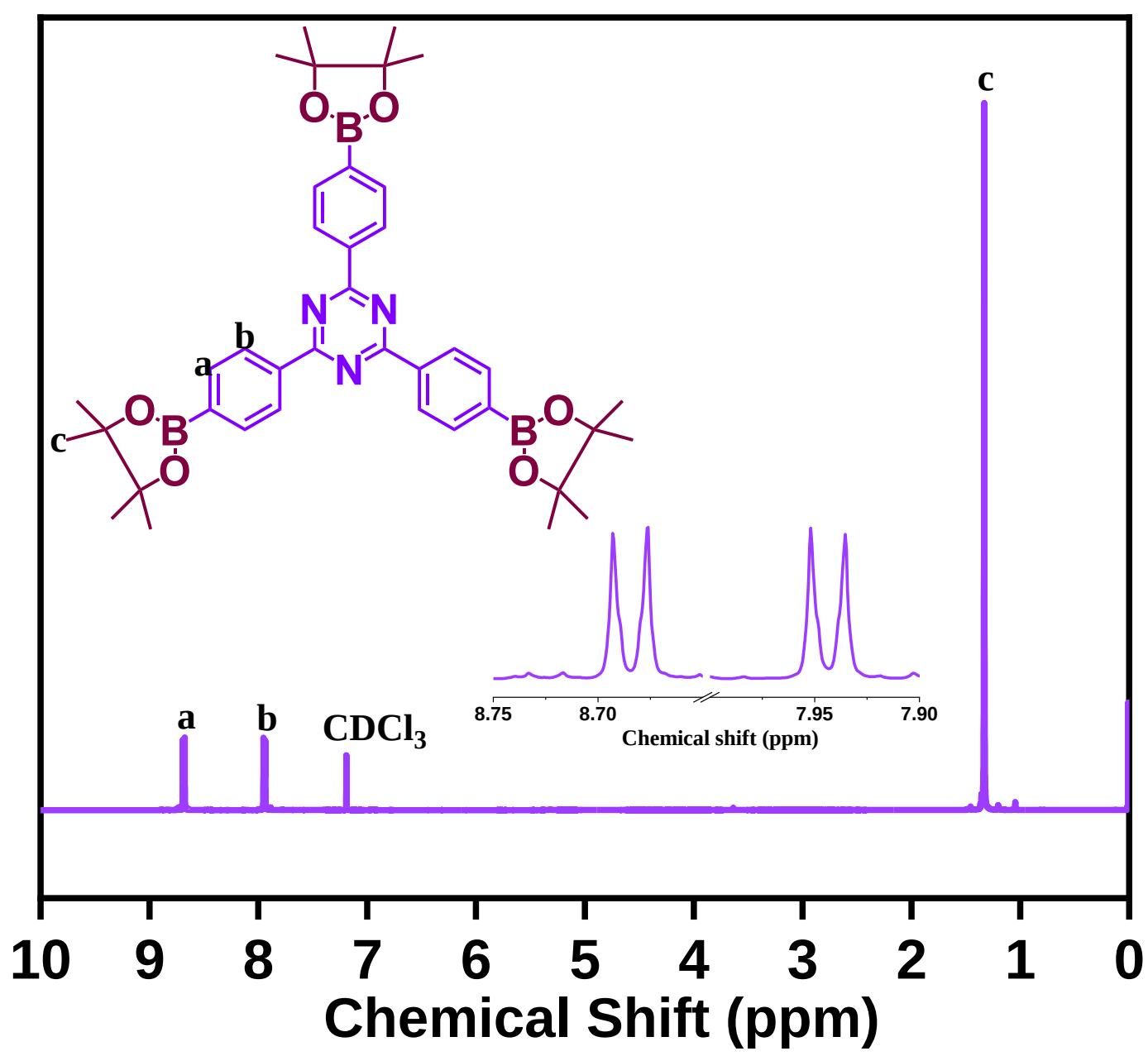

Figure S8. ^{1}H NMR spectrum of TPT-3BO.

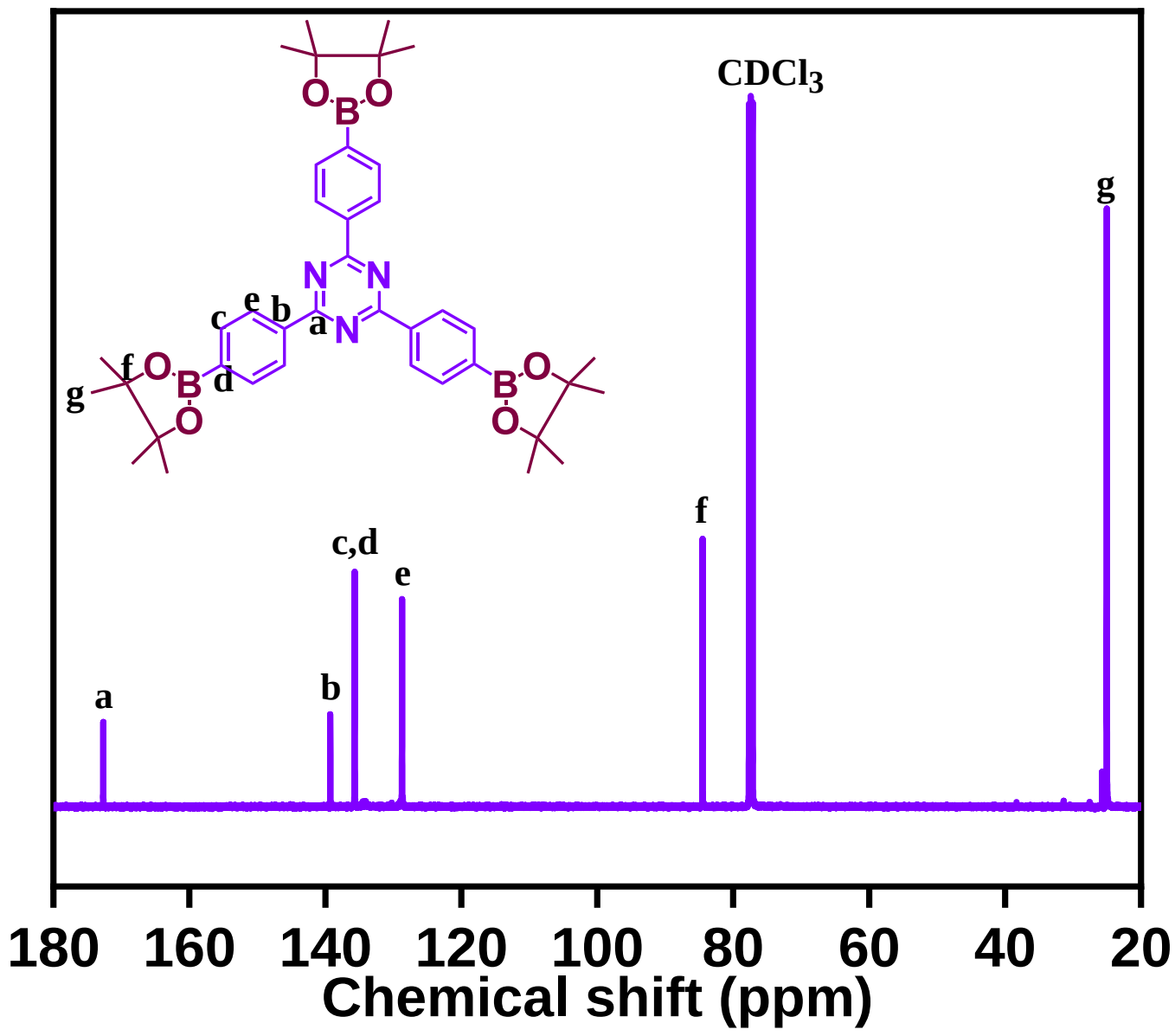

Figure S9. ^{13}C NMR spectrum of TPT-3BO.

S6. X-Ray Photoelectron Spectroscopy Analysis

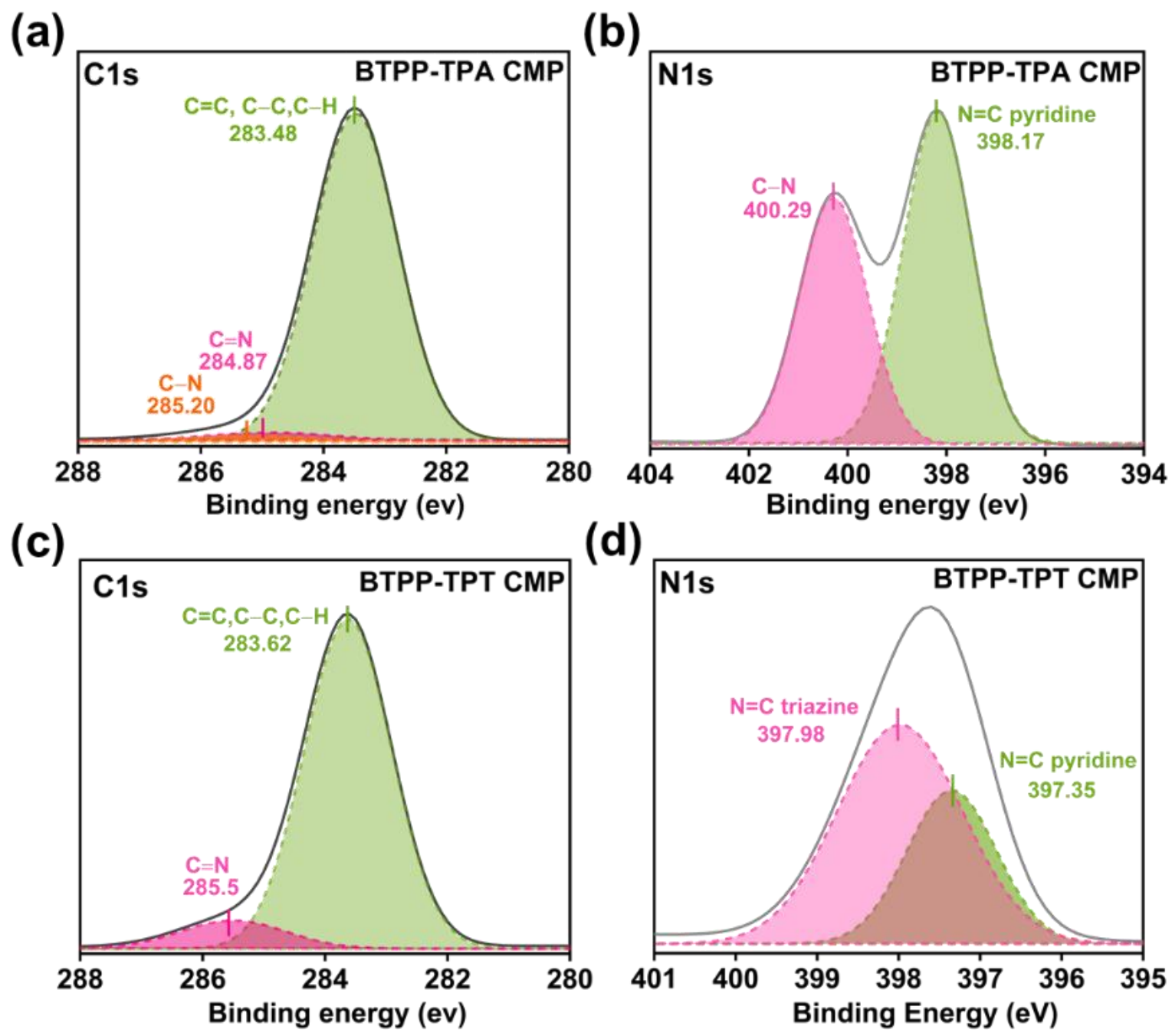

Figure S10. (a-d) XPS spectra of the (a,c) C1s and (b,d) N1s of (a,b) the BTPP-TPA CMP and (c,d) the BTPP-TPT CMP.

Table S1. XPS fitting positions of BTPP-TPA and BTPP-TPT CMPs.

Samples		C species			N species		
		C=C/C–C/C–H	C=N	C–N	N=C pyridine	N–C	N=C triazine
CMPs	BTPP-TPA	283.48	284.87	285.20	398.17	400.29	....
	BTPP-TPT	283.62	285.50	-------	397.35	-------	397.98

Table S2. XPS fitting ratio (%) of BTPP-TPA and BTPP-TPT CMPs.

Samples		%C species			%N species		
		C=C/C–C/C–H	C=N	C–N	N=C pyridine	N–C	N=C triazine
CMPs	BTPP-TPA	93.59	3.45	2.96	57.33	42.67	....
	BTPP-TPT	90.48	9.52	-------	33.49	-------	66.51

Table S3. Experimental element ratio (At %) of BTPP-TPA and BTPP-TPT CMPs.

Samples		Experimental element ratio (At %) *		
		C	N	O
CMPs	BTPP-TPA	88.76	4.49	6.75
	BTPP-TPT	85.02	8.28	6.69

* Excluding hydrogen atoms.

Table S4. Theoretical element ratio (At %) of BTPP-TPA and BTPP-TPT CMPs.

Samples		Theoretical element ratio (At %) *		
		C	N	O
CMPs	BTPP-TPA	89.28	4.42	6.3
	BTPP-TPT	85.65	8.14	6.2

* Excluding hydrogen atoms.

S7. Elemental analysis

Table S5. Elemental analysis results of BTPP-TPA and BTPP-TPT CMPs

CMP/Elements	C (%)	N (%)	O (%)	H (%)
BTPP-TPA	81.5	4.12	6.2	4.78
BTPP-TPT	77.6	7.56	6.11	4.32

S8. Thermogravimetric Analysis (TGA) profile

Table S6. Values of $T_{d10\%}$ and Char yield of BTPP-TPA and BTPP-TPT CMPs.

CMP	T_{d10}(°C)	Char yield (%)
BTPP-TPA	530.27	65.85
BTPP-TPT	573.91	68.54

S9. BET Parameters

Table S7. BET parameters of the synthesized CMPs.

CMP	SBET (m^2 g^{-1})	Pore volume (cm^3 g^{-1})	Pore size (nm)
BTPP-TPA CMP	300.16	0.49	0.67~1.58, 1.58~2.05, 2.05~2.69, 2.69~3.59
BTPP-TPT CMP	521	0.99	1.07~1.65 , 1.65~2.34

S10. X-ray diffraction pattern (XRD) profile

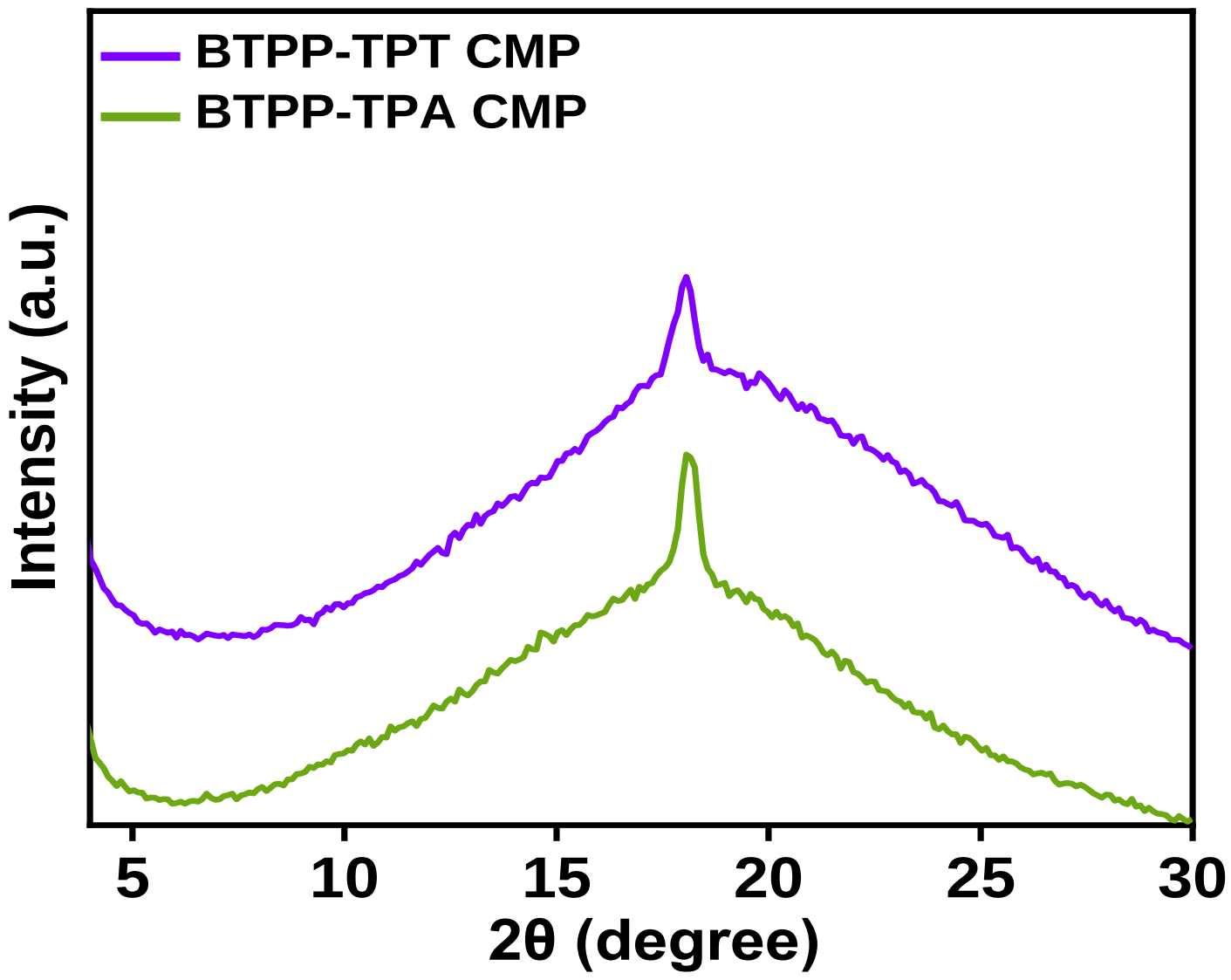

Figure S11. XRD spectra of the BTPP-TPA and the BTPP-TPT CMPs.

S11. Chemical Stability

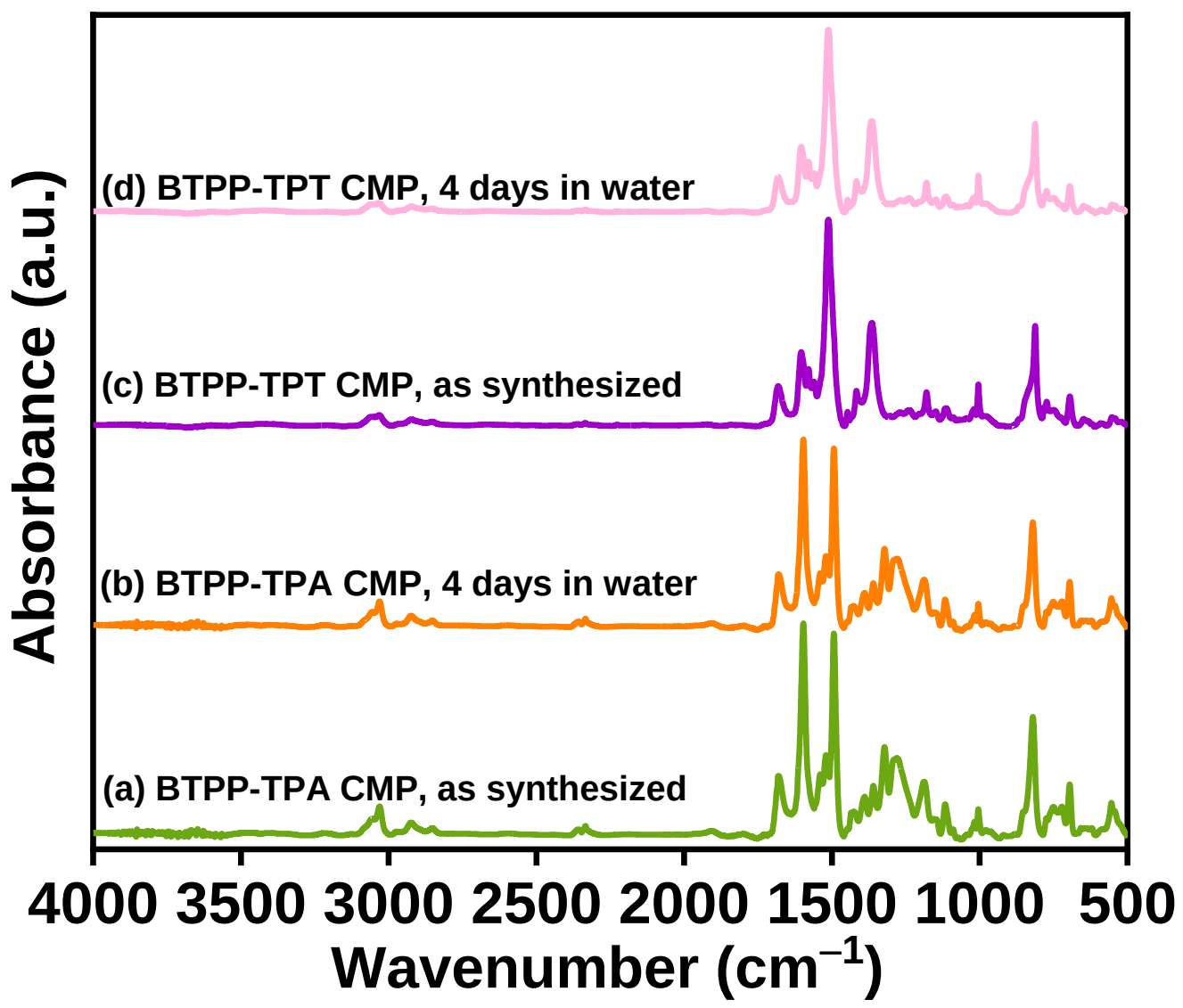

Figure S12. FTIR spectra of (a,c) BTPP-TPA and BTPP-TPT CMPs as-synthesized and (b,d) after immersing 4 days in water.

S12. Photophysical and electrochemical properties

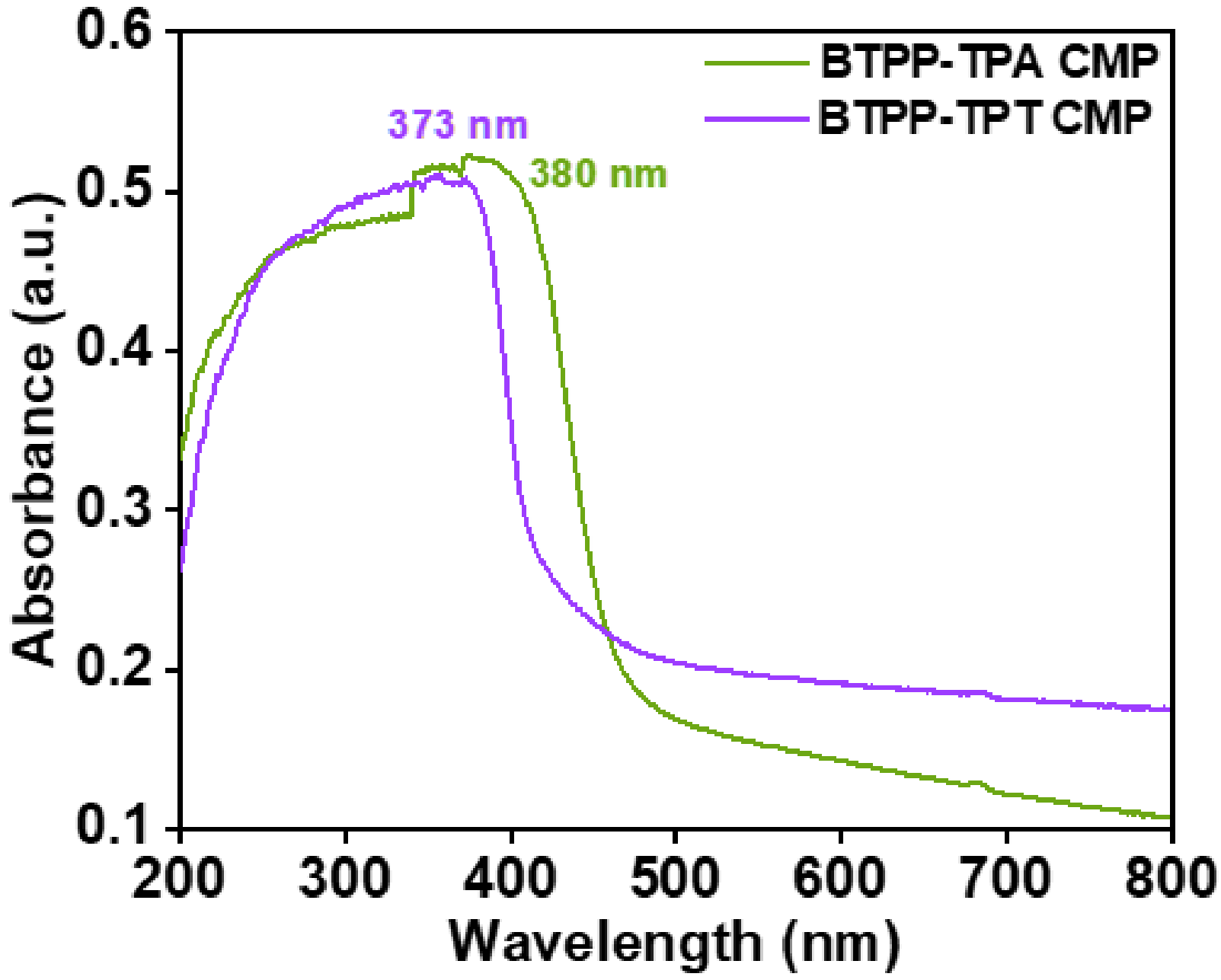

Figure S13. UV–Vis DRS spectra of the BTPP-TPA and the BTPP-TPT CMPs.

S13. Cyclic Voltammetry and Electronic band levels

Cyclic voltammetry (CV) measurements were carried out using Zahner Zennium 6273E workstation with three electrode configurations. The electrolyte is 0.1 M tetrabutylammonium hexafluorophosphate ($TBAPF_6$) using distilled acetonitrile as solvent. CHI 102 platinum working electrode (2 mm diameter) was used as the working electrode, RE-7 Ag/AgCl (0.1 M Ag/Ag+ acetonitrile solution) reference electrode was used as the reference electrode and platinum wire electrode was used as the counter electrode. 5 mg of the catalyst was mixed with isopropyl alcohol mixture (10 mL), subsequently added 25 µL of 5 wt. % Nafion to it and sonicated in a bath sonicator for 1h. 10 µL of the catalyst ink was drop cast on the working carbon electrode surface and air dried overnight.

For optical band gaps for BTPP-TPA and BTPP-TPT CMPs, the Tauc plots were performed according to the Kubelka–Munk function [3], [4]. The following equation is used to calculate optical band gap using absorption spectra:

Where *α* is the optical absorption coef $\alpha h\nu = A(h\nu - Eg)^{n/2}$ nt, ν is the light frequency, *Eg* is the optical band gap, and *A* is a constant depending on electron-hole mobility. The value of n is 1. Then, The Eg of the samples could well be determined from a plot of $(\alpha h\nu)^{1/2}$ versus energy (hν), and its value can be derived from the intercept of the tangent to the X-axis.

Table S8. Absorption maxima and energy levels of the BTPP-TPA and BTPP-TPT CMPs.

Photocatalyst	λ_{max}[nm][a]	E_g[eV][b]	LUMO[eV][c]	HOMO[eV][d]
BTPP-TPA CMP	380	2.66	–3.38	–6.04
BTPP-TPT CMP	373	2.97	–3.1	–6.07

[a] Absorption was determined in the solid state using reflectance mode; [b] Determined by the onset of UV-vis absorption; [c] Measured in acetonitrile/$TBAPF_6$ (0.1 M) solution, CHI 102 platinum working electrode was used as the working electrode, platinum wire as the counter electrode, Ag/AgCl reference electrode as the reference electrode, and Fc/Fc+ as an internal standard. Estimated vs vacuum level from LUMO = – [E_{red} + 4.40] eV; [d] Calculated from HOMO = LUMO – Eg.

S14. Photocurrent Measurement

The photocurrent response measurements were performed on a Zahner Zennium 6273E workstation equipped with visible-light irradiation with a conventional three-electrode cell including a Pt wire counter electrode, Ag/AgCl as reference electrode (3 M NaCl) and an indium tin oxide (ITO) glass as working electrode. About 5 mg of BTPP-CMPs were dispersed into an acetonitrile solution (1 mL) with 30 µL Nifion and sonicate for 30 min to obtain a slurry mixture. After that, 200 µL of as-prepared slurry was spread onto ITO glass with an active area of 6.875 cm^2. Here, 0.5 M Na_2SO_4 aqueous solution was prepared as electrolyte. 0.8 V constant potential was applied with the 30 s light on-off after a certain time interval to record the photo and dark current. A 300 W Xe lamp with a visible-light band-pass filter (λ > 450 nm) was employed as the excitation light source.

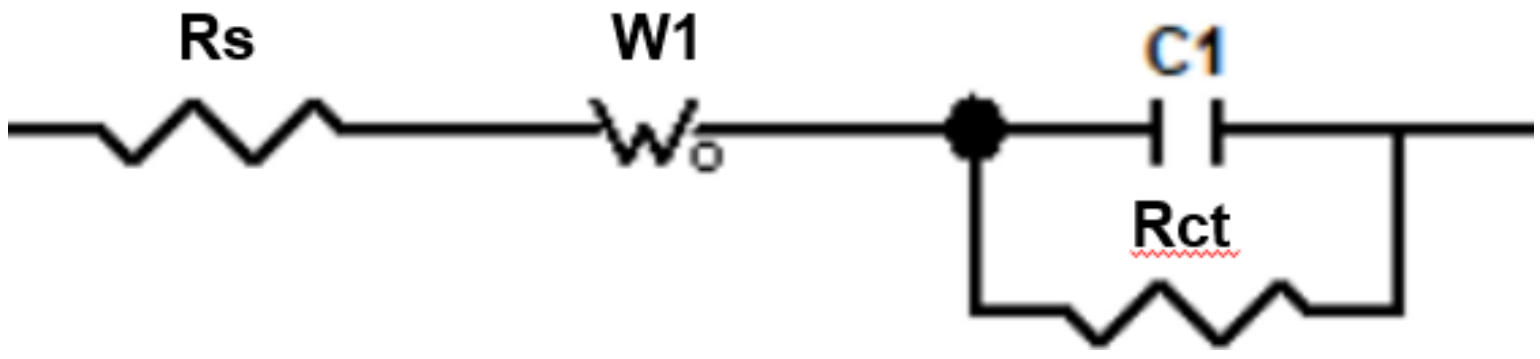

Figure S14. EIS and the fitted Nyquist plot of the BTPP-TPA and BTPP-TPT CMPs.

Table S9. The fitting values of Nyquist plots of the BTPP-TPA and BTPP-TPT CMPs.

CMP	C1 (F)	Rs (Ω)	Rct1 (Ω)
BTPP-TPA CMP	6.32E-6	23.06	500
BTPP-TPT CMP	5.59E-5	18.71	280

S15. Contact Angle Measurements

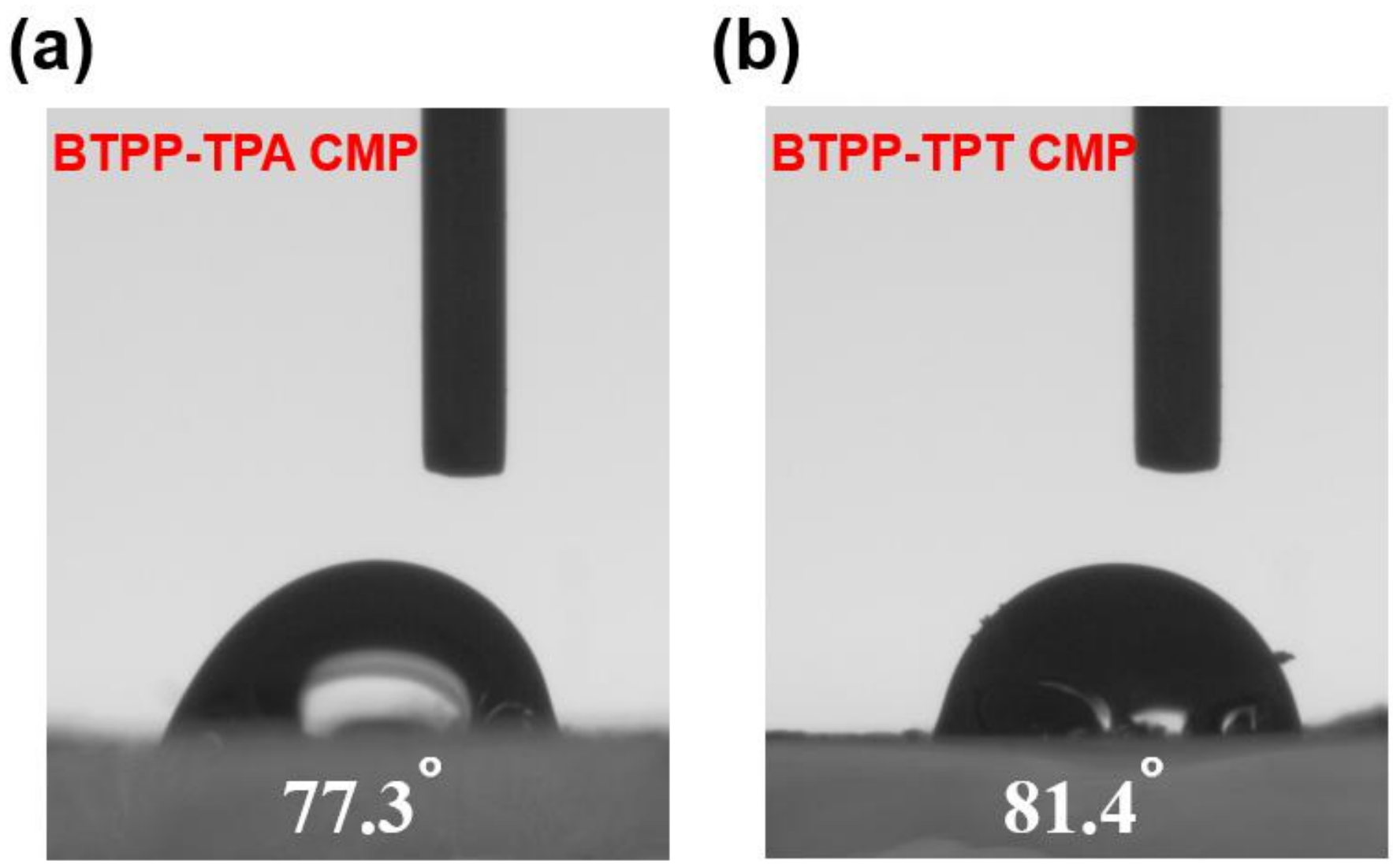

Figure S15. Contact Angle Measurements of (a) BTPP-TPA, and (b) BTPP-TPT CMPs.

S16. Dye adsorption Experiments

Rhodamine B (RhB), and methylene blue (MB) dyes were selected to study the efficiency of the BTPP-TPA and BTPP-TPT CMPs to remove them from water. In a typical experiment, polymer (6 mg) was added to an aqueous solution of the dye (RhB, 10 mL) and (MB, 10 mL) in a glass vial and then the mixture was stirred (for 0, 15, 30, 45, 60, 75, 90 min.). The initial concentration of both RhB and MB dyes are 10 mg L^{-1} on to the BTPP-TPA CMP and 20 mg L^{-1} on to the BTPP-TPT CMP. The supernatant was then isolated through centrifugation (6000 rpm, 10 min). The UV–Vis spectrum of the isolated supernatant was measured.

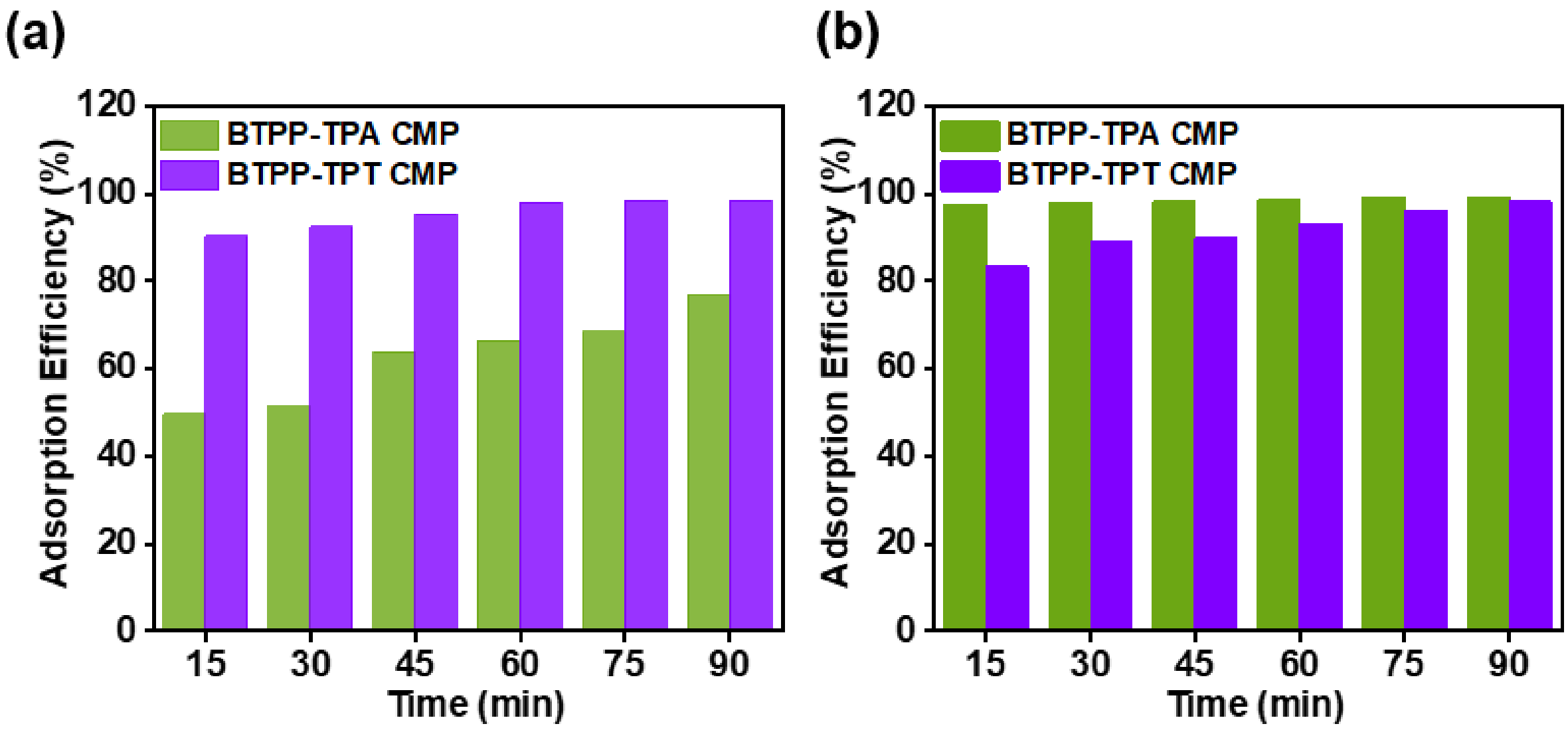

Figure S16. Adsorption efficiencies of BTPP-TPA and BTPP-TPT CMPs on (a) RhB and (b) MB organic dyes from an aqueous solution.

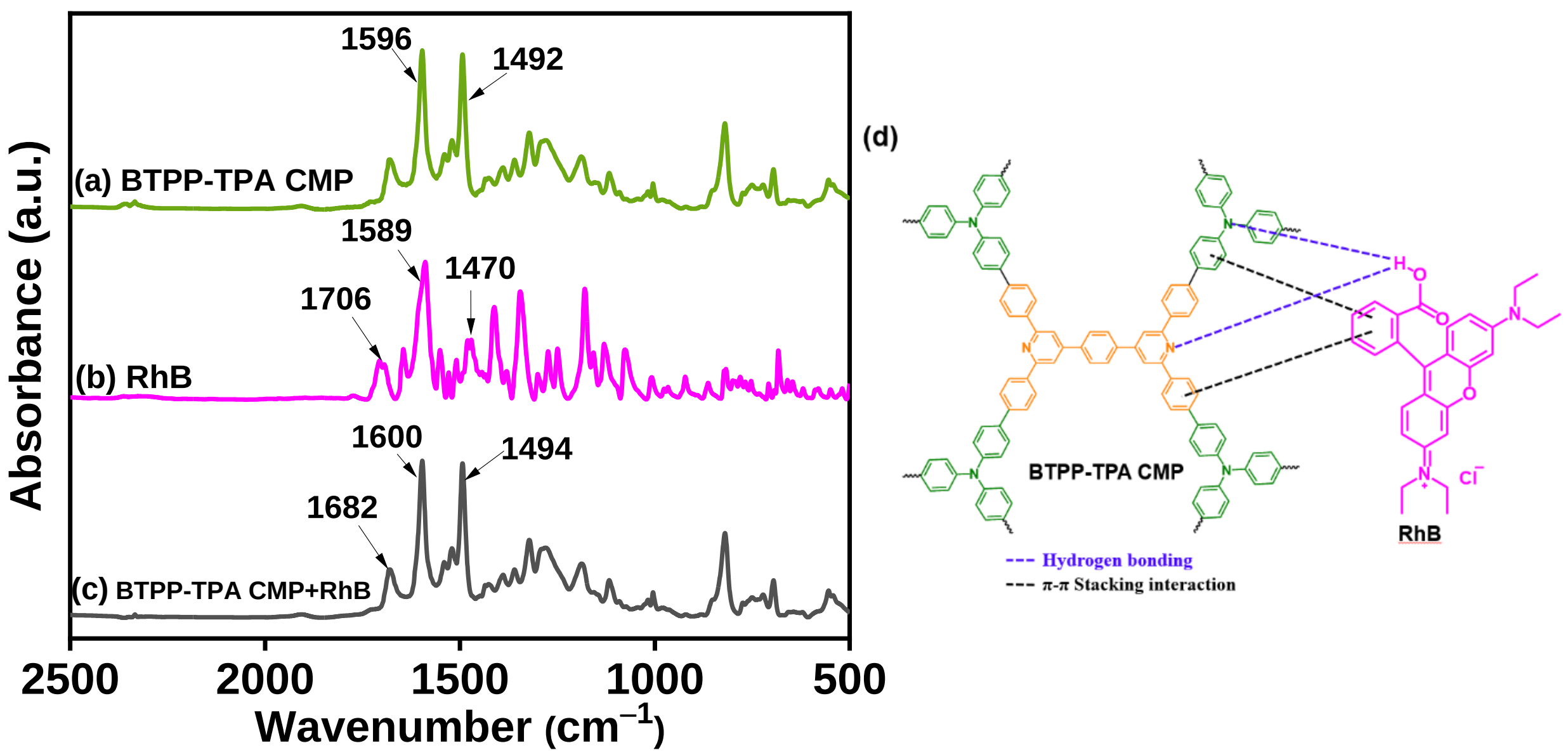

Figure S17. FT-IR spectra of (a) BTPP-TPA CMP, (b) Rhodamine B (RhB), and (c) BTPP-TPA CMP after RhB adsorption. (d) Adsorption mechanism of RhB on BTPP-TPA CMP.

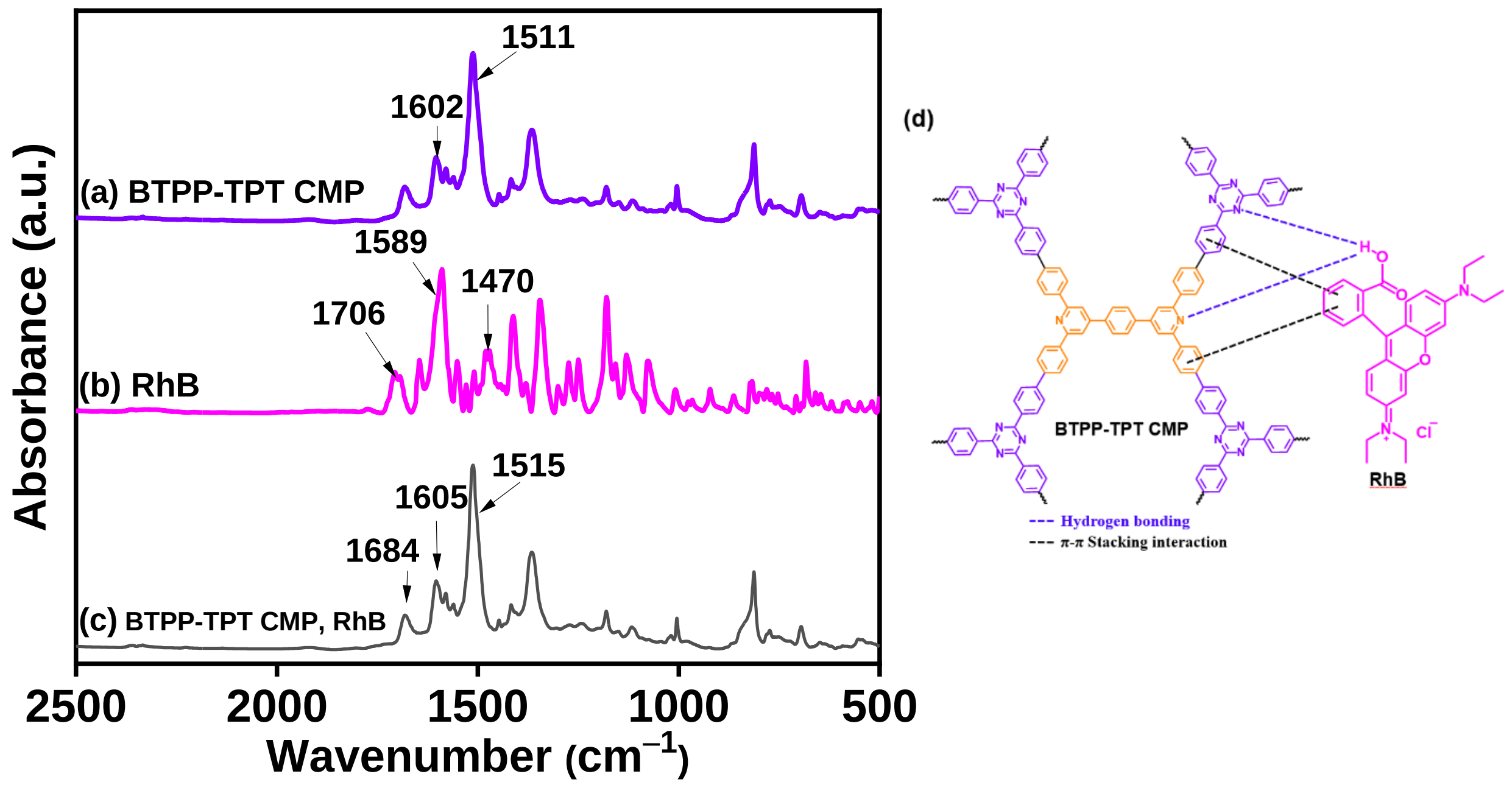

Figure S18. FT-IR spectra of (a) BTPP-TPT CMP, (b) Rhodamine B (RhB), and (c) BTPP-TPT CMP after RhB adsorption. (d) Adsorption mechanism of RhB on BTPP-TPT CMP.

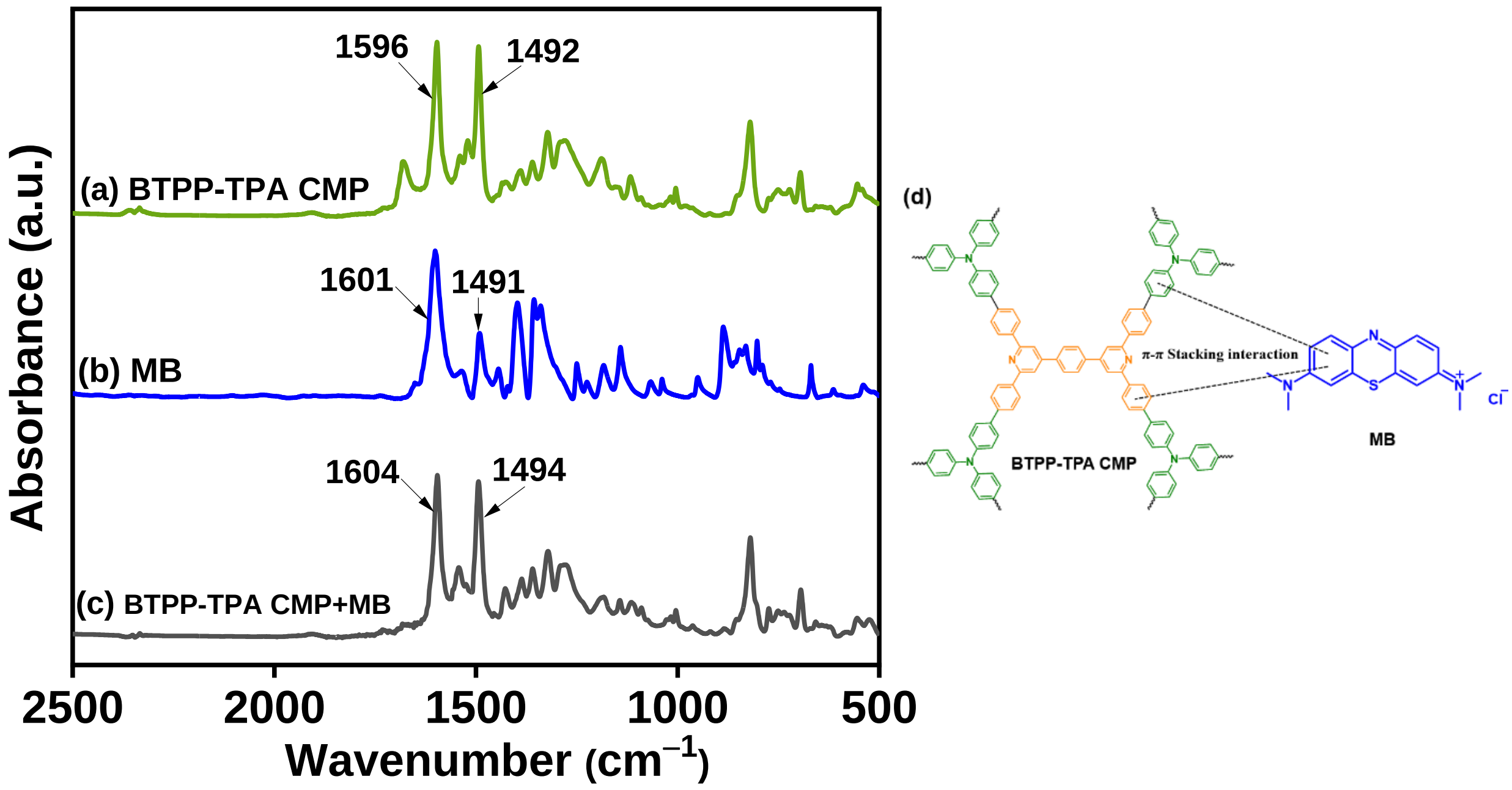

Figure S19. FT-IR spectra of (a) BTPP-TPA CMP, (b) Methylene Blue (MB), and (c) BTPP-TPA CMP after MB adsorption. (d) Adsorption mechanism of MB on BTPP-TPA CMP.

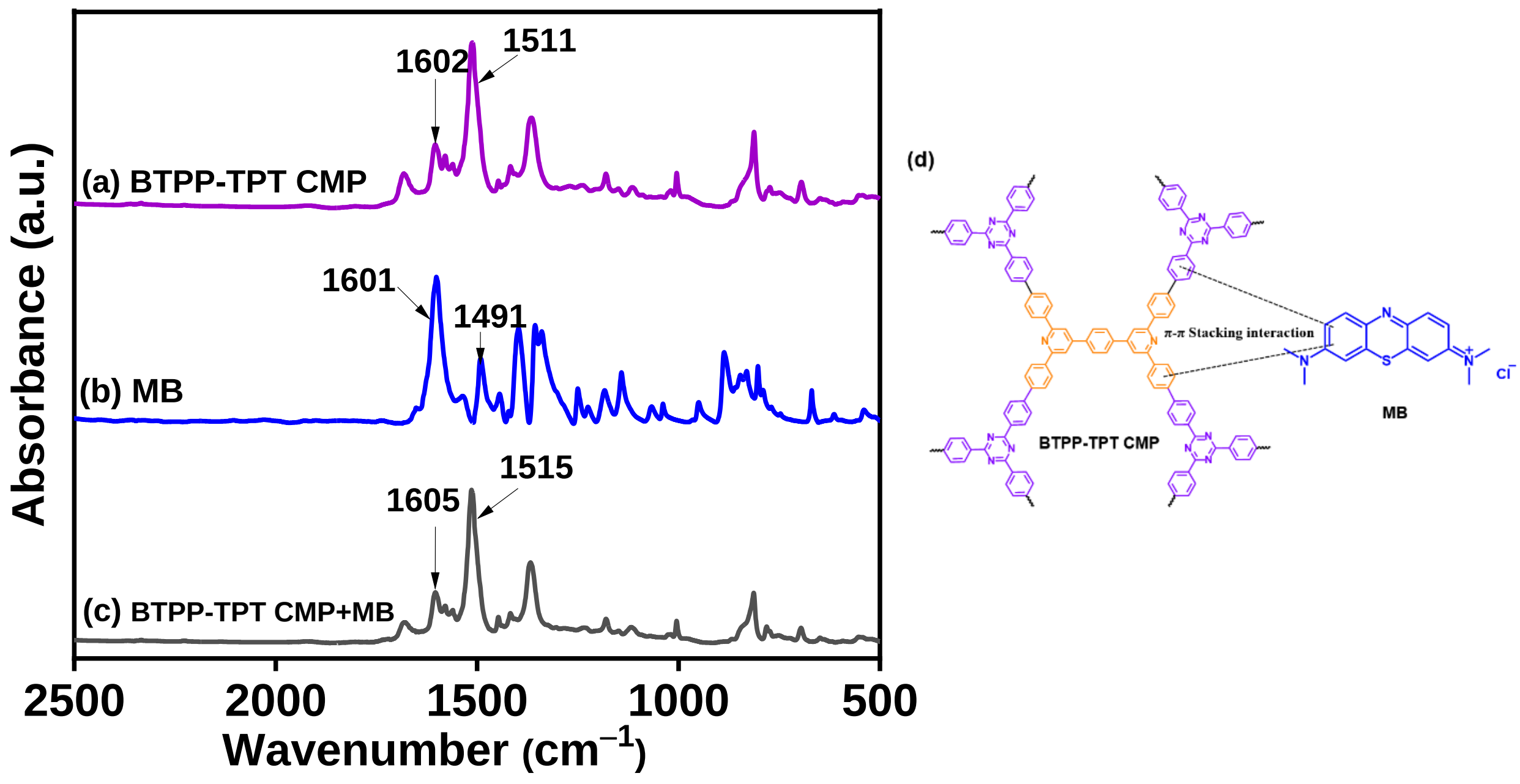

Figure S20. FT-IR spectra of (a) BTPP-TPT CMP, (b) Methylene Blue (MB), and (c) BTPP-TPT CMP after MB adsorption. (d) Adsorption mechanism of MB on BTPP-TPT CMP.

S17. Photodegradation

Photocatalytic degradation experiments were performed under visible-light irradiation at ambient temperature and atmospheric pressure in air, without N_2 or Ar purging. For the blank experiment, 60 mL of RhB or MB aqueous solution (20mg L^{-1}) was irradiated with visible light for 4 h in the absence of BTPP-TPA or BTPP-TPT CMPs. The distance between the liquid surface and the optical filter/light source was maintained at 8 cm.

In a typical photodegradation experiment, 6 mg of BTPP-TPA or BTPP-TPT CMP was added to 60 mL of RhB or MB aqueous solution (20mg L^{-1}) in a 100 mL glass beaker. The suspension was magnetically stirred at 460 rpm in the dark for 1 h to establish adsorption-desorption equilibrium between the dye molecules and the CMP catalyst. After this dark-equilibrium step, the suspension was exposed to visible-light irradiation for 4 h. At 30 min intervals, 3 mL aliquots of the reaction mixture were collected using a pipette. The catalyst was removed by centrifugation at 6000 rpm for 10 min, and the UV–Vis absorption spectrum of the isolated supernatant was recorded to monitor the degradation process. The apparent photodegradation efficiency was calculated using the following equation:

$$\text{Degradation efficiency (\%)} = [(C_0 - C_t) / C_0] \times 100$$

where C_0 is the dye concentration in the liquid phase after the dark adsorption-desorption equilibrium step, corresponding to the concentration at the beginning of irradiation, and C_t is the dye concentration in the liquid phase after irradiation for time t.

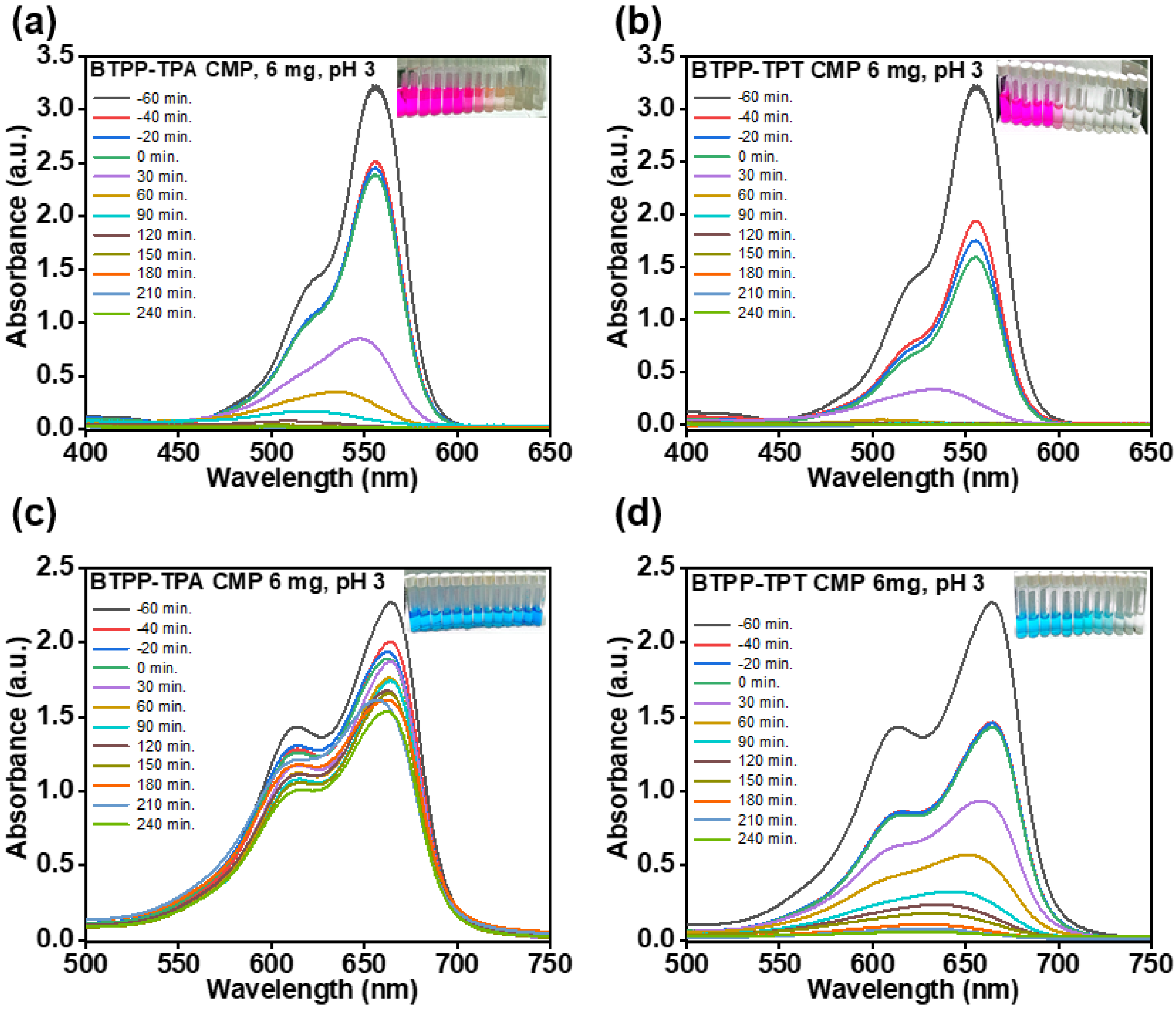

Figure S21. (a–d) UV–Vis spectroscopy of photocatalytic degradation of (a,b) RhB and (c,d) MB dyes at various time intervals after the addition of (a,c) BTPP-TPA and (b,d) BTPP-TPT CMPs.

Table S10. Photodegradation performance of RhB on the BTPP-TPA and BTPP-TPT CMPs, compared with other reported materials.

Catalyst	Catalyst amount	Dye RhB solution	Degradation time (min)	Degradation efficiency (%)	Ref.
$Cu_{0.5}Mg_{0.5}Fe_2O_4$–TiO_2	20 mg	10 mg L^{-1}, 20mL	180	98.4	J.Hazard. Mater. **420** (2021) 126636.
BC-TT CMP	7 mg	20 mg L^{-1}, 56mL	135	100	MATER. TODAY SUSTAIN. **23** (2023) 100429
BF-TT CMP	7 mg	10 mg L^{-1}, 56mL	135	80	MATER. TODAY SUSTAIN. **23** (2023) 100429
BIPE-TT CMP	7 mg	100 mg L^{-1}, 56mL	135	75	MATER. TODAY SUSTAIN. **23** (2023) 100429
TPA-TAZ HPP	3 mg	75 mg L^{-1}, 60mL	330	88	Colloids Surf. A, Physicochem. Eng. Aspects **722** (2025) 137239
Py-CMP-1	6 mg	20 mg L^{-1}, 60 mL	90	16.22	Polym. Chem. **13** (2022) 5300-5308.
Py-CMP-2	6 mg	20 mg L^{-1}, 60 mL	90	95.77	Polym. Chem. **13** (2022) 5300-5308.
MgAl-LDH	Thin-film	20 mg L^{-1}, 40 mL	35	99	J. Mater. Chem. A **12** (2024) 3411-3433.
LDH–EDTA	Thin-film	20 mg L^{-1}, 40 mL	30	99.4	J. Mater. Chem. A **12** (2024) 3411-3433.
LDH–EDTA–Zn	Thin-film	20 mg L^{-1}, 40 mL	25	99.7	J. Mater. Chem. A **12** (2024) 3411-3433.
AZO-TFP-BT-TFP COF	8 mg	25 mg L^{-1}, 60 mL	180	97.70 ± 0.51	Sep. Purif. Technol. **356** (2025) 129950
AZO-TFP-MBT-TFP COF	8 mg	25 mg L^{-1}, 60 mL	210	95.82 ± 0.61	Sep. Purif. Technol. **356** (2025) 129950
**BTPP-TPA CMP**	**6 mg**	**20 mg L^{-1}, 60 mL**	**120**	**99.08**	**This work**
**BTPP-TPT CMP**	**6 mg**	**20 mg L^{-1}, 60 mL**	**60**	**99.31**	**This work**

Table S11. Photodegradation performance of MB on the BTPP-TPA and BTPP-TPT CMPs, compared with other reported materials.

Catalyst	Catalyst amount	Dye MB solution	Degradation time (min)	Degradation efficiency (%)	Ref.
TPA-TAZ HPP	3 mg	75 mg L^{-1}, 60mL	330	96	Colloids Surf. A, Physicochem. Eng. Aspects **722** (2025) 137239
BC-TT CMP	7 mg	20 mg L^{-1}, 56mL	135	100	MATER. TODAY SUSTAIN. **23** (2023) 100429
BF-TT CMP	7 mg	10 mg L^{-1}, 56mL	135	66	MATER. TODAY SUSTAIN. **23** (2023) 100429
BIPE-TT CMP	7 mg	100 mg L^{-1}, 56mL	135	66	MATER. TODAY SUSTAIN. **23** (2023) 100429
N-doped MCMs@TiO_2	50 mg	40 mg L^{-1}, 40 mL	~120	95	Chem. Eng. J. **411** (2021) 128615.
PVDF/PVP-Cu_2O	10 mg	10 mg L^{-1}, 50 mL	120	93.6	Langmuir **40** (2024) 1447–1460.
NZGZA-1	150 mg	10 mg L^{-1}, 100 mL	180	87.08	J. Photochem. Photobiol. A **449** (2024) 115369
COFs quantum dots	20 mg	10 mg L^{-1}, 20 mL	~40	＞99.97	J. Mater. Sci. Mater. **32** (2021) 23018-23029
TTB-TTA	20 mg	10 mg L^{-1}, 100 mL	30	50.9	Micropor. Mesopor. Mat. **341** (2022) 112107
FeIII 6.25-TA@TTB-TTA	20 mg	10 mg L^{-1}, 100 mL	30	94.9	Micropor. Mesopor. Mat. **341** (2022) 112107
AZO-TFP-BT-TFP COF	8 mg	12.5 mg L^{-1},60 mL	180	95.72 ± 0.59	Sep. Purif. Technol. **356** (2025) 129950
AZO-TFP-MBT-TFP COF	8 mg	12.5 mg L^{-1},60 mL	240	61.17 ± 2.96	Sep. Purif. Technol. **356** (2025) 129950
**BTPP-TPA CMP**	**6 mg**	**20 mg L^{-1}, 60 mL**	**240**	**19.05**	**This work**
**BTPP-TPT CMP**	**6 mg**	**20 mg L^{-1}, 60 mL**	**180**	**95.8**	**This work**

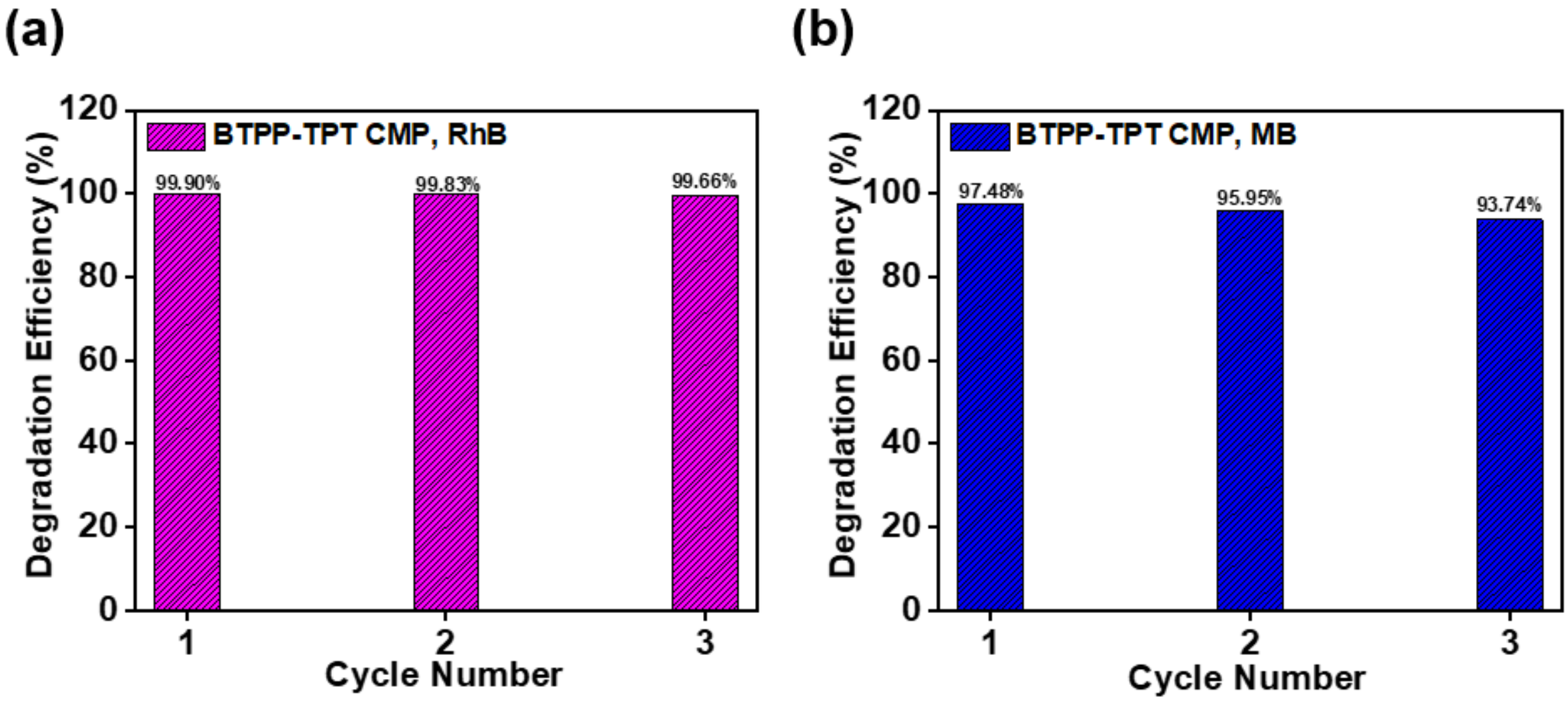

Figure S22. Cycling the photocatalytic degradation of (a) RhB and (b) MB over BTPP-TPT CMP in water. Each experiment was repeated three times.

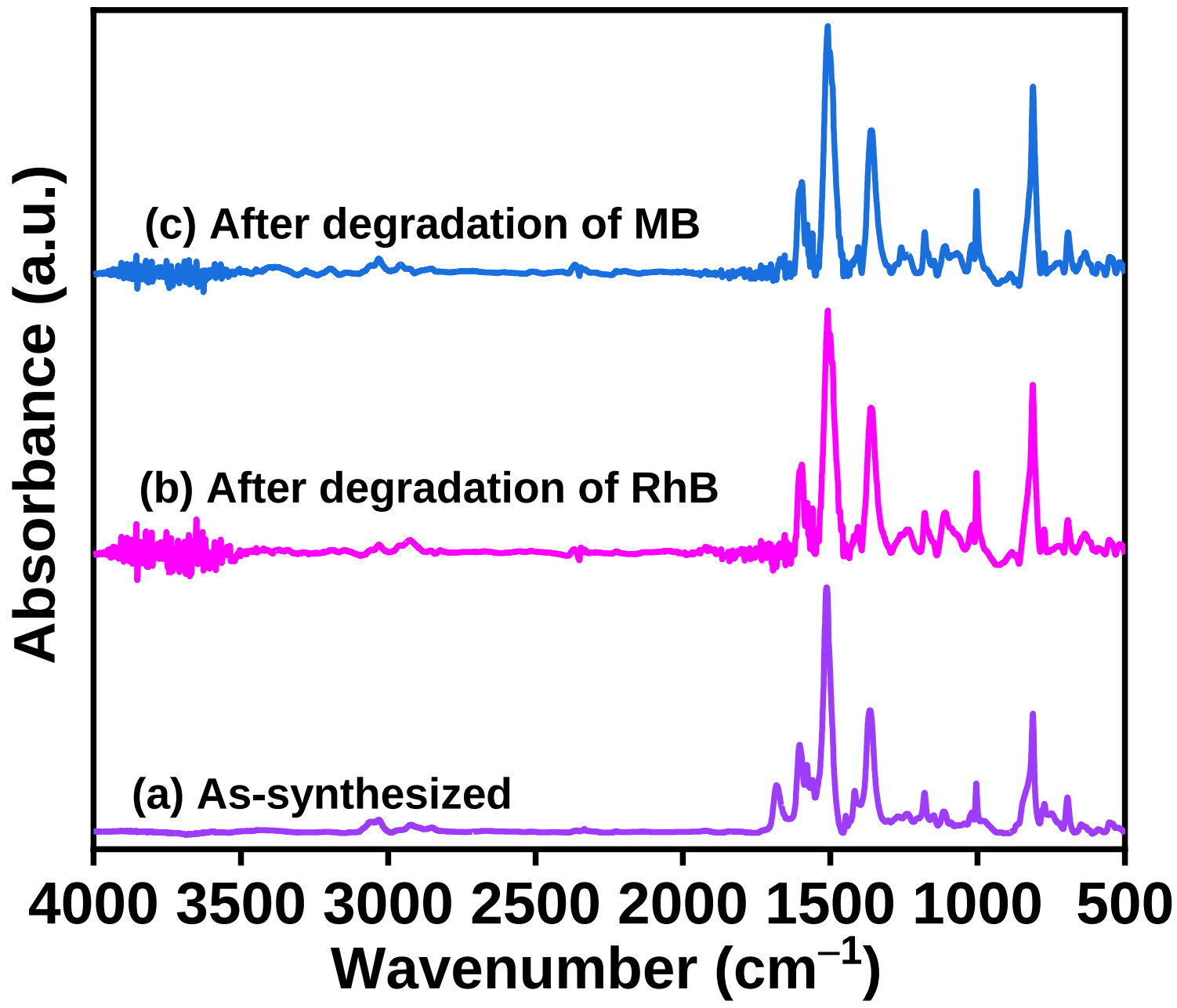

Figure S23. FTIR spectra of BTPP-TPT CMP before and after cycling photocatalytic degradation of RhB and MB.

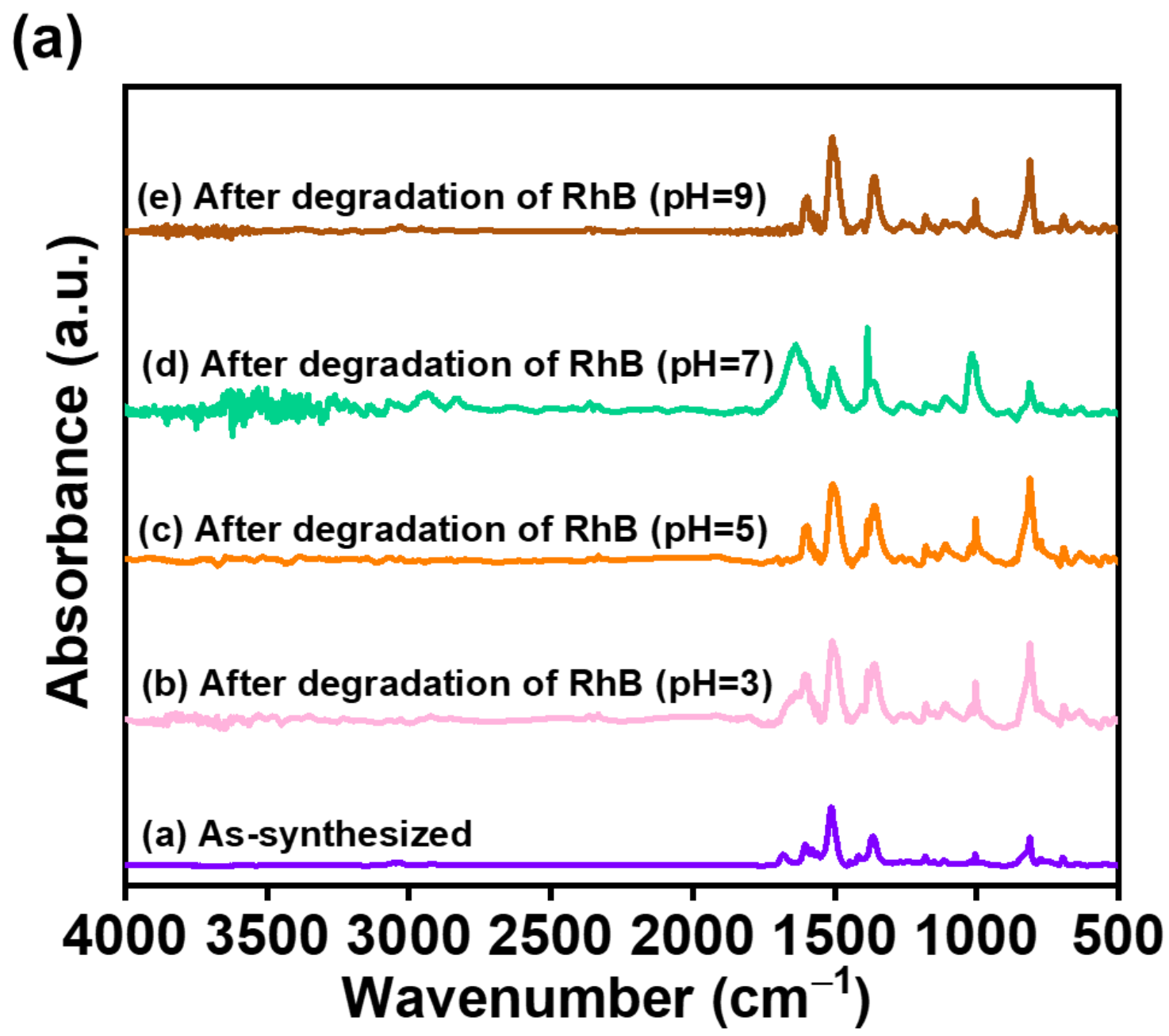

Figure S24. FTIR spectra of BTPP-TPT CMP before and after cycling photocatalytic degradation of RhB at different pH ranges.

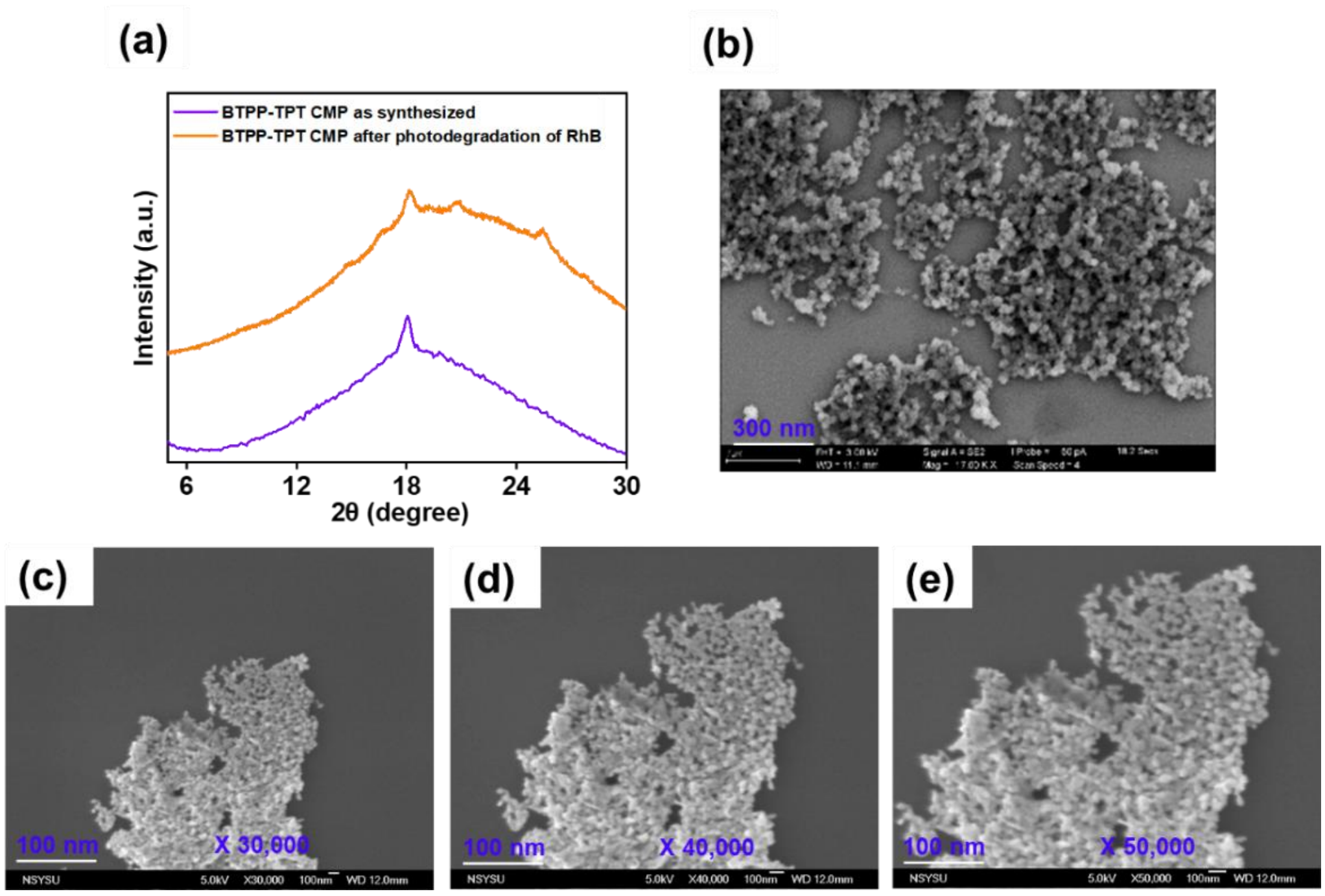

Figure S25. (a) XRD of BTPP-TPT CMP before and after photocatalytic degradation of RhB. (b-e) FE-SEM images of (b) the as-synthesized BTPP-TPT CMP, (c-e) BTPP-TPT CMP after cycling photocatalytic degradation of RhB (pH=3).

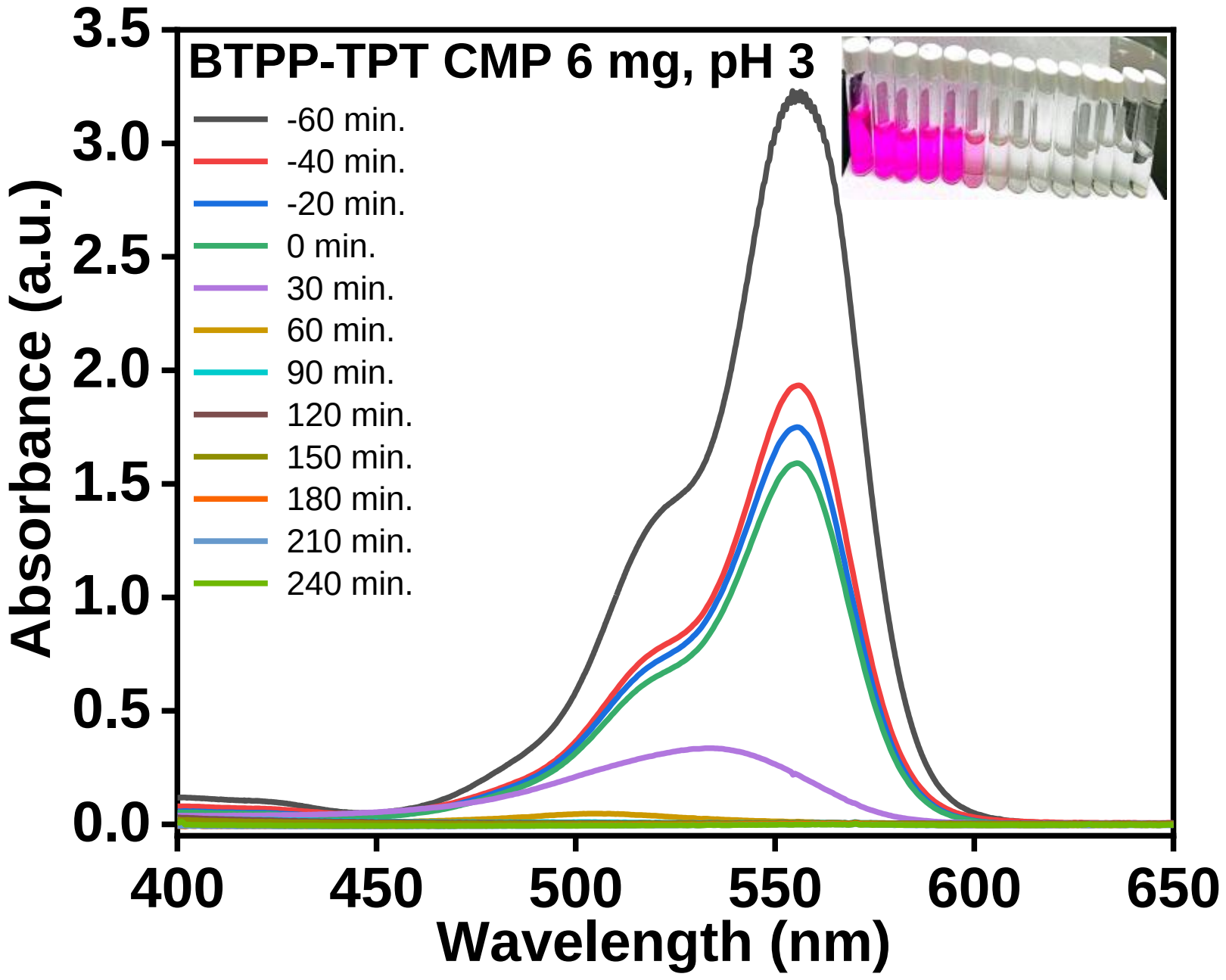

Figure S26. UV–Vis spectra of RhB solution (starting concentration: 20 mg L^{-1}, pH = 3) at different times after the addition of BTPP-TPT (6 mg).

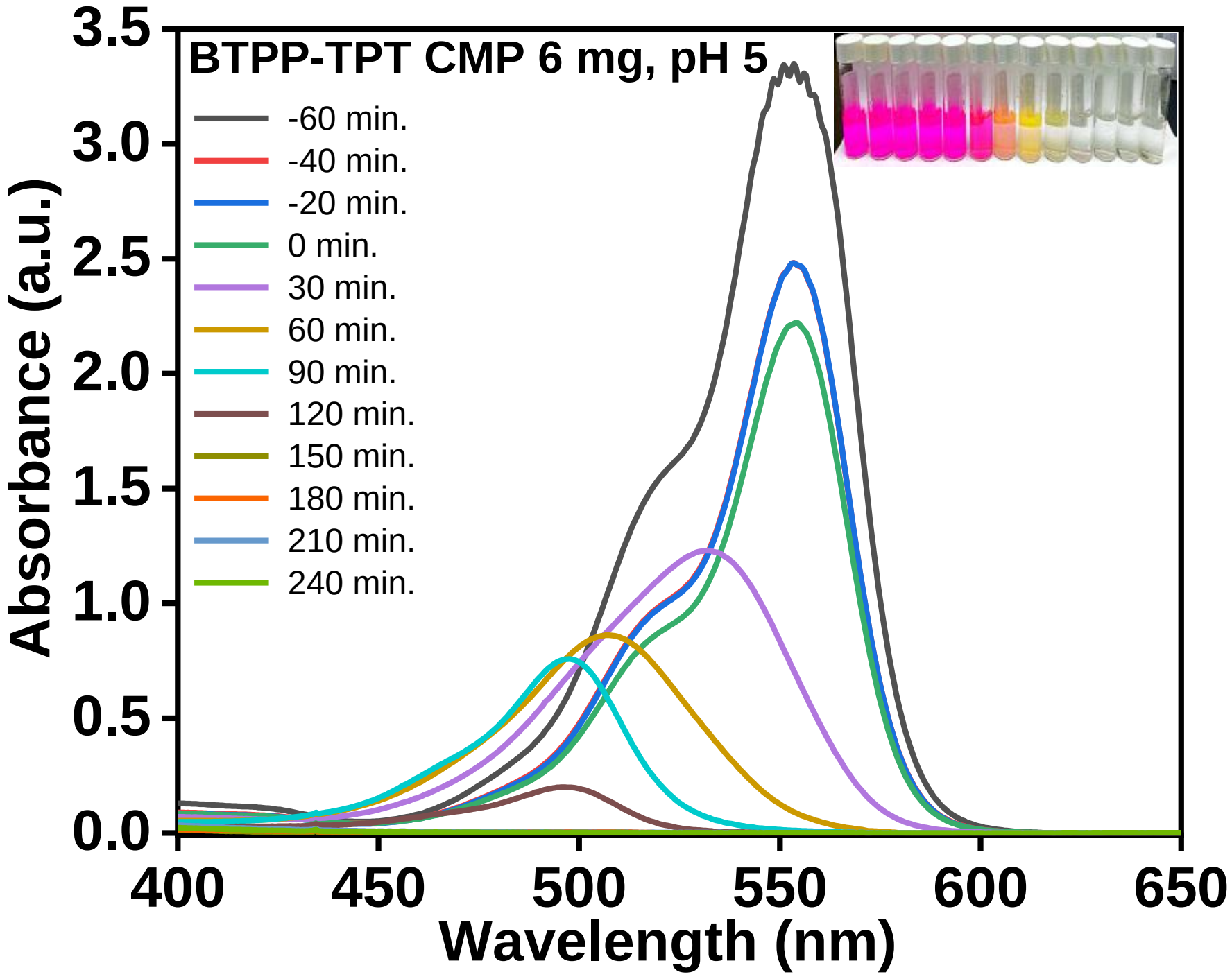

Figure S27. UV–Vis spectra of RhB solution (starting concentration: 20 mg L^{-1}, pH = 5) at different times after the addition of BTPP-TPT (6 mg).

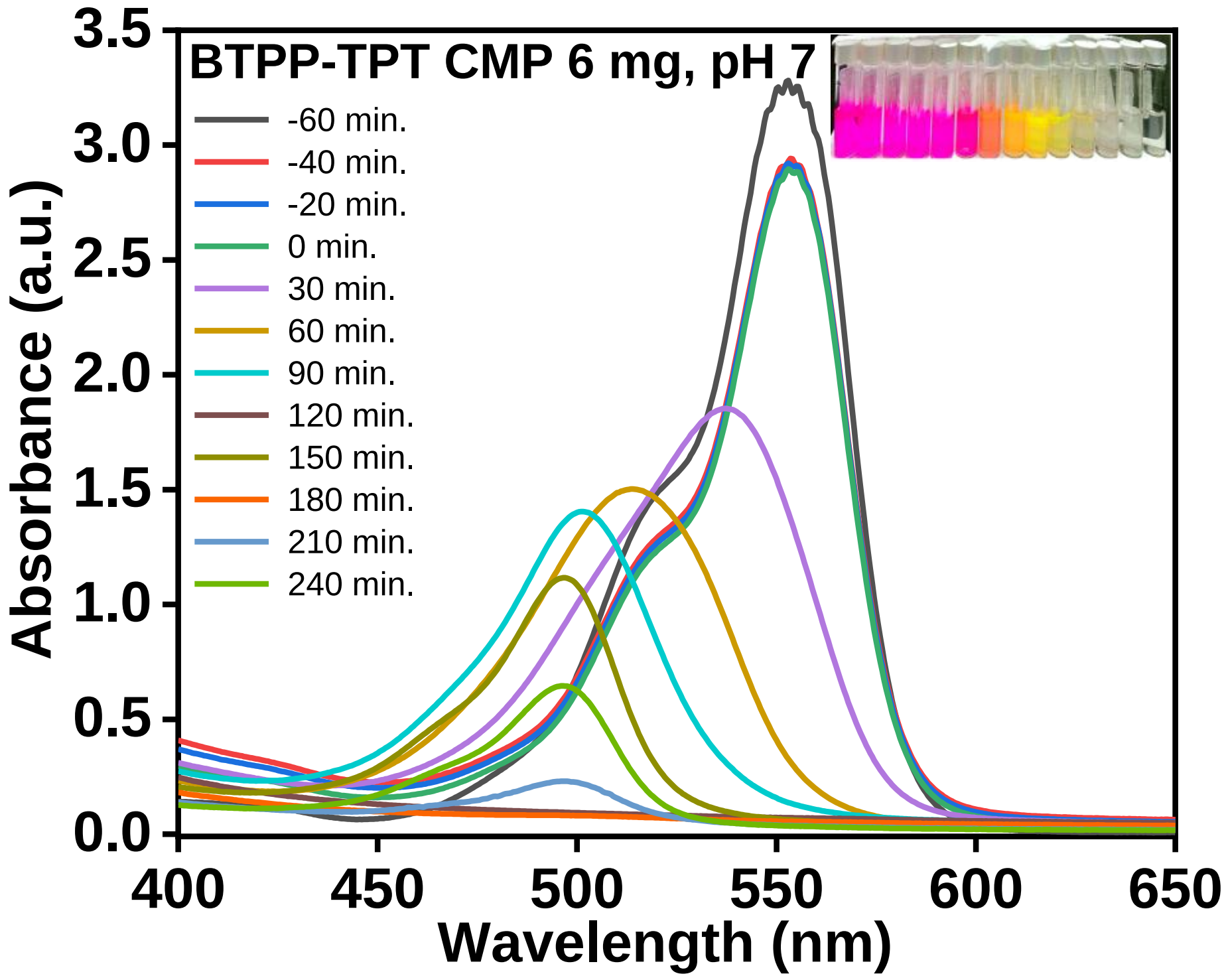

Figure S28. UV–Vis spectra of RhB solution (starting concentration: 20 mg L^{-1}, pH = 7) at different times after the addition of BTPP-TPT (6 mg).

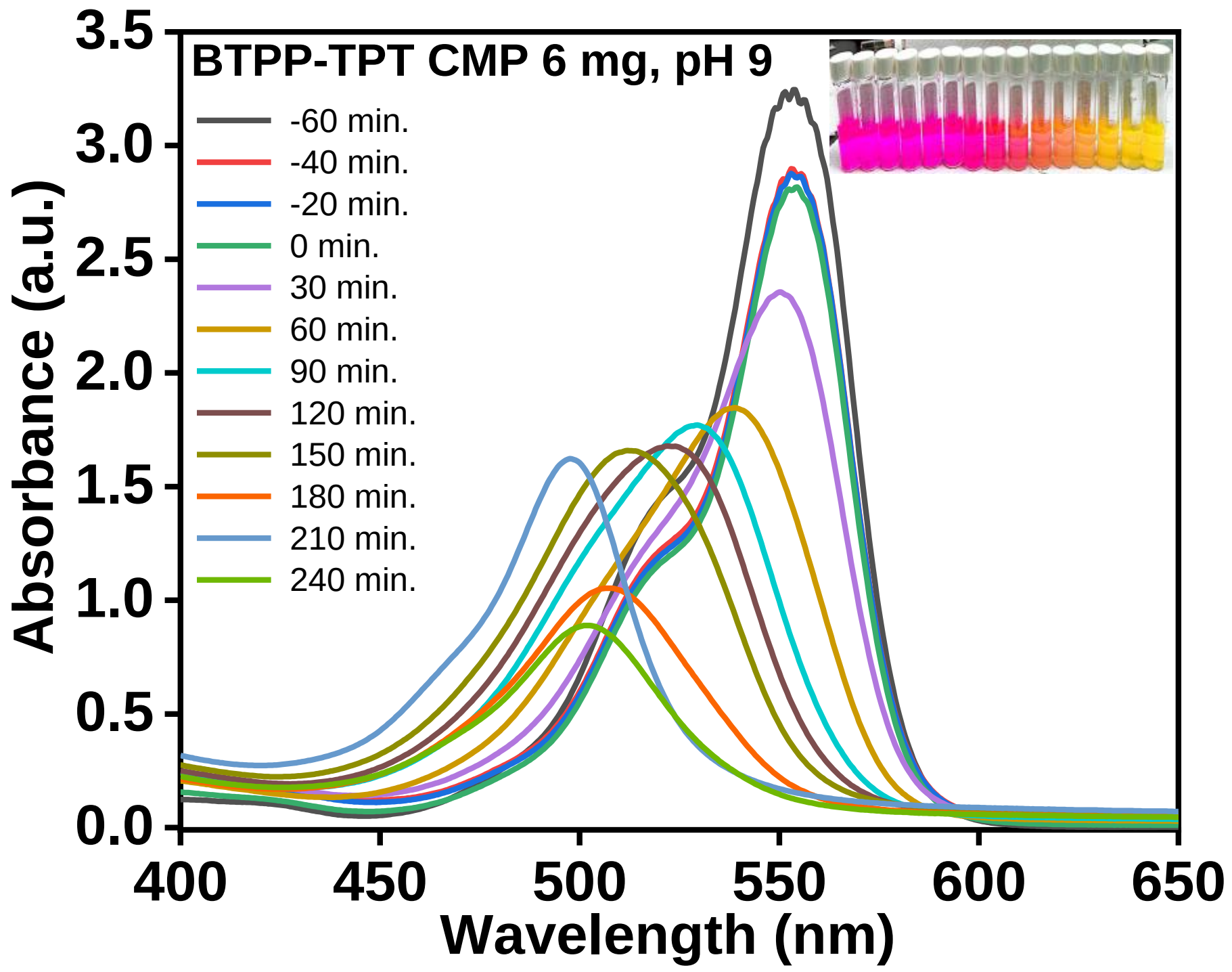

Figure S29. UV–Vis spectra of RhB solution (starting concentration: 20 mg L^{-1}, pH = 9) at different times after the addition of BTPP-TPT (6 mg).

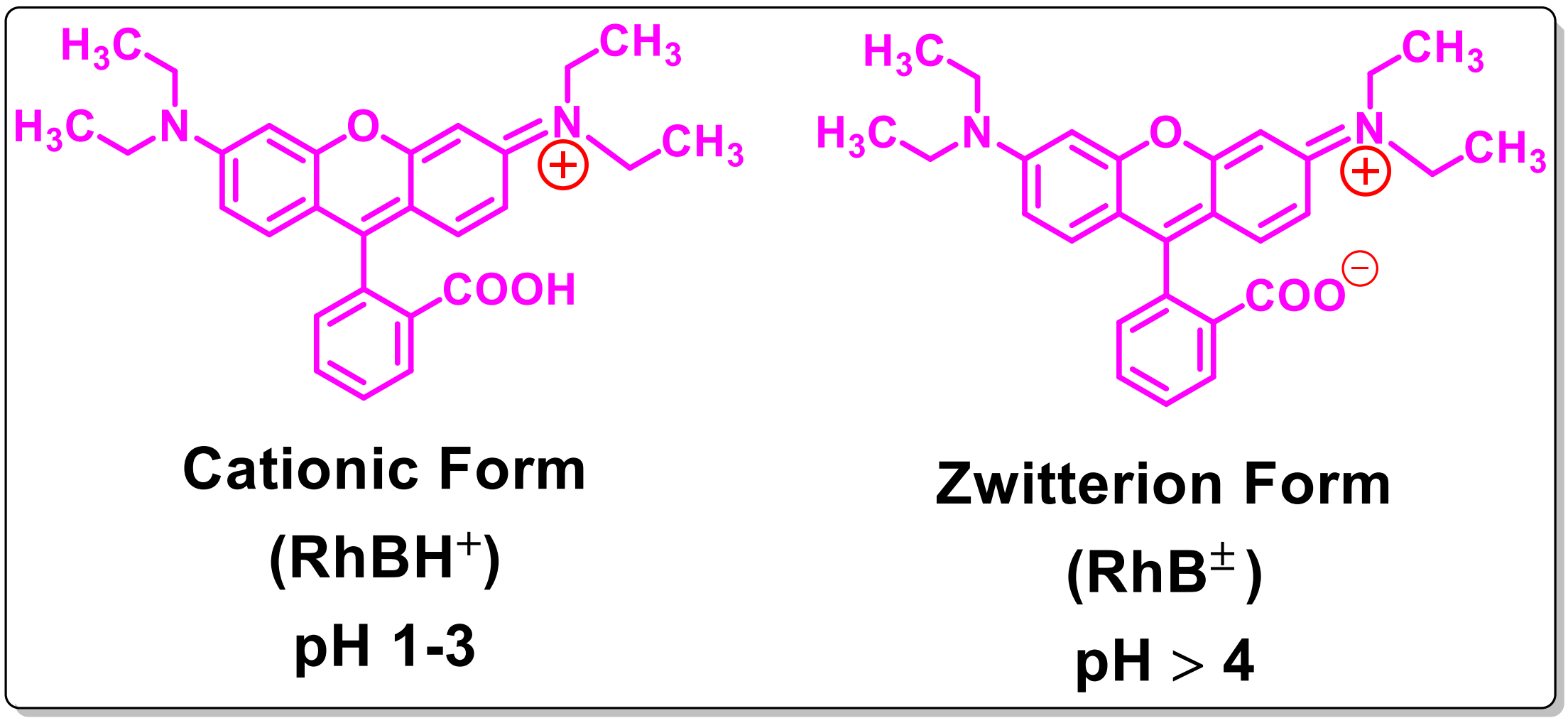

Figure S30. Molecular structure of RhB at varying pH values.

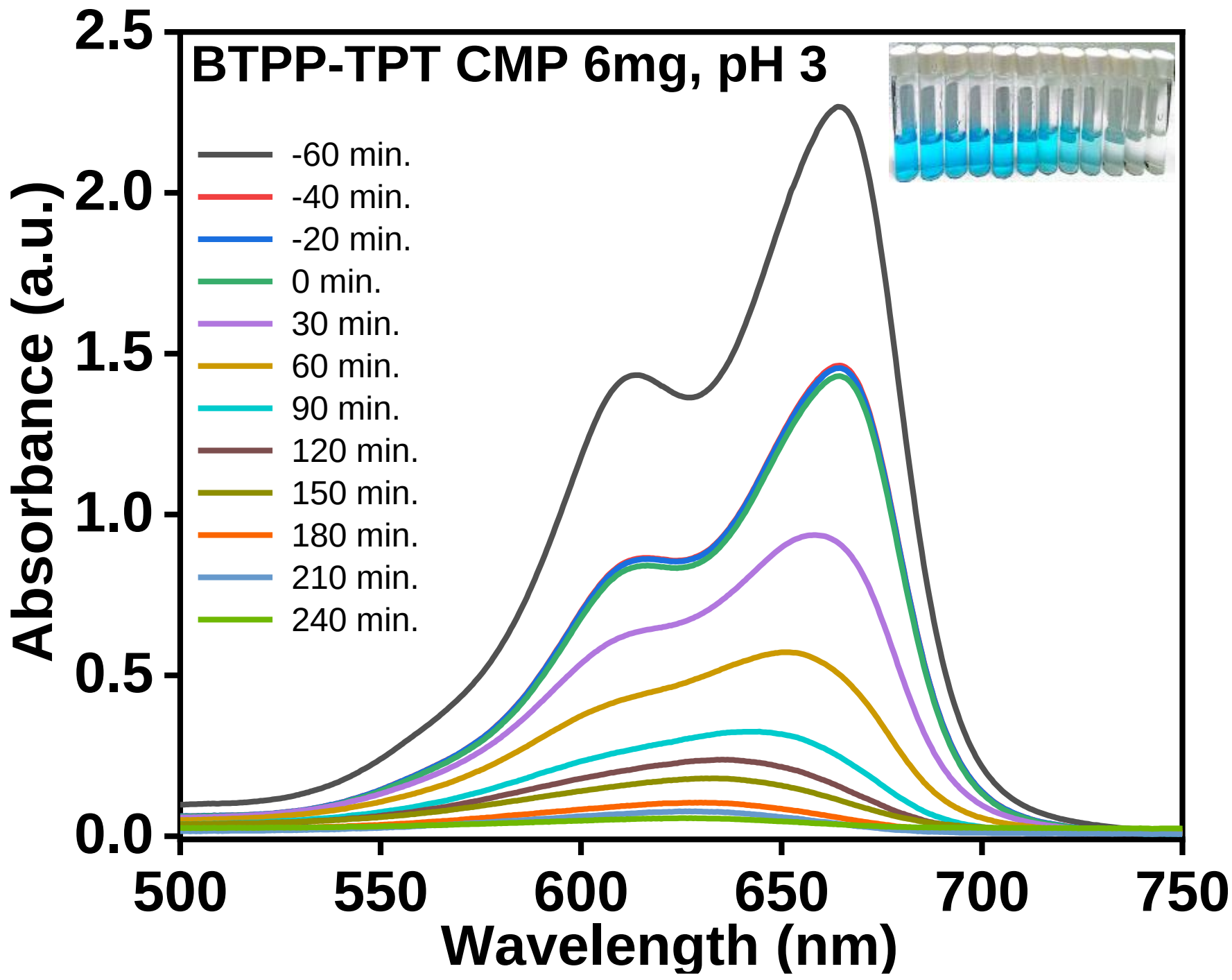

Figure S31. UV–Vis spectra of MB solution (starting concentration: 20 mg L^{-1}, pH = 3) at different times after the addition of BTPP-TPT (6 mg).

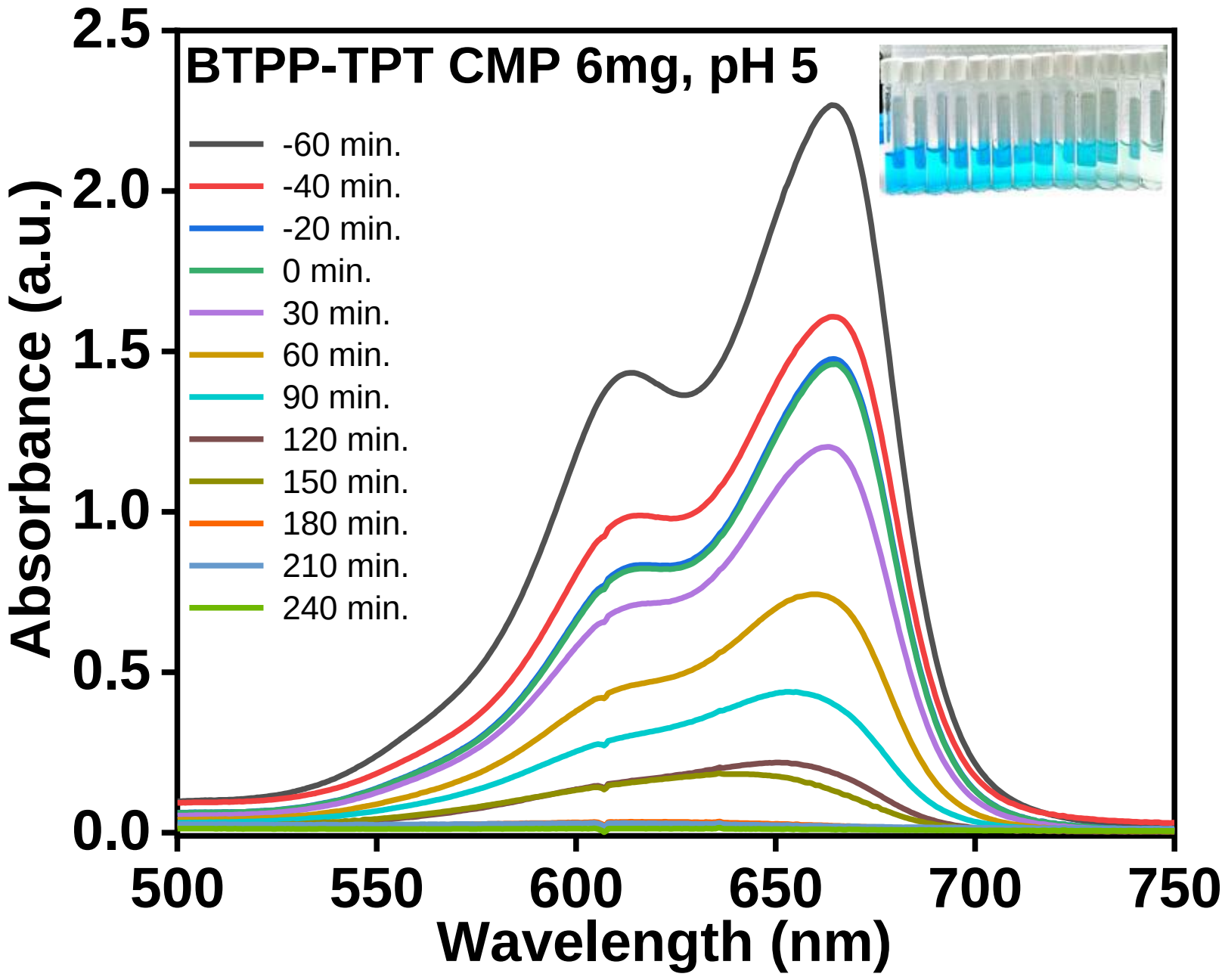

Figure S32. UV–Vis spectra of MB solution (starting concentration: 20 mg L^{-1}, pH = 5) at different times after the addition of BTPP-TPT (6 mg).

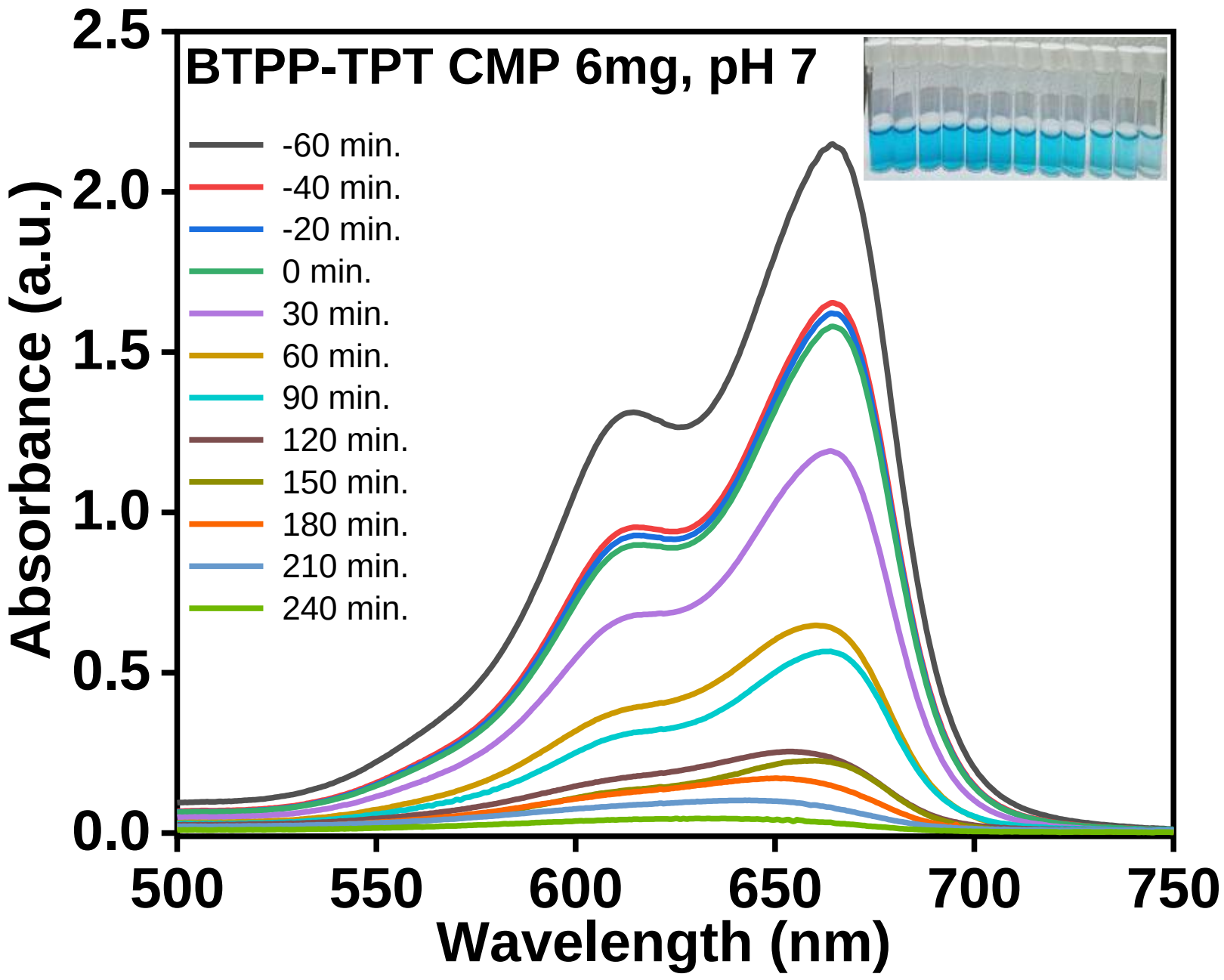

Figure S33. UV–Vis spectra of MB solution (starting concentration: 20 mg L^{-1}, pH = 7) at different times after the addition of BTPP-TPT (6 mg).

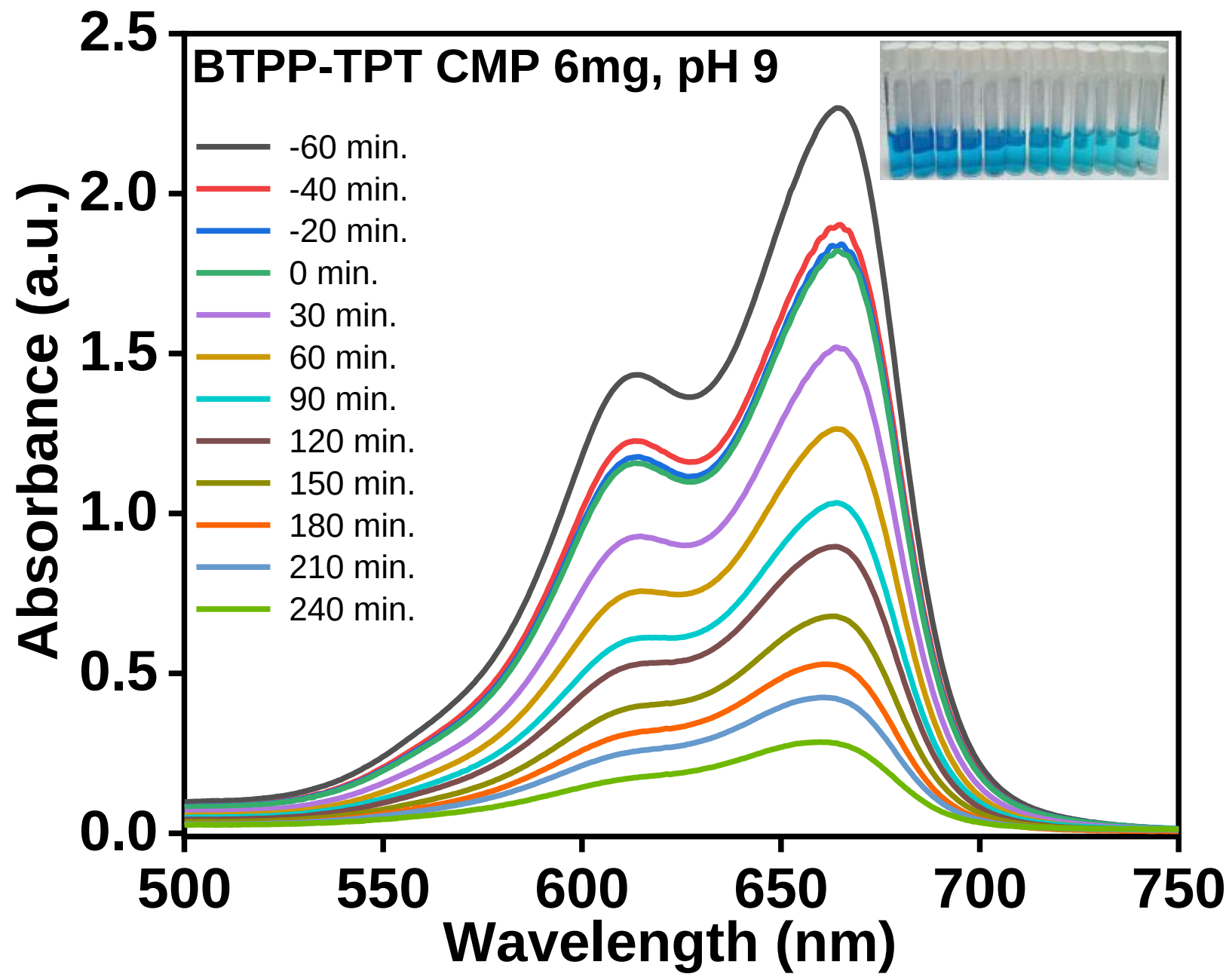

Figure S34. UV–Vis spectra of MB solution (starting concentration: 20 mg L^{-1}, pH = 9) at different times after the addition of BTPP-TPT (6 mg).

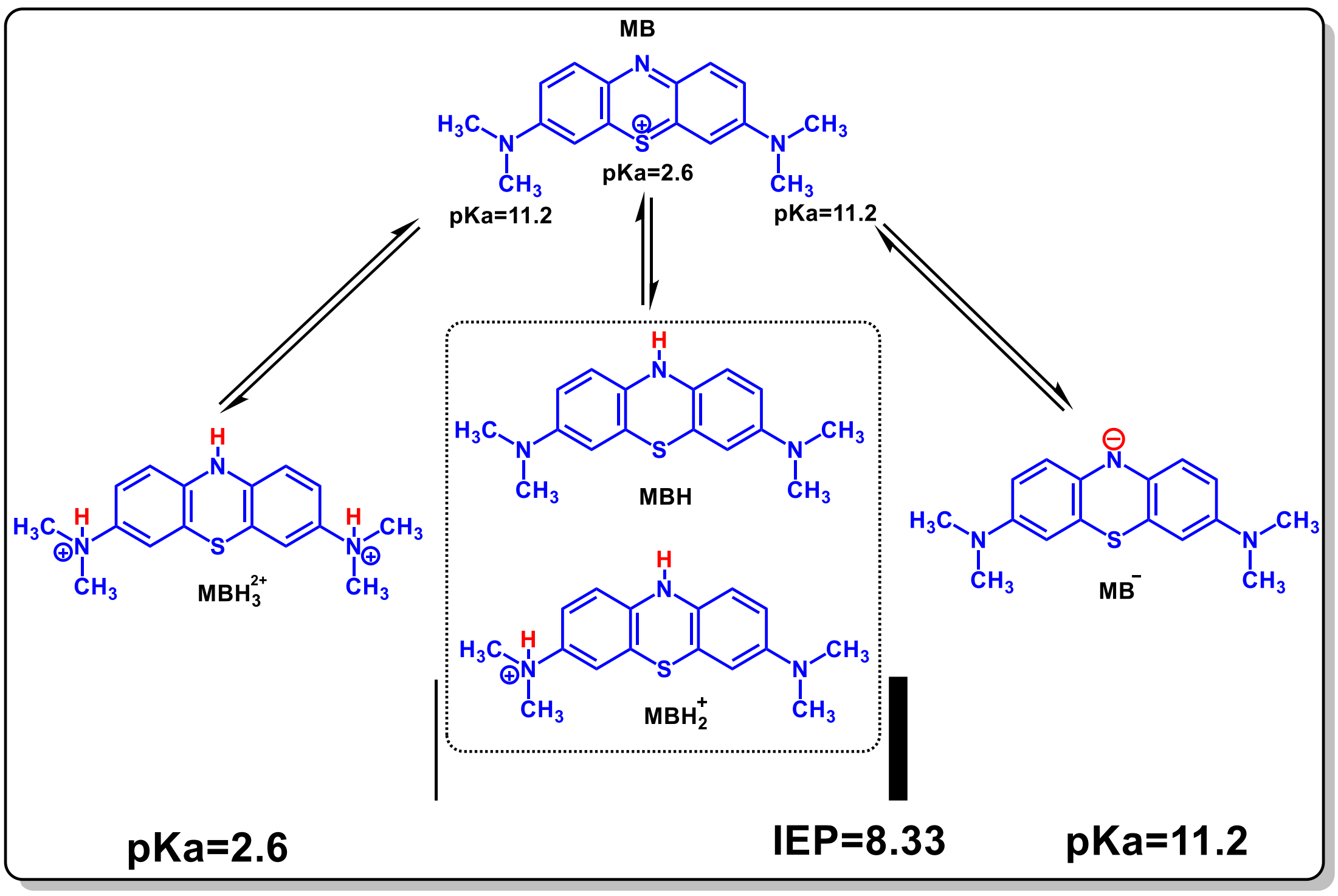

Figure S35. Molecular structure of MB at varying pH values.

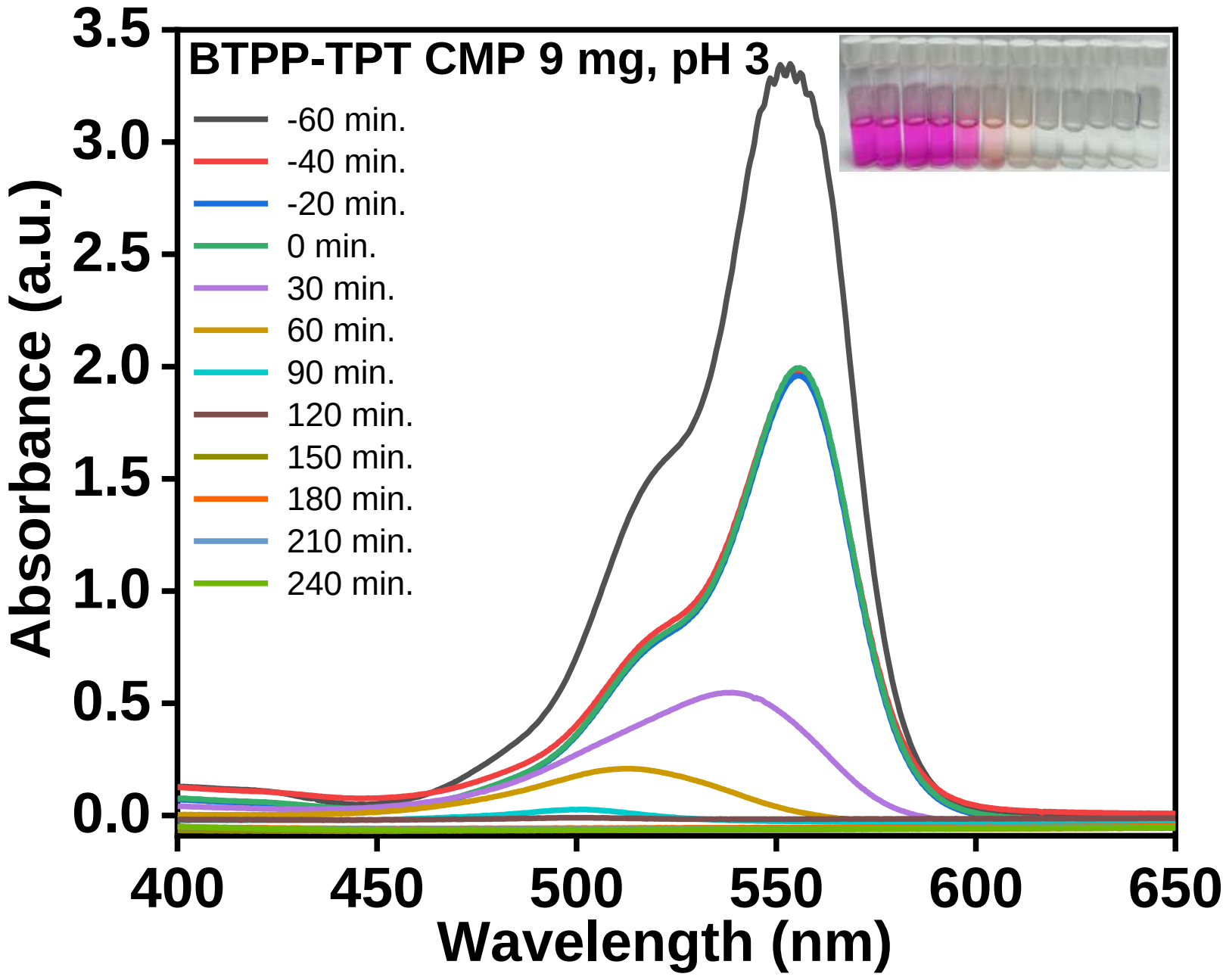

Figure S36. UV–Vis spectra of RhB solution (starting concentration: 20 mg L^{-1}, pH = 3) at different times after the addition of BTPP-TPT CMP (9 mg).

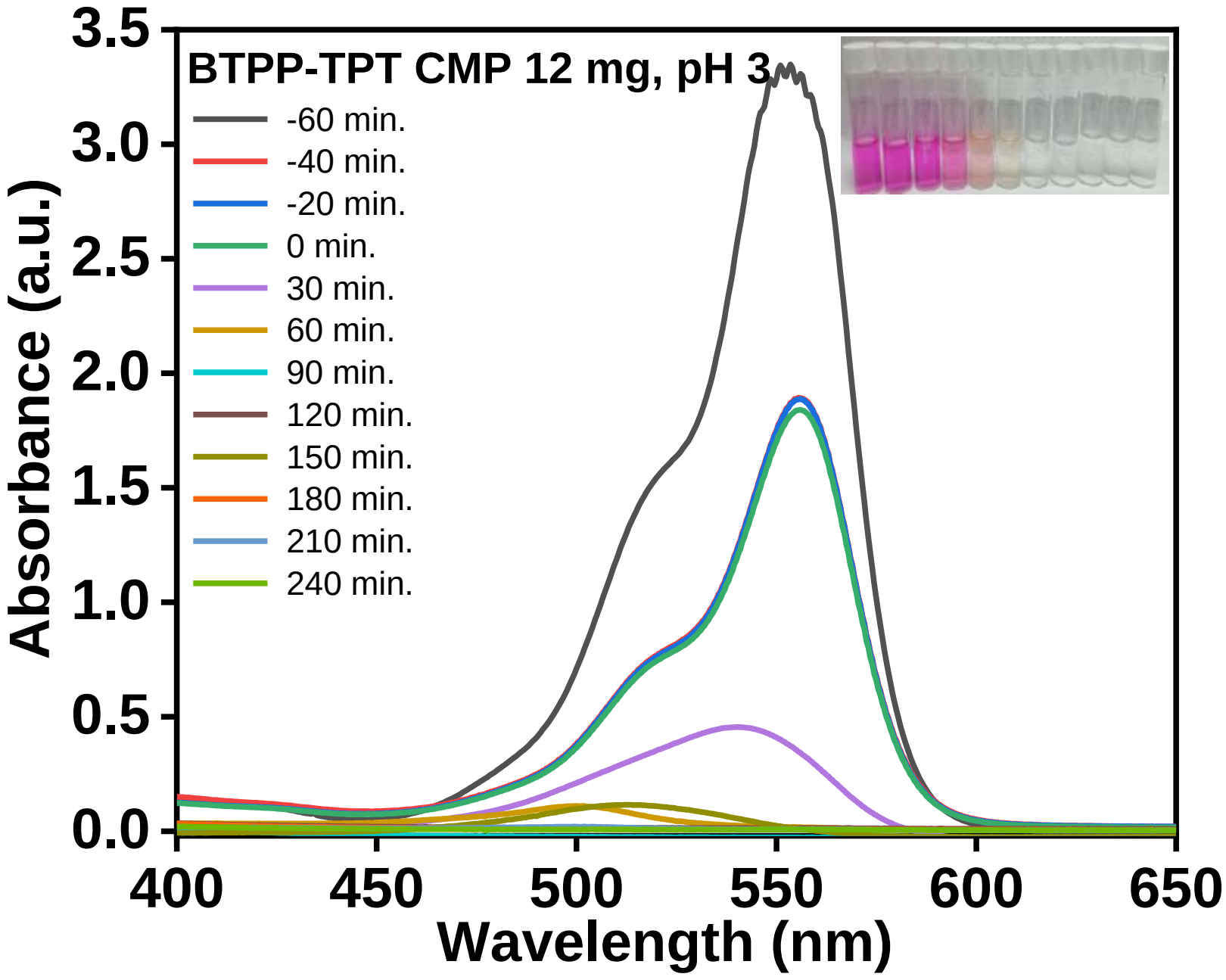

Figure S37. UV–Vis spectra of RhB solution (starting concentration: 20 mg L^{-1}, pH = 3) at different times after the addition of BTPP-TPT CMP (12 mg).

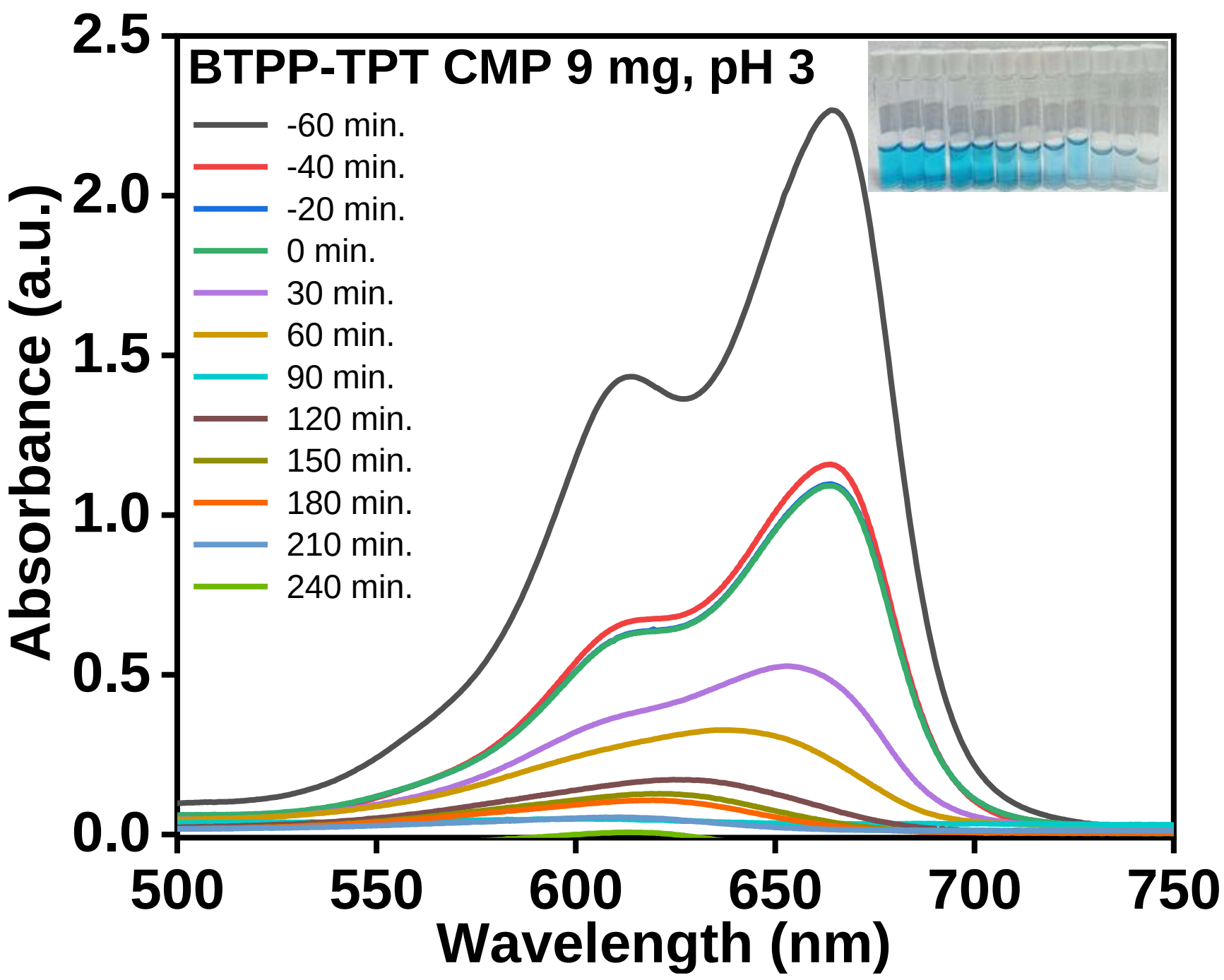

Figure S38. UV–Vis spectra of MB solution (starting concentration: 20 mg L^{-1}, pH = 3) at different times after the addition of BTPP-TPT CMP (9 mg).

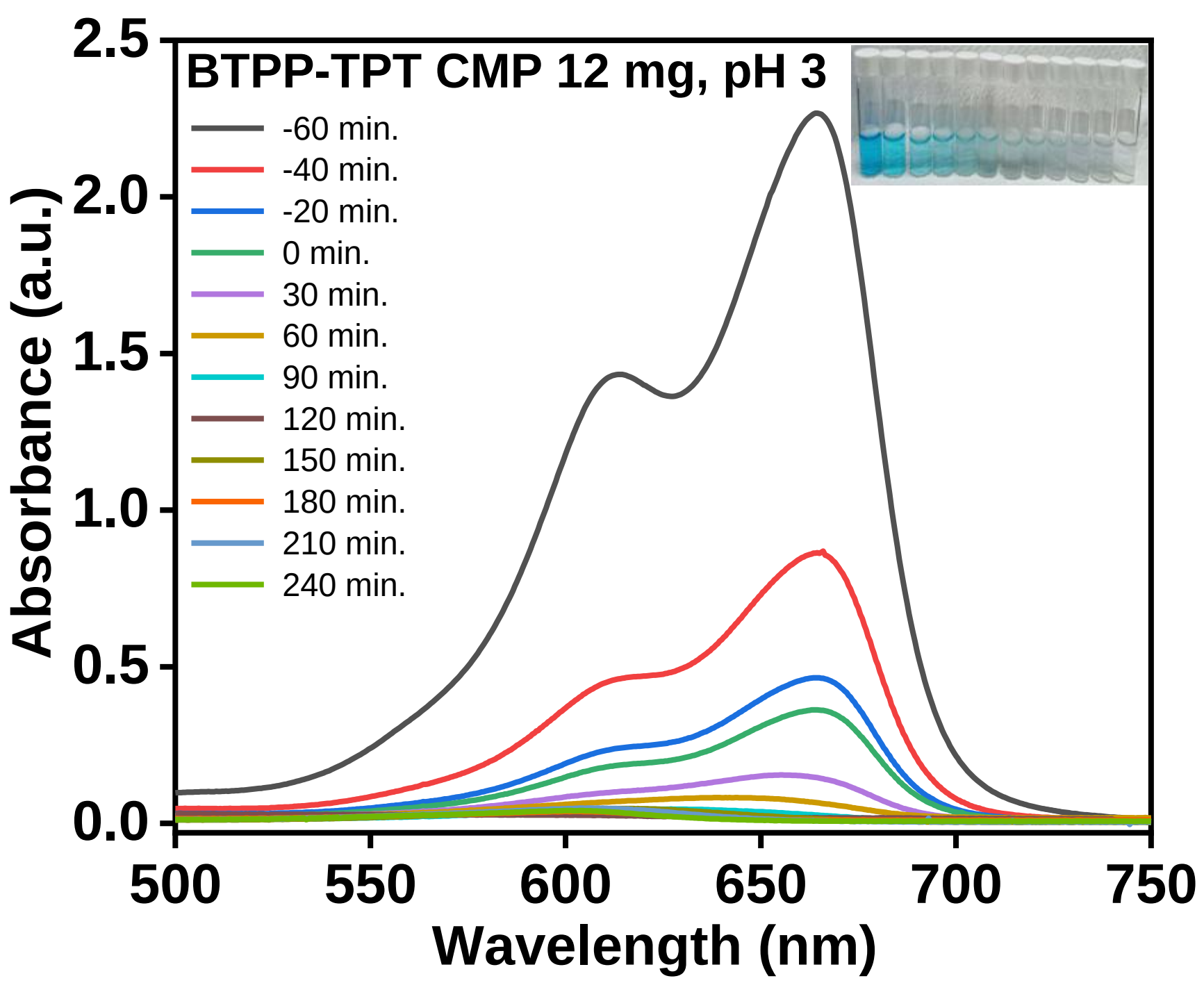

Figure S39. UV–Vis spectra of MB solution (starting concentration: 20 mg L^{-1}, pH = 3) at different times after the addition of BTPP-TPT CMP (12 mg).

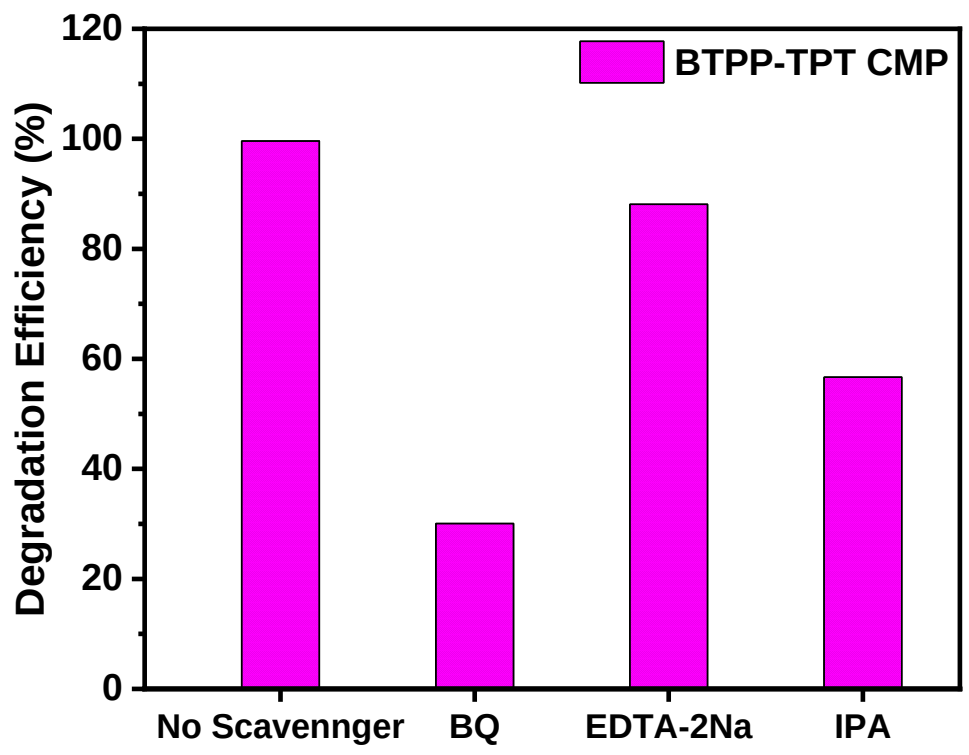

Figure S40. Effect of different scavengers on the photodegradation of RhB organic dye over BTPP–TPT CMP under visible light illumination for 240 mins.

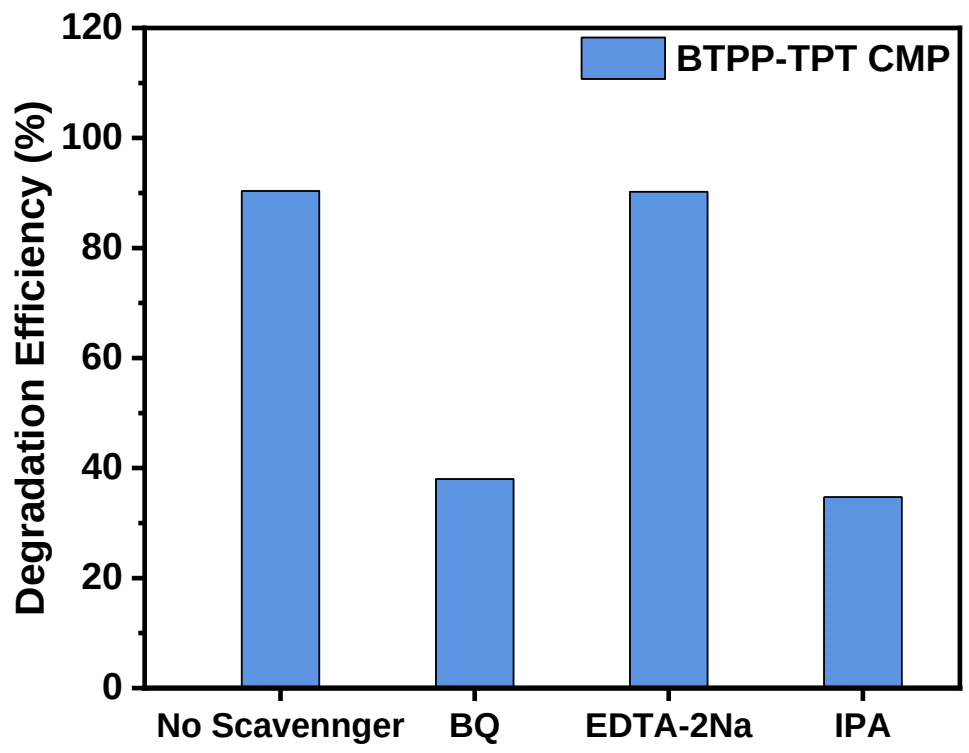

Figure S41. Effect of different scavengers onto photodegradation of MB organic dye over BTPP–TPT CMP under visible light illumination for 240 mins.

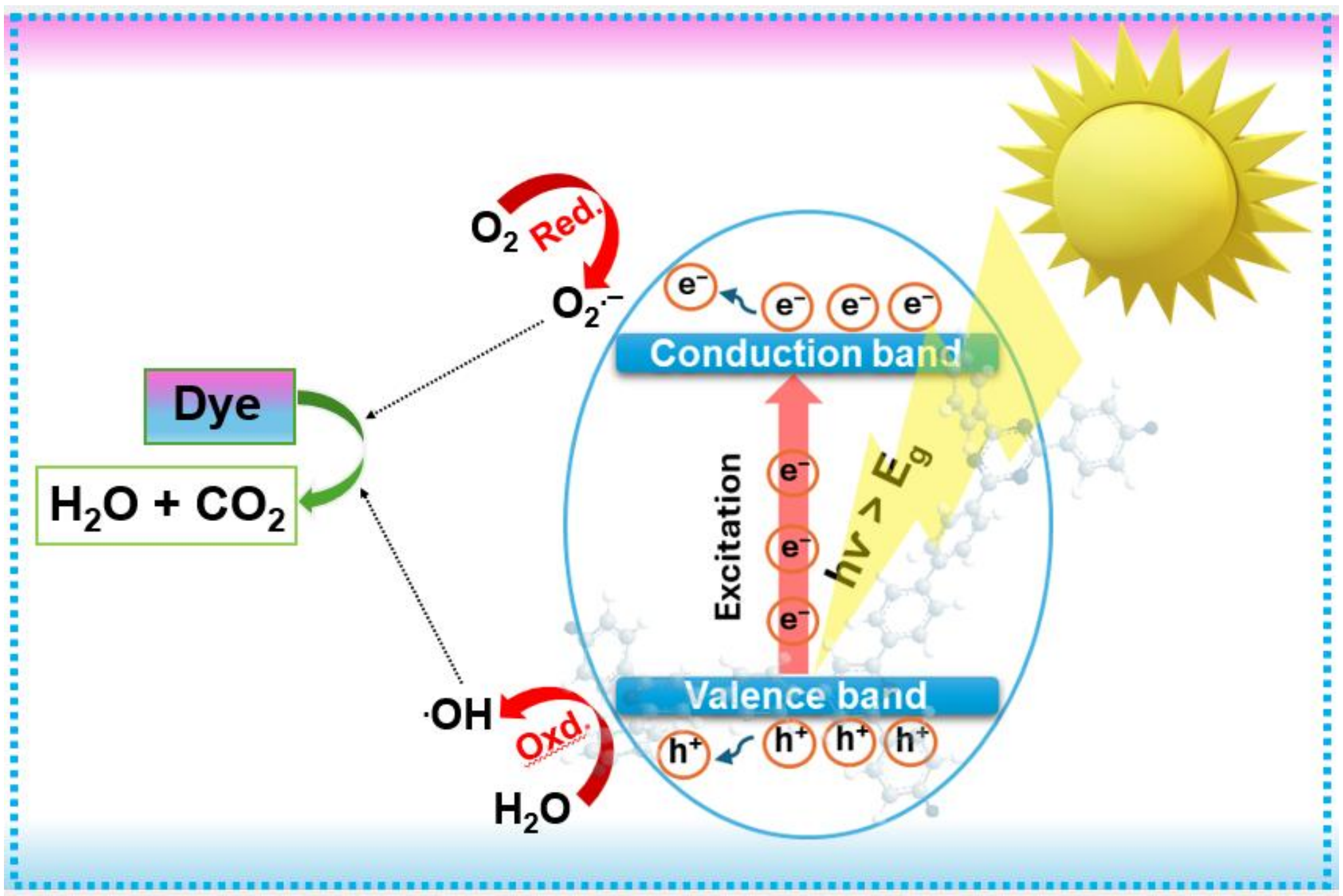

Figure S42. Photodegradation mechanism of RhB and MB using BTPP-TPA and BTPP-TPT CMP photocatalysts.

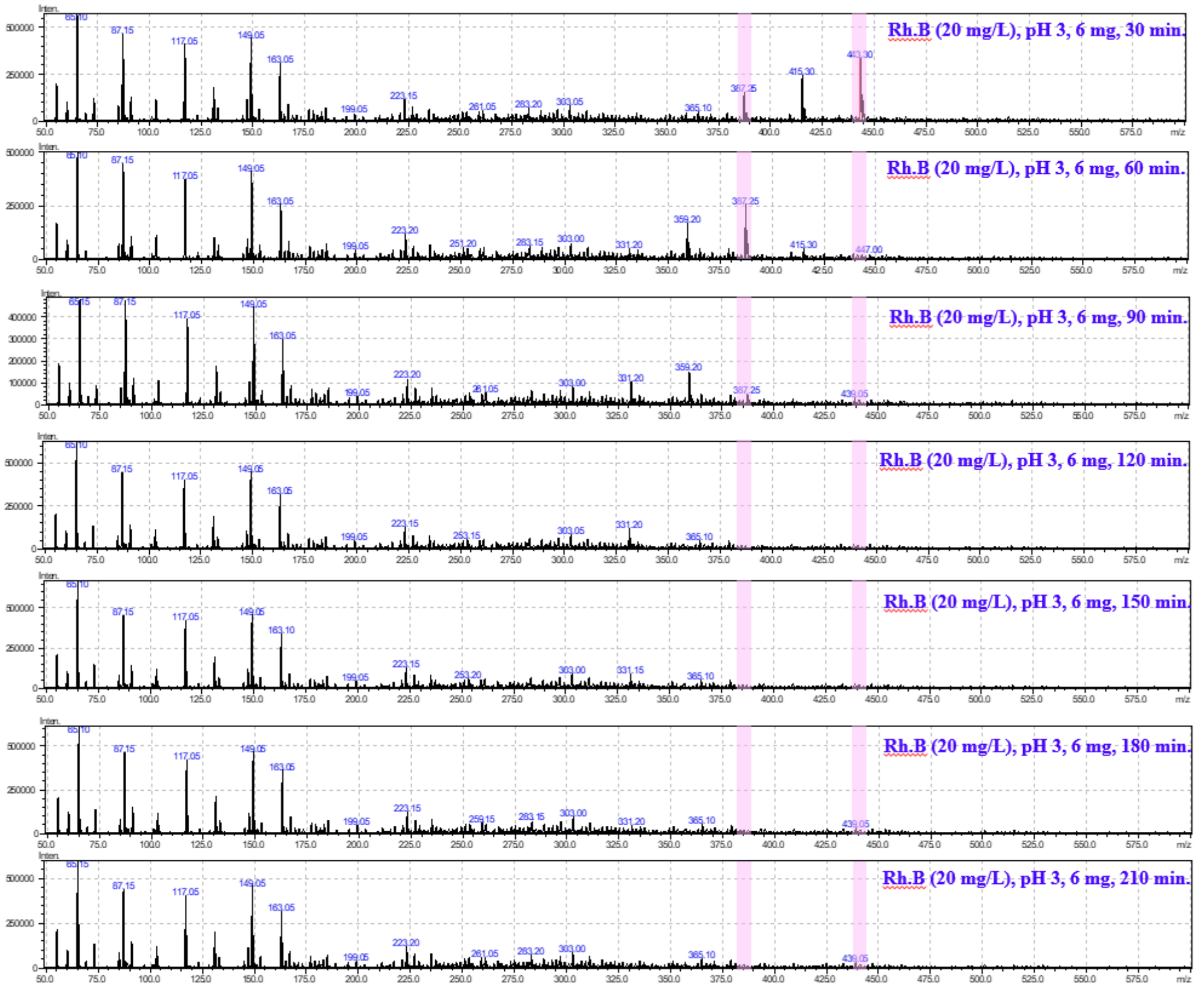

Figure S43. LCMass analysis of photodegradation of RhB over the BTPP-TPT CMP under irradiation with visible light.

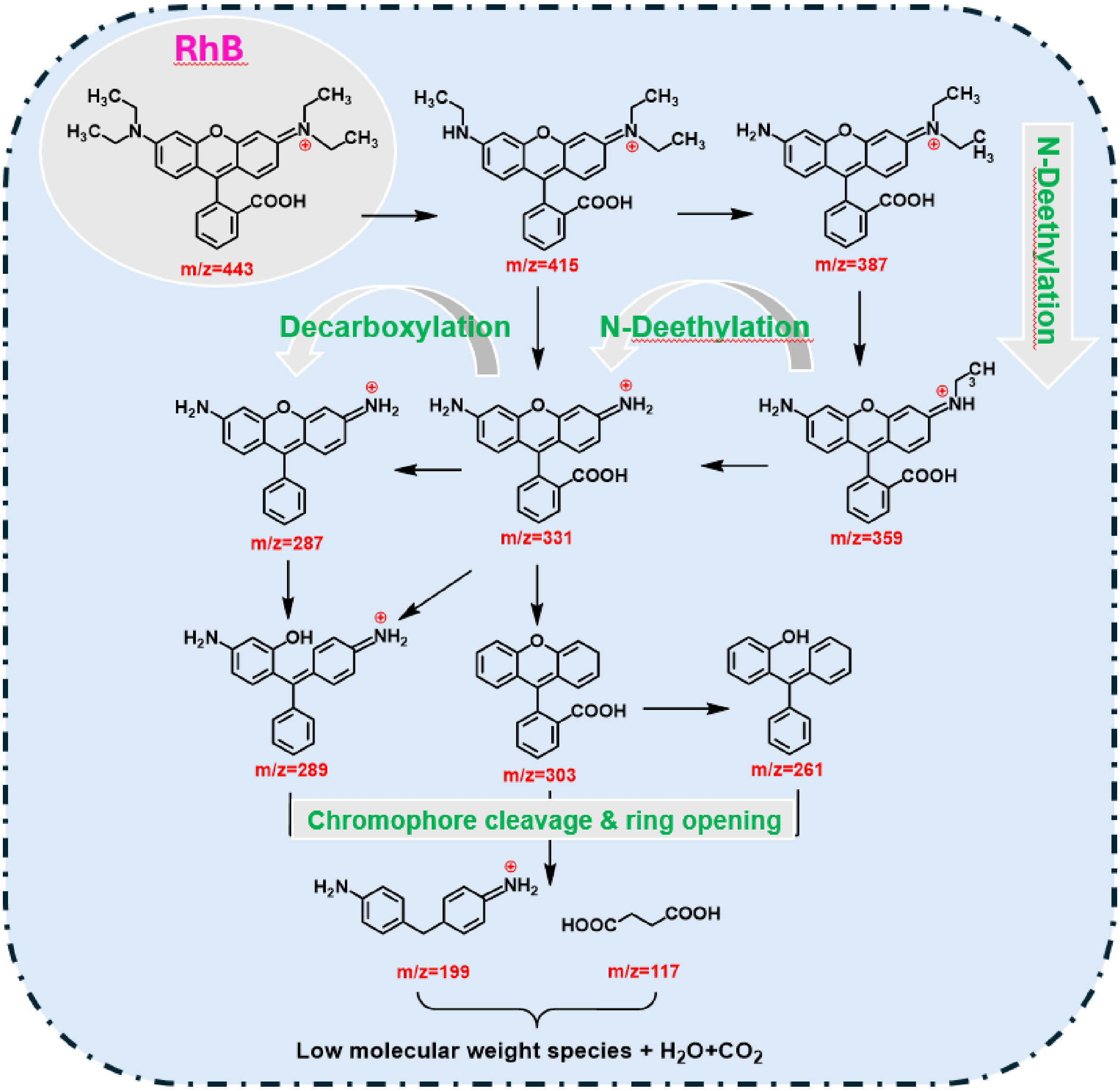

Scheme S6. Photodegradation pathways of RhB over the BTPP-TPT CMP under irradiation with visible light.

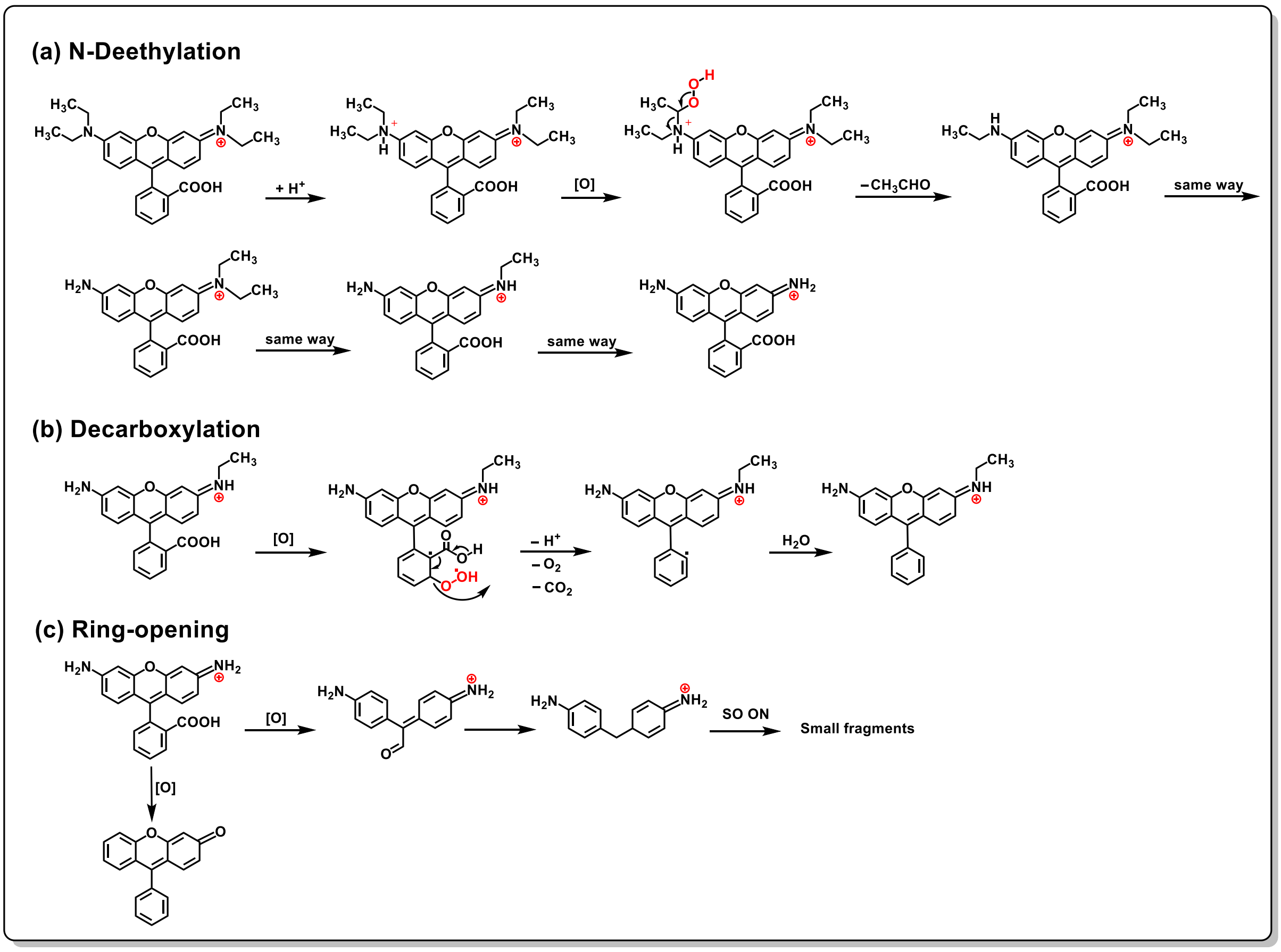

Scheme S7. The expected pathways for the formation of RhB intermediate products using active species. (Sources: Separation and Purification Technology 356 (2025) 129950; Advanced Sensor and Energy Materials 2 (2023) 100054; Nanoscale 11 (2019) 21128–21136; Journal of Hazardous Materials 317 (2016) 17–26; Chemical Engineering Journal 169 (2011) 126–134; J. Phys. Chem. A. 113 (2009) 10024-10032).

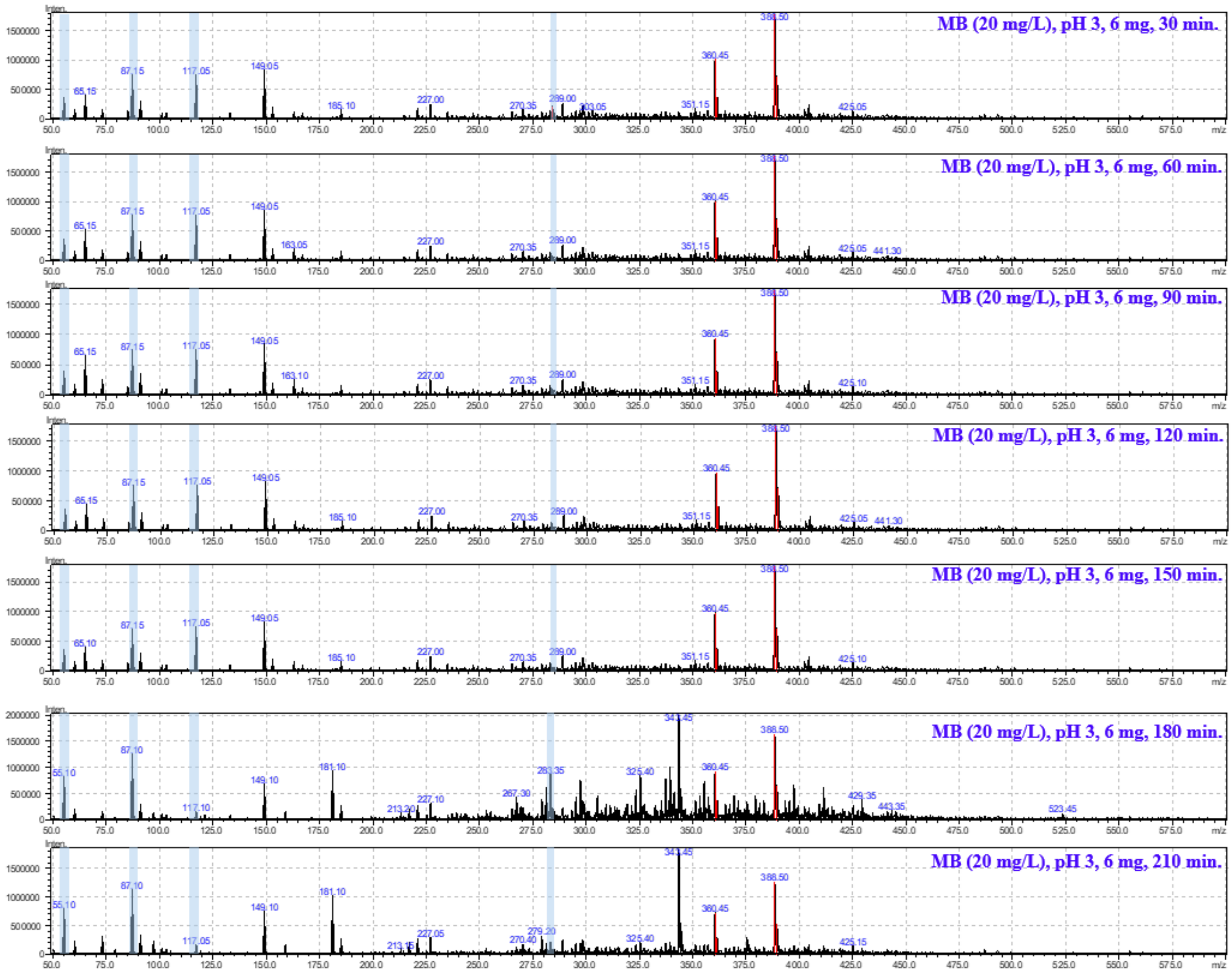

Figure S44. LCMass analysis of photodegradation of MB over the BTPP-TPT CMP under irradiation with visible light. (Peak at m/z = 325.4, 343.45, 360.45, 388.50, 429.35, 523.45) are from background of machine).

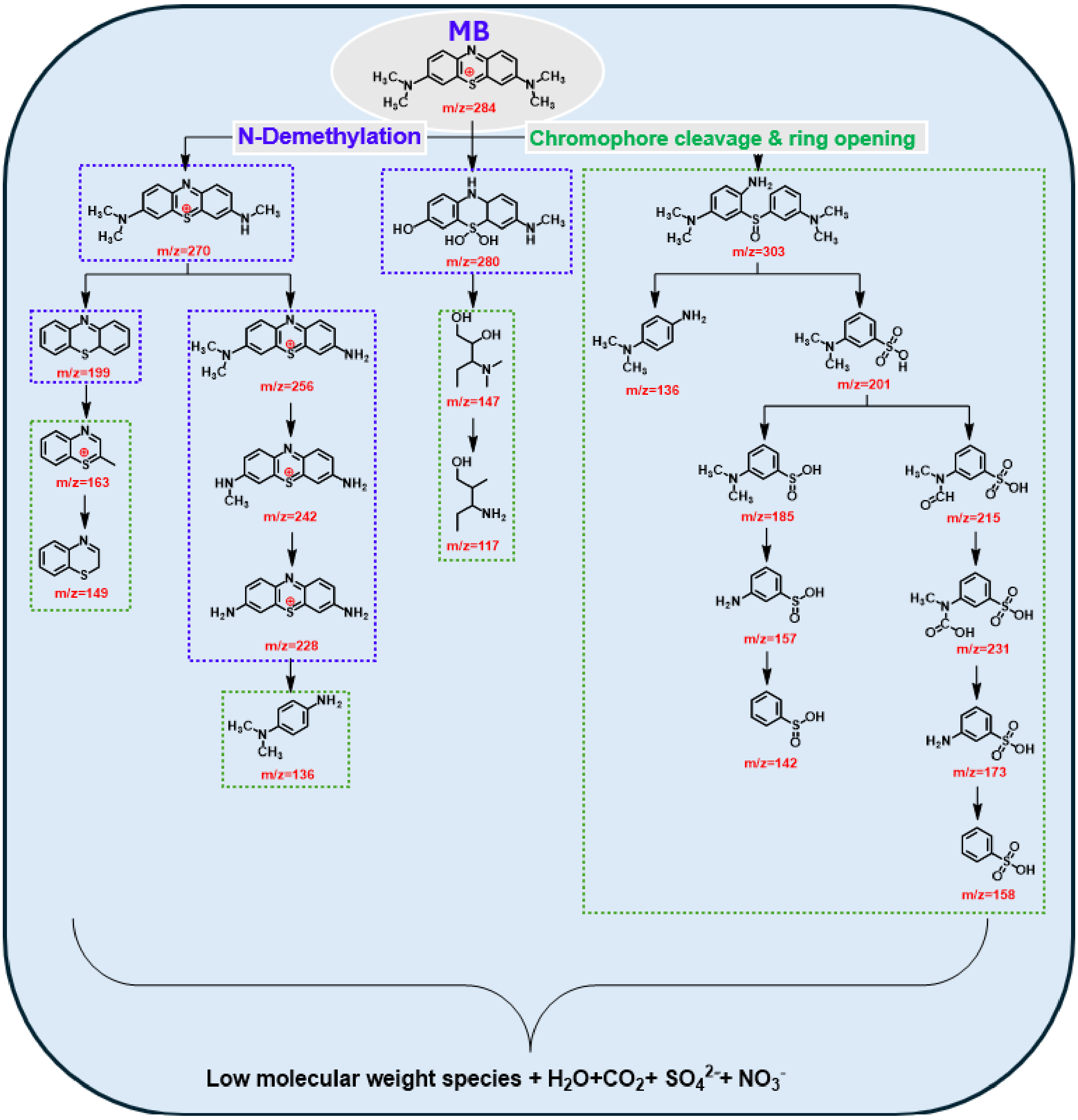

Scheme S8. Photodegradation pathways of MB over the BTPP-TPT CMP under irradiation with visible light.

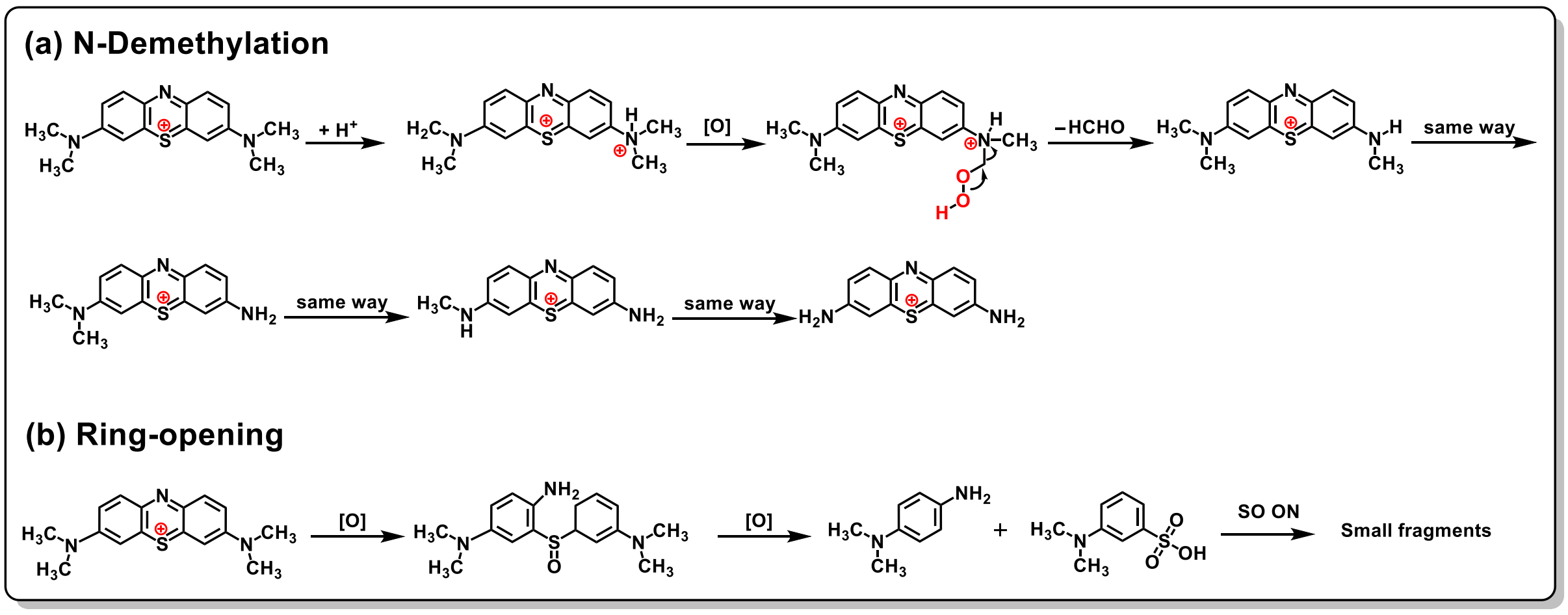

Scheme S9. The expected pathways for the formation of MB intermediate products using active species. (Sources: Separation and Purification Technology 356 (2025) 129950; Water 14 (2022) 242; Journal of Hazardous Materials 400 (2020) 123314; Journal of Polymers and the Environment (2018) 26:3389–3396; Ind. Eng. Chem. Res. 53 (2014) 643-649; Applied Catalysis B: Environmental 31 (2001) 145–157).

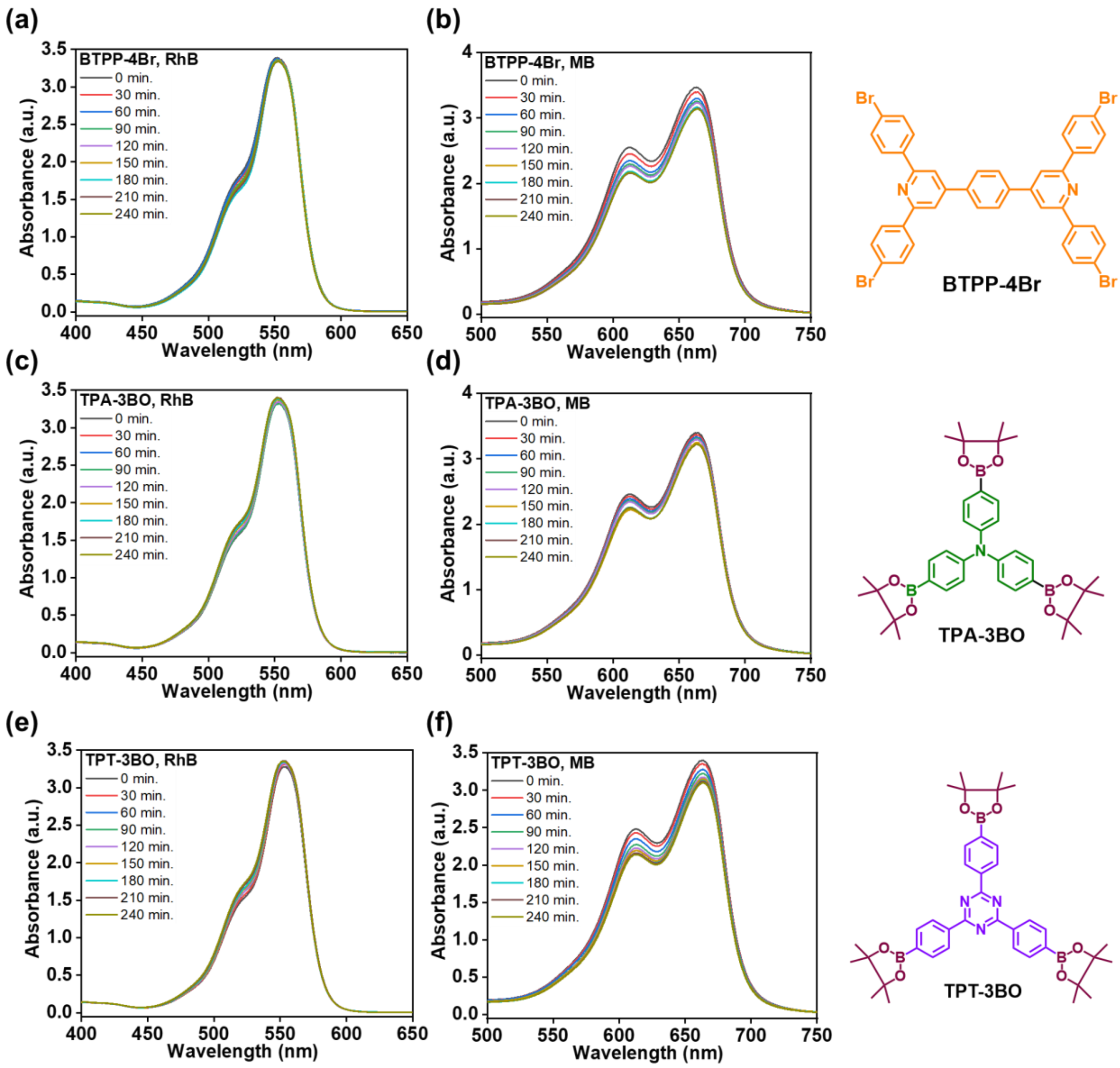

Figure S45. UV–Vis spectra showing the photocatalytic degradation of RhB and MB at different irradiation times in the presence of the individual building blocks: (a,b) BTPP–4Br, (c,d) TPA–3BO, and (e,f) TPT–3BO.

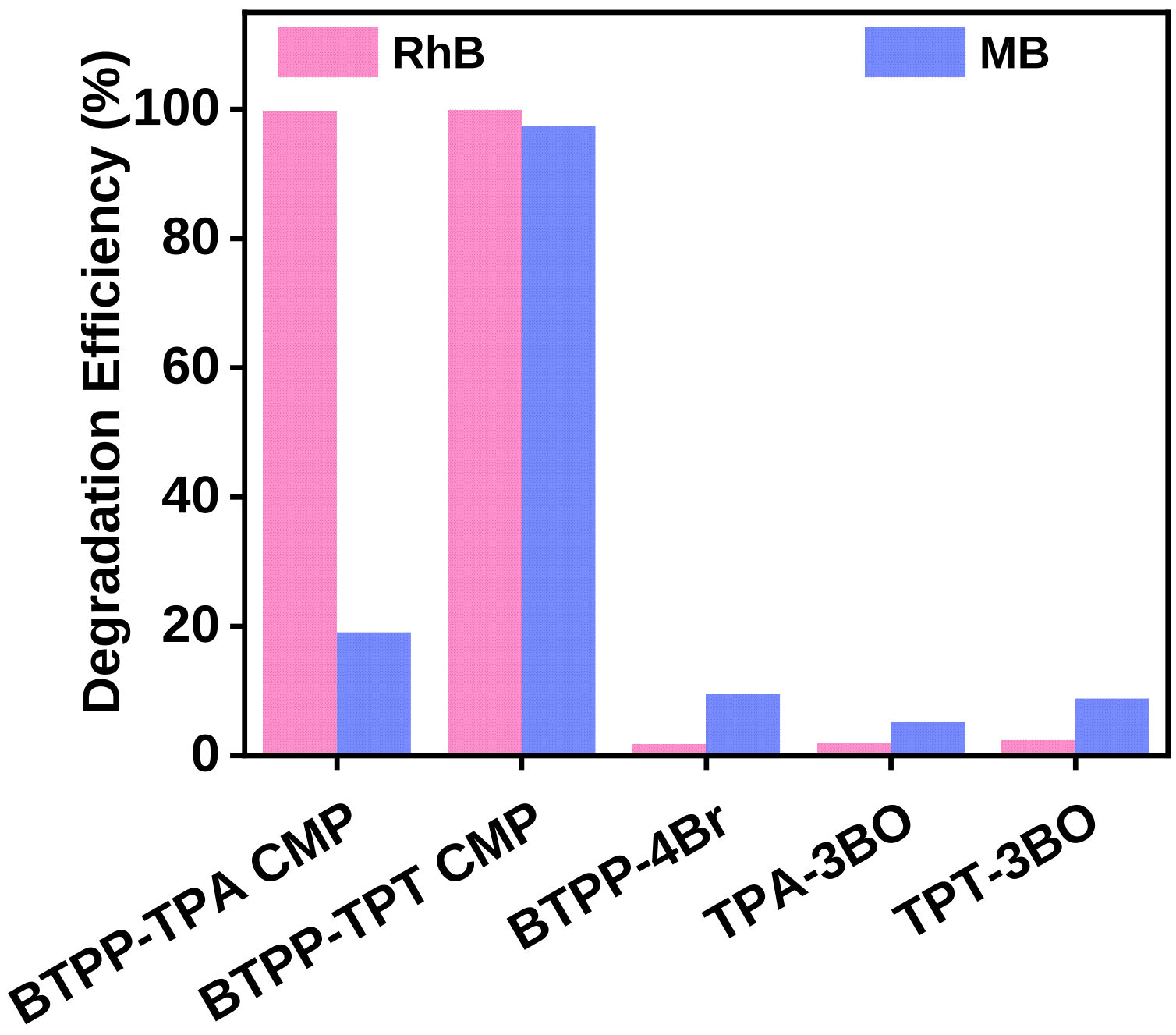

Figure S46. Comparison of the photodegradation performance of BTPP–TPA CMP, BTPP–TPT CMP, BTPP–4Br, TPA–3BO, and TPT–3BO toward RhB and MB under visible-light irradiation.

Table S12. Photodegradation performance of BTPP CMPs, compared with other reported pyridine-based materials.

Catalyst	Catalyst amount (mg)	Dye solution	Degradation time (min)	Degradation efficiency (%)	Ref.
[Zn(pdh)] polymer	40	MB, 80 mL	70	66.95 %	Spectrochimica Acta Part A: Molecular and Biomolecular Spectroscopy 233 (2020) 118232
Fe2O3@PNH	10	Tetracycline, 50 mg L^{-1}, 100 mL	60	94 %	ACS Appl. Nano Mater. 2020, 3, 9200-9208
OCN/Py system	5	Tetracycline, 10 mg L^{-1}, 30 mL	60	97 %	Separation and Purification Technology 314 (2023) 123565
LDPO-1	7	bisphenol A, 14 mL	20	< 50 %	Chem. Commun., 2023, 59, 14399–14402
BTPP-TPA CMP	6	MB, 20 mg L^{-1}, 60 mL	60	6.88 %	**This work**
BTPP-TPT CMP	6	MB, 20 mg L^{-1}, 60 mL	60	65.03 %	**This work**
BTPP-TPA CMP	6	RhB, 20 mg L^{-1}, 60 mL	60	90.79 %	**This work**
BTPP-TPT CMP	6	RhB, 20 mg L^{-1}, 60 mL	60	99.31 %	**This work**

S17. References

[1] S. Abdelnaser, S. W. Kuo, A. F. M. EL-Mahdy, Conjugated microporous polymers incorporating pyridine moieties for efficient faradaic supercapacitor energy storage, J. Power Sources 635 (2025) 236535, https://doi.org/10.1016/j.jpowsour.2025.236535.

[2] J. Zhan, A. F. M. EL-Mahdy, Redox-Active Benzodithiophene-4,8-dione-Based conjugated microporous polymers for High-Performance faradaic supercapacitor energy storage, Chem. Eng. J. 473 (2023) 145124, https://doi.org/10.1016/j.cej.2023.145124.

[3] P. Sivakarthik, V. Thangaraj, M. Parthibavarman, A facile and one-pot synthesis of pure and transition metals (M= Co & Ni) doped WO3 nanoparticles for enhanced photocatalytic performance, J. Mater. Sci. Mater. Electron. 28 (2017) 5990–5996, https://doi.org/10.1007/s10854-016-6274-7.

[4] C. Yang, Z. Cheng, G. Divitini, C. Qian, B. Hou, Y. Liao, A Ni or Co single atom anchored conjugated microporous polymer for high-performance photocatalytic hydrogen evolution, J. Mater. Chem. A 9 (2021) 19894–19900, https://doi.org/10.1039/d1ta02547c.
